# Supplementary figures and images for: Synthesis of geological data and comparative phylogeography of lowland tetrapods suggests recent dispersal through lowland portals crossing the Eastern Andean Cordillera
Source: PeerJ. 2022 Jul 13;10:e13186. doi: 10.7717/peerj.13186 (PMC9288170; doi:10.7717/peerj.13186)

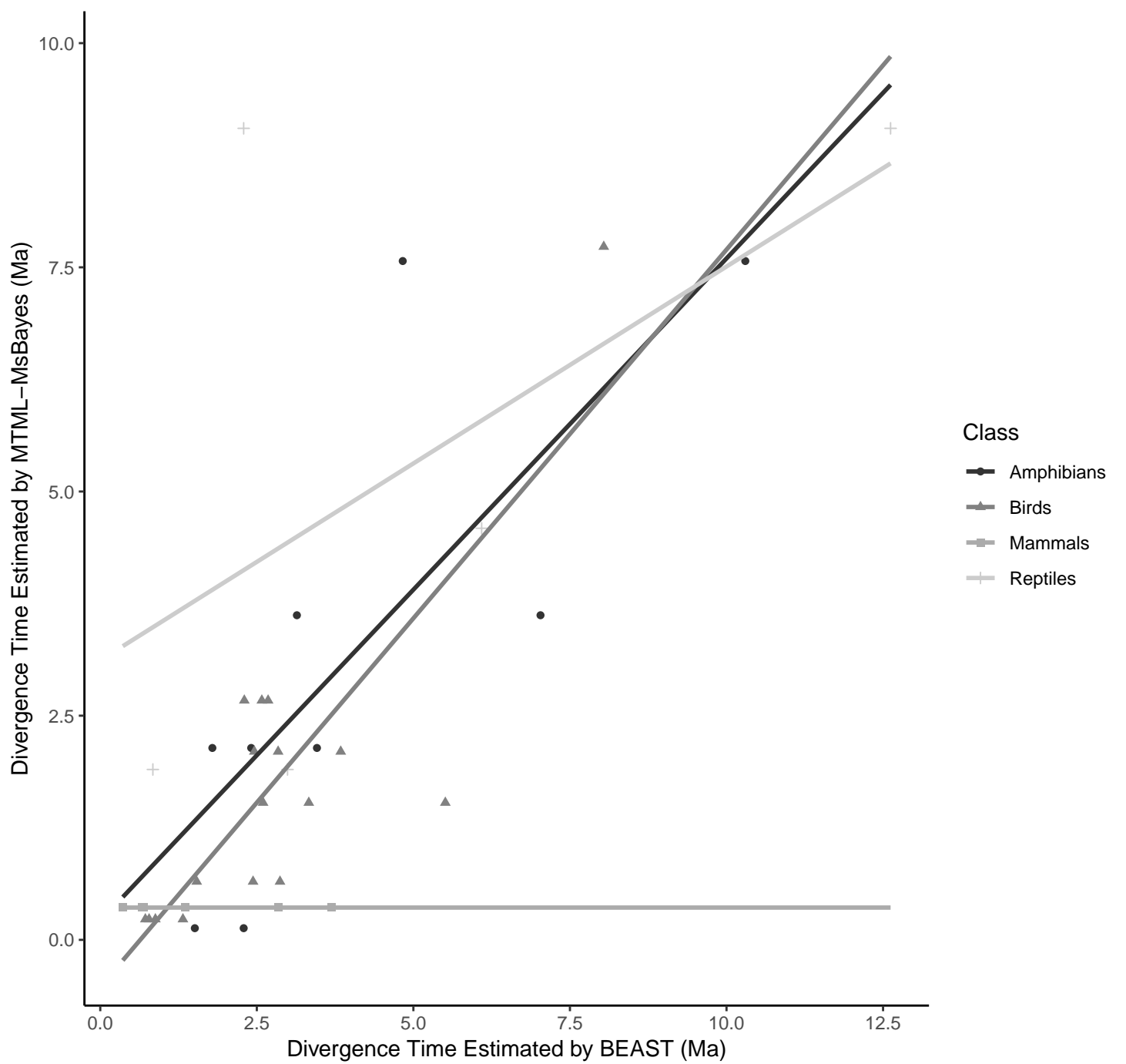

Supplement: Supplemental Information 2 [file peerj-10-13186-s002.pdf]
