# Supplementary material for: Synthesis of geological data and comparative phylogeography of lowland tetrapods suggests recent dispersal through lowland portals crossing the Eastern Andean Cordillera
Source: PeerJ. 2022 Jul 13;10:e13186. doi: 10.7717/peerj.13186 (PMC9288170; doi:10.7717/peerj.13186)
Supplement: Supplemental Information 3 [file peerj-10-13186-s003.docx]

Synthesis of geological data and comparative phylogeography of lowland tetrapods suggests recent dispersal through lowland portals

crossing the Eastern Andean Cordillera

Erika Rodríguez-Muñoz, Camilo Montes, Fernando J. M. Rojas-Runjaic, and Andrew J. Crawford.

**Appendix S1.** Priors used for estimating divergence times independently in each clade based on Bayesian MCMC phylogenetic inference. Prior mean and range on divergence times are in units of million years ago (Ma). Range is given as the 2.5% and 97.5% quantiles bounding the central 95% of the prior density. Prior densities on divergence times followed a lognormal distribution. Calibrated nodes are defined as the most recent common ancestor (MRCA) of two extant clades containing the named descendent lineages. SD = standard deviation in log space. Divergence times in birds were not obtained through secondary calibrations, rather we assumed a rate prior with mean of 0.0104 with range (limits of central 95% density) of 0.0094 to 0.0114, based on the avian molecular clock for mitochondrial DNA reported in Weir & Schluter (2008). We estimated divergence times in amphibians with the calibrated nodes below and additionally, we assumed a rate prior with mean of 0.00955 with range from 0.0074 to 0.01225, according to the molecular clock for mitochondrial DNA for anurans (Crawford, 2003). For mammals and reptiles, we only employed the secondary calibrations reported below and we used a broad uniform prior on the clock rate that ranged from 0.00001 to 0.9 (following a suggestion in the BEAST2 manual). For *Crotalus durissus* we calibrated with the oldest fossil of *Sistrurus*, placed in the Late Miocene - Clarendonian, setting the minimum age of this node strictly at 9 Ma (Anderson & Greenbaum, 2012) and the upper limit at 14 Ma. We verified that the MCMC runs reached stationarity by observing the convergence of independent chains in time series plots of each parameter in Tracer v. 1.6 (Rambaut et al., 2014) and by confirming that the effective sample size (ESS) for each parameter was greater than 200. The two independent chains were then combined in LogCombiner v. 2.4.7 and consensus trees were summarized with TreeAnotator v. 2.4.7 (Drummond & Rambaut, 2007) after discarding the initial 40,001 trees as burnin. See main text for details and references.

| **Ingroup clade** | **Calibration**  **(MRCA of these descendents)** | **Mean**  **(In real space)** | **SD** | **Range**  **(central 95% prior density)** | **Reference** |
| --- | --- | --- | --- | --- | --- |
| **Amphibians** | | | | | |
| *Boana boans* | *Aplastodiscus* and *Boana* | 24.7 | 0.12 | 19.4 – 31.0 | Feng *et al.*, 2017 |
| *B. pugnax* | *Aplastodiscus* and *Boana* | 24.7 | 0.12 | 19.4 – 31.0 | Feng *et al.*, 2017 |
| *B. xerophylla*+*p* | *Aplastodiscus* and *Boana* | 24.7 | 0.12 | 19.4 – 31.0 | Feng *et al.*, 2017 |
| *Dendropsophus microcephalus* | *Dendropsophus* and *Pseudis* | 35.3 | 0.068 | 30.6 – 40.2 | Feng *et al.*, 2017 |
| *Elachistocleis ovalis, E. pearsei, E. bicolor* | *(Gastrophryne, Hypopachus)* and *Elachistocleis* | 20.9 | 0.113 | 16.6 – 25.9 | Feng *et al.*, 2017 |
| *Leptodactylus bolivianus* | *Leptodactylus* and *Lithodytes* | 35.5 | 0.09 | 29.6 – 42.5 | Feng *et al.*, 2017 |
| *Leptodactylus fuscus* | *Leptodactylus* and *Lithodytes* | 35.5 | 0.09 | 29.6 – 42.5 | Feng *et al.*, 2017 |
| *Scarthyla vigilans* | Crown age of (*Scarthyla*, *Pseudis*) and (*Xenohyla*, *Dendropsophus*) | 35.3 | 0.068 | 30.6 – 40.2 | Feng *et al.*, 2017 |
| *Scinax ruber* | *Scinax* and (*Dendropsophus*, *Pseudis*, *Osteocephalus*, *Hyla*, *Acris*) | 46.2 | 0.05 | 39.4 – 48.6 | Feng *et al.*, 2017 |
| **Mammals** | | | | | |
| *Cebus albifrons,*  *Saimiri sciureus, S. oerstedii* | *Cebus* and *Saimiri* | 14 | 0.072 | 12.1 – 16.1 | Chiou *et al*., 2011 |
| *Coendou prehensilis* | *Coendou* *prehensilis* and (*C.* *mexicanus, C. quichua*, *C. rufescens*) | 9.3 | 0.219 | 2.8 – 6.6 | Voss *et al.*, 2013 |
|  | *Erethizon and (C. prehensilis, C.* *mexicanus, C. quichua*, *C. rufescens.* | 6.3 | 0.191 | 5.91 – 13.9 | Voss *et al.*, 2013 |
| *Marmosa robinsoni* | *Marmosa* and *Monodelphis* | 12.0 | 0.083 | 10.2 – 14.1 | Jansa *et al.*, 2014 |
| *Philander opossum* | *Didelphis* and *Philander* | 3.3 | 0.14 | 2.48 – 4.3 | Jansa *et al.*, 2014 |
| *Trachops cirrhosus* | (*Tonatia*, *Macrophyllum*) and *Trachops* | 18.3 | 0.103 | 14.9 – 22.3 | Hoffmann *et al.*, 2008 |
|  | *Macrophyllum* and *Trachops* | 16.9 | 0.114 | 13.4 – 21 |  |
| **Non-avian reptiles** | | | | | |
| *Boa constrictor* | Crown age of Boinae *(Boa* and *[Epicrates, Corallus])* | 60.2 | 0.012 | 58.7 – 61.7 | Head *et al.*, 2009; Hsiang *et al.*, 2015 |
| *Caiman crocodilus* | *Paleosuchus* and *Caiman* | 24.17 | 0.092 | 20.14 – 28.81 | Oaks, 2011 |
| *Chelonoidis carbonaria* | *Chelonoidis denticulata* and *C. carbonaria* | 13.75 | 0.043 | 12.6 – 14.9 | Vargas-Ramírez *et al.*, 2010 |
| *Crotalus durissus* | *Sistrurus* and *Crotalus* | 0.01  Offset 9* | 1 | 9.2 – 14.2 | Anderson & Greenbaum, 2012 |
| *Leptodeira annulata* | *Imantodes* and *Leptodeira* | 15.9 | 0.162 | 20.6 – 30.4 | Daza *et al.*, 2009 |

Anderson, C. G., & Greenbaum, E. (2012). Phylogeography of Northern Populations of the Black-Tailed Rattlesnake (*Crotalus molossus* Baird And Girard, 1853), with the Revalidation of *C. ornatus* Hallowell, 1854. *Herpetological Monographs*, *26*(1), 19–57. https://doi.org/10.1655/HERPMONOGRAPHS-D-11-00012.1

Chiou, K. L., Pozzi, L., Lynch Alfaro, J. W., & Di Fiore, A. (2011). Pleistocene diversification of living squirrel monkeys (*Saimiri spp.*) inferred from complete mitochondrial genome sequences. *Molecular Phylogenetics and Evolution*, *59*(3), 736–745. https://doi.org/10.1016/j.ympev.2011.03.025

Daza, J. M., Smith, E. N., Páez, V. P., & Parkinson, C. L. (2009). Complex evolution in the Neotropics: The origin and diversification of the widespread genus *Leptodeira* (Serpentes: Colubridae). *Molecular Phylogenetics and Evolution*, *53*(3), 653–667. https://doi.org/10.1016/j.ympev.2009.07.022

Eo, S. H., & DeWoody, J. A. (2010). Evolutionary rates of mitochondrial genomes correspond to diversification rates and to contemporary species richness in birds and reptiles. *Proceedings of the Royal Society B: Biological Sciences*, *277*(1700), 3587–3592. https://doi.org/10.1098/rspb.2010.0965

Feng, Y.-J., Blackburn, D. C., Liang, D., Hillis, D. M., Wake, D. B., Cannatella, D. C., & Zhang, P. (2017). Phylogenomics reveals rapid, simultaneous diversification of three major clades of Gondwanan frogs at the Cretaceous–Paleogene boundary. *Proceedings of the National Academy of Sciences*, *114*(29), E5864–E5870. https://doi.org/10.1073/pnas.1704632114

Green, R. E., Braun, E. L., Armstrong, J., Earl, D., Nguyen, N., Hickey, G., … Ray, D. A. (2014). Three crocodilian genomes reveal ancestral patterns of evolution among archosaurs. *Science*, *346*(6215), 1254449–1254449. https://doi.org/10.1126/science.1254449

Head, J. J., Bloch, J. I., Hastings, A. K., Bourque, J. R., Cadena, E. A., Herrera, F. A., … Jaramillo, C. A. (2009). Giant boid snake from the Palaeocene neotropics reveals hotter past equatorial temperatures. *Nature*, *457*(7230), 715–717. https://doi.org/10.1038/nature07671

Hoffmann, F. G., Hoofer, S. R., & Baker, R. J. (2008). Molecular dating of the diversification of Phyllostominae bats based on nuclear and mitochondrial DNA sequences. *Molecular Phylogenetics and Evolution*, *49*(2), 653–658. https://doi.org/10.1016/j.ympev.2008.08.002

Jansa, S. A., Barker, F. K., & Voss, R. S. (2014). The early diversification history of didelphid marsupials: A window into south America’s ‘splendid isolation’. *Evolution*, *68*(3), 684–695. https://doi.org/10.1111/evo.12290

Kumar, S., & Subramanian, S. (2002). Mutation rates in mammalian genomes. *Proceedings of the National Academy of Sciences*, *99*, 803–808. https://doi.org/10.1073/pnas.022629899

Vargas-Ramírez, M., Maran, J., & Fritz, U. (2010). Red- and yellow-footed tortoises, *Chelonoidis carbonaria* and *C. denticulata* (Reptilia: Testudines: Testudinidae), in South American savannahs and forests: do their phylogeographies reflect distinct habitats? *Organisms Diversity and Evolution*, *10*, 161–172. https://doi.org/10.1007/s13127-010-0016-0

Voss, R. S., Hubbard, C., & Jansa, S. A. (2013). Phylogenetic relationships of new world porcupines (Rodentia, Erethizontidae): Implication for taxonomy, morphological evolution, and biogeography. *American Museum Novitates*, (3769), 1–36. https://doi.org/10.1206/3769.2

Weir, J. T., & Schluter, D. (2008). Calibrating the avian molecular clock. *Molecular Ecology*, *17*, 2321–2328. https://doi.org/10.1111/j.1365-294X.2008.03742.x
