# Supplementary material for: Synthesis of geological data and comparative phylogeography of lowland tetrapods suggests recent dispersal through lowland portals crossing the Eastern Andean Cordillera
Source: PeerJ. 2022 Jul 13;10:e13186. doi: 10.7717/peerj.13186 (PMC9288170; doi:10.7717/peerj.13186)
Supplement: Supplemental Information 4 [file peerj-10-13186-s004.pdf]

Rodríguez-Muñoz, Montes & Crawford. *Synthesis of geological and comparative phylogeographic data rejects mountain uplift as driver of lowland divergence across the Eastern Andean Cordillera*

Appendix S2. Calibrated trees made with BEAST. Decimal values in the nodes represent the posterior values of support. Samples from the eastern and western sides of the EC-MA are represented with "E" and "W", respectively. The red point indicates the age of divergence between eastern and western populations and represent the nodes used for hABC analyses.

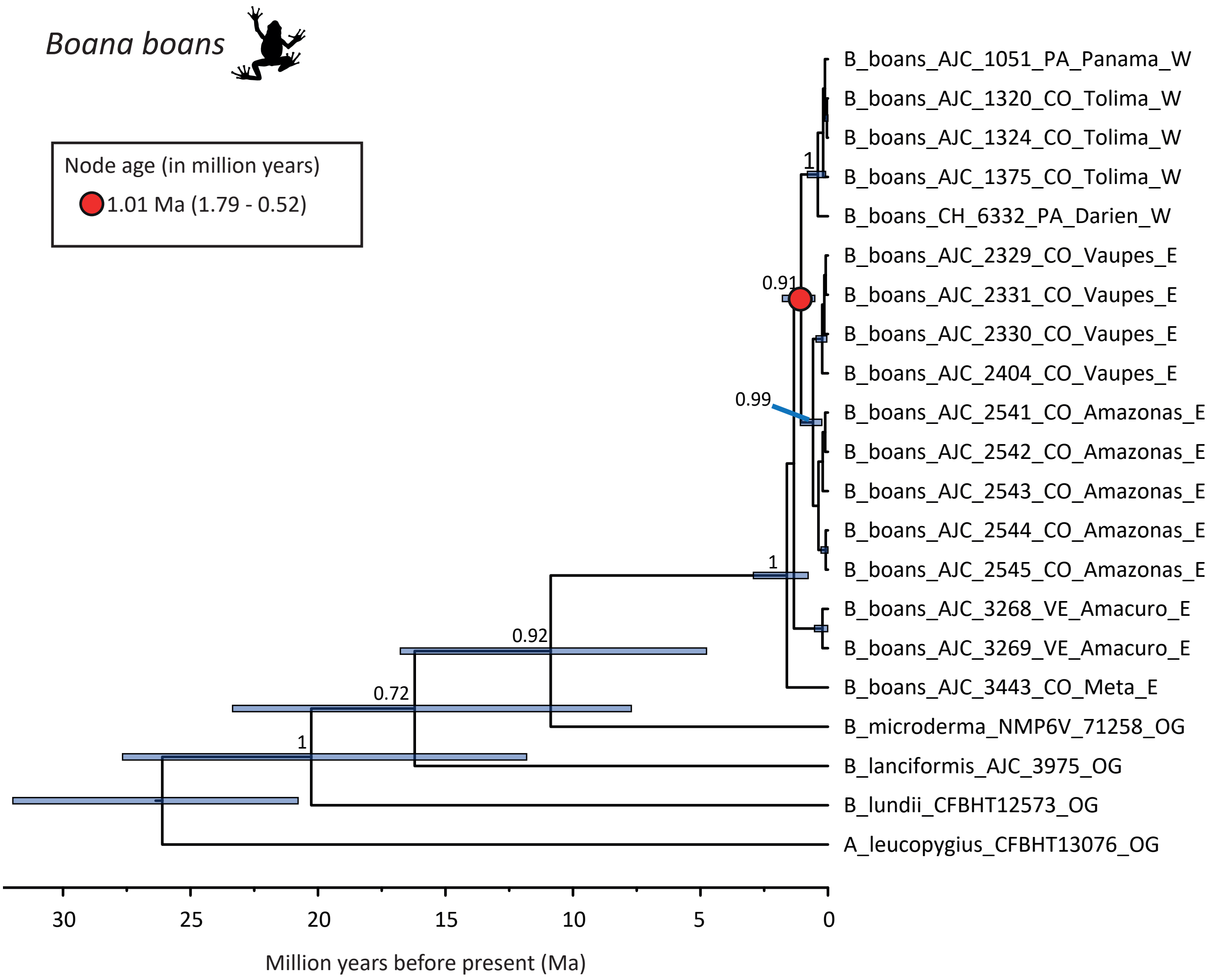

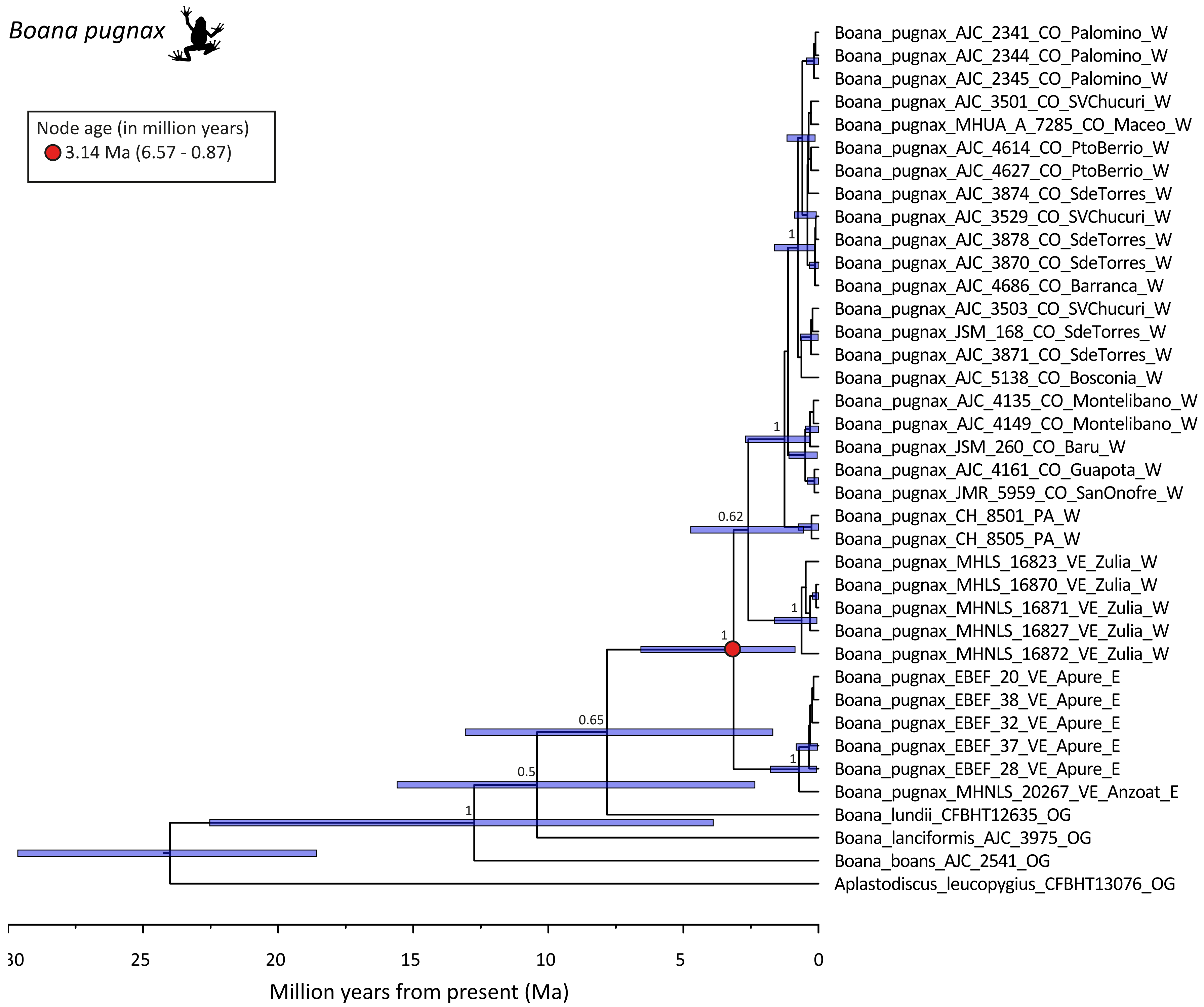

Boana pugnax
Node age (in million years)
3.14 Ma (6.57 - 0.87)
Boana_pugnax_AJC_2341_CO_Palomino_W
Boana_pugnax_AJC_2344_CO_Palomino_W
Boana_pugnax_AJC_2345_CO_Palomino_W
Boana_pugnax_AJC_3501_CO_SVChucuri_W
Boana_pugnax_MHUA_A_7285_CO_Maceo_W
Boana_pugnax_AJC_4614_CO_PtoBerrio_W
Boana_pugnax_AJC_4627_CO_PtoBerrio_W
Boana_pugnax_AJC_3874_CO_SdeTorres_W
Boana_pugnax_AJC_3529_CO_SVChucuri_W
Boana_pugnax_AJC_3878_CO_SdeTorres_W
Boana_pugnax_AJC_3870_CO_SdeTorres_W
Boana_pugnax_AJC_4686_CO_Barranca_W
Boana_pugnax_AJC_3503_CO_SVChucuri_W
Boana_pugnax_JSM_168_CO_SdeTorres_W
Boana_pugnax_AJC_3871_CO_SdeTorres_W
Boana_pugnax_AJC_5138_CO_Bosconia_W
Boana_pugnax_AJC_4135_CO_Montelibano_W
Boana_pugnax_AJC_4149_CO_Montelibano_W
Boana_pugnax_JSM_260_CO_Baru_W
Boana_pugnax_AJC_4161_CO_Guapota_W
Boana_pugnax_JMR_5959_CO_SanOnofre_W
Boana_pugnax_CH_8501_PA_W
Boana_pugnax_CH_8505_PA_W
Boana_pugnax_MHLS_16823_VE_Zulia_W
Boana_pugnax_MHLS_16870_VE_Zulia_W
Boana_pugnax_MHNLS_16871_VE_Zulia_W
Boana_pugnax_MHNLS_16827_VE_Zulia_W
Boana_pugnax_MHNLS_16872_VE_Zulia_W
Boana_pugnax_EBEF_20_VE_Apure_E
Boana_pugnax_EBEF_38_VE_Apure_E
Boana_pugnax_EBEF_32_VE_Apure_E
Boana_pugnax_EBEF_37_VE_Apure_E
Boana_pugnax_EBEF_28_VE_Apure_E
Boana_pugnax_MHNLS_20267_VE_Anzoat_E
Boana_lundii_CFBHT12635_OG
Boana_lanciformis_AJC_3975_OG
Boana_boans_AJC_2541_OG
Aplastodiscus_leucopygius_CFBHT13076_OG
1
1
0.62
1
1
0.65
1
0.5
1
30
25
20
15
10
5
0
Million years from present (Ma)

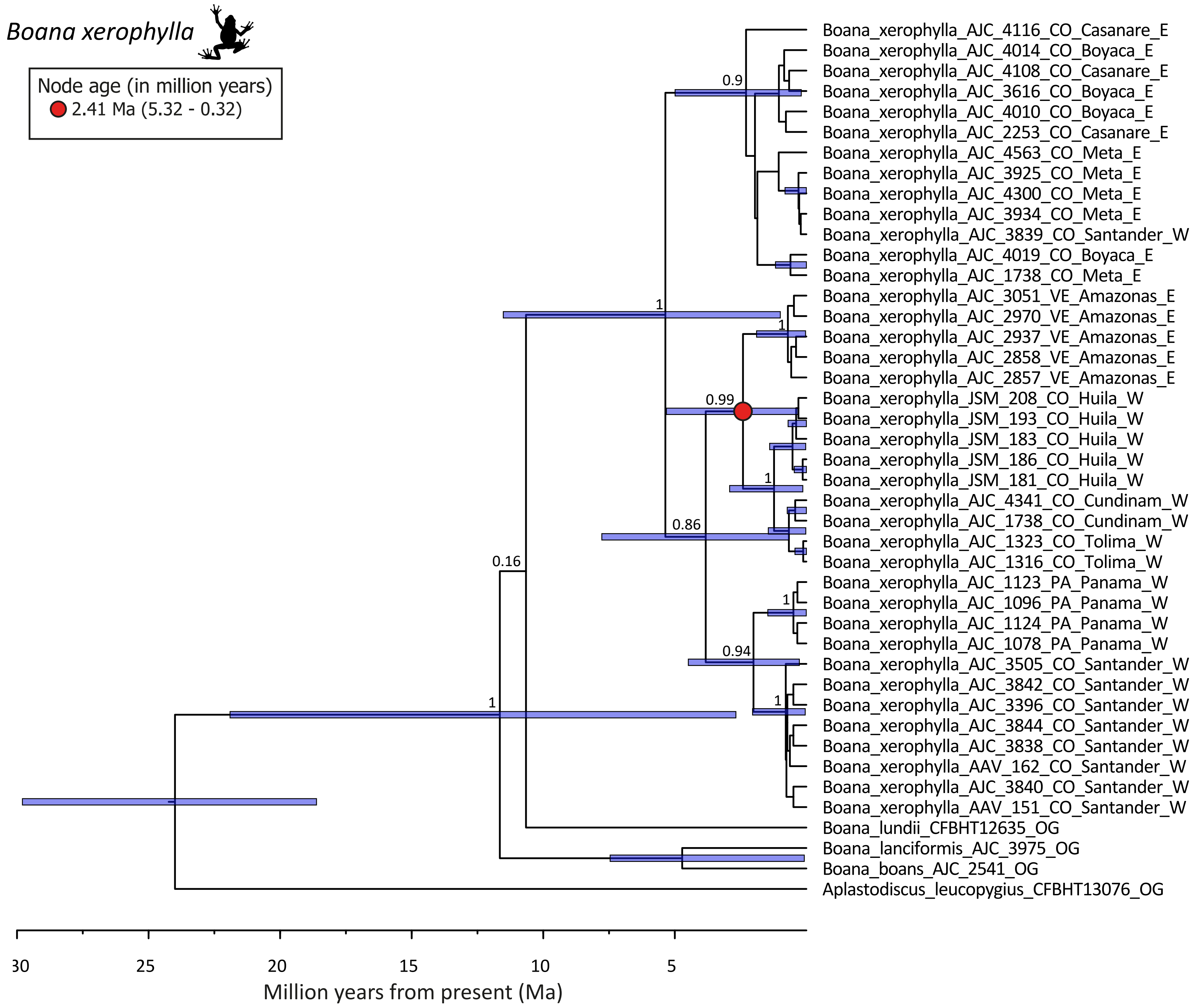
Boana xerophylla
Node age (in million years)
2.41 Ma (5.32 - 0.32)
0.9
1
1
0.99
1
0.86
0.16
1
0.94
1
1
Boana_xerophylla_AJC_4116_CO_Casanare_E
Boana_xerophylla_AJC_4014_CO_Boyaca_E
Boana_xerophylla_AJC_4108_CO_Casanare_E
Boana_xerophylla_AJC_3616_CO_Boyaca_E
Boana_xerophylla_AJC_4010_CO_Boyaca_E
Boana_xerophylla_AJC_2253_CO_Casanare_E
Boana_xerophylla_AJC_4563_CO_Meta_E
Boana_xerophylla_AJC_3925_CO_Meta_E
Boana_xerophylla_AJC_4300_CO_Meta_E
Boana_xerophylla_AJC_3934_CO_Meta_E
Boana_xerophylla_AJC_3839_CO_Santander_W
Boana_xerophylla_AJC_4019_CO_Boyaca_E
Boana_xerophylla_AJC_1738_CO_Meta_E
Boana_xerophylla_AJC_3051_VE_Amazonas_E
Boana_xerophylla_AJC_2970_VE_Amazonas_E
Boana_xerophylla_AJC_2937_VE_Amazonas_E
Boana_xerophylla_AJC_2858_VE_Amazonas_E
Boana_xerophylla_AJC_2857_VE_Amazonas_E
Boana_xerophylla_JSM_208_CO_Huila_W
Boana_xerophylla_JSM_193_CO_Huila_W
Boana_xerophylla_JSM_183_CO_Huila_W
Boana_xerophylla_JSM_186_CO_Huila_W
Boana_xerophylla_JSM_181_CO_Huila_W
Boana_xerophylla_AJC_4341_CO_Cundinam_W
Boana_xerophylla_AJC_1738_CO_Cundinam_W
Boana_xerophylla_AJC_1323_CO_Tolima_W
Boana_xerophylla_AJC_1316_CO_Tolima_W
Boana_xerophylla_AJC_1123_PA_Panama_W
Boana_xerophylla_AJC_1096_PA_Panama_W
Boana_xerophylla_AJC_1124_PA_Panama_W
Boana_xerophylla_AJC_1078_PA_Panama_W
Boana_xerophylla_AJC_3505_CO_Santander_W
Boana_xerophylla_AJC_3842_CO_Santander_W
Boana_xerophylla_AJC_3396_CO_Santander_W
Boana_xerophylla_AJC_3844_CO_Santander_W
Boana_xerophylla_AJC_3838_CO_Santander_W
Boana_xerophylla_AAV_162_CO_Santander_W
Boana_xerophylla_AJC_3840_CO_Santander_W
Boana_xerophylla_AAV_151_CO_Santander_W
Boana_lundii_CFBHT12635_OG
Boana_lanciformis_AJC_3975_OG
Boana_boans_AJC_2541_OG
Aplastodiscus_leucopygius_CFBHT13076_OG
30
25
20
15
10
5
Million years from present (Ma)

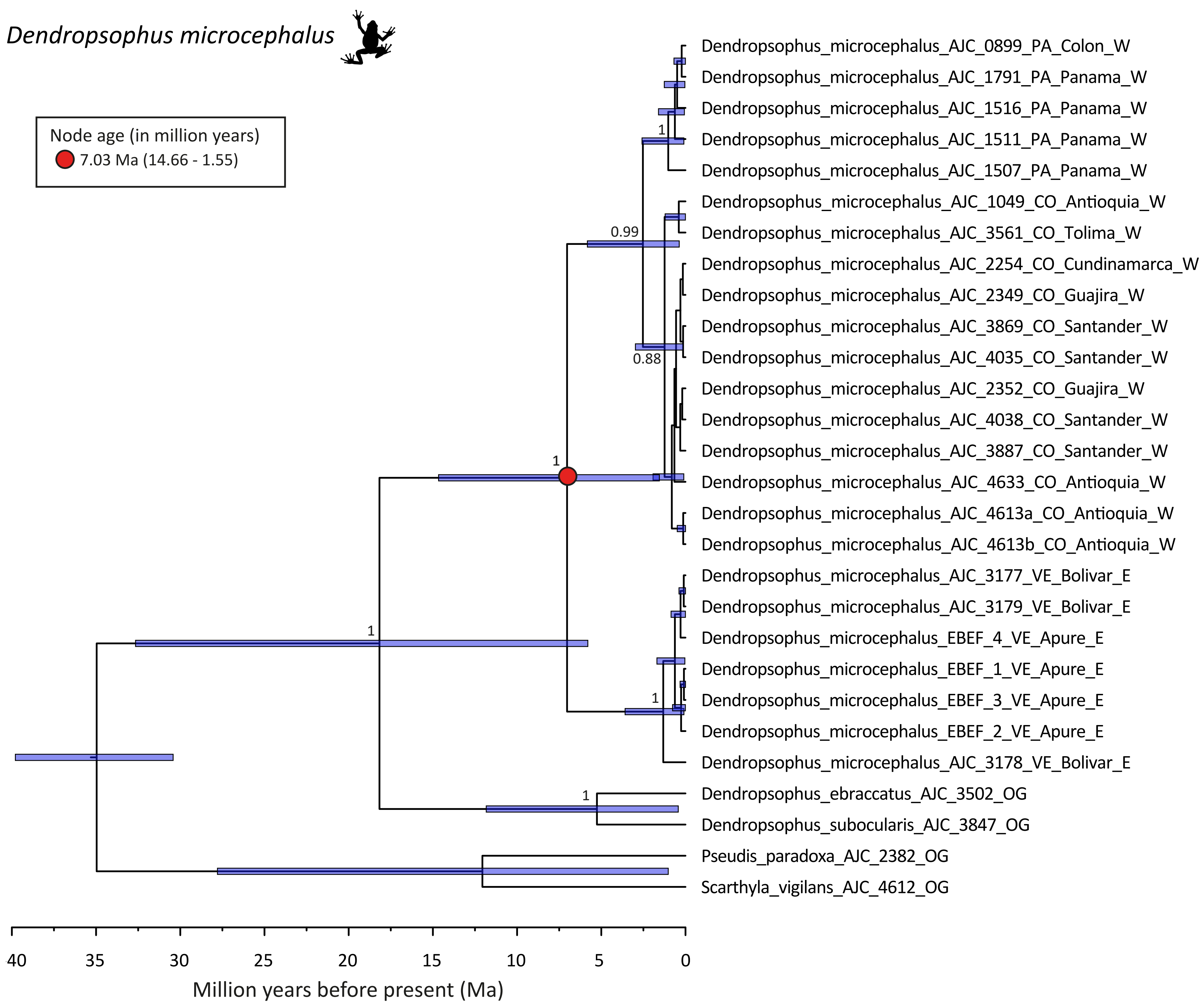

Dendropsophus microcephalus
Node age (in million years)
7.03 Ma (14.66 - 1.55)
Dendropsophus_microcephalus_AJC_0899_PA_Colon_W
Dendropsophus_microcephalus_AJC_1791_PA_Panama_W
Dendropsophus_microcephalus_AJC_1516_PA_Panama_W
Dendropsophus_microcephalus_AJC_1511_PA_Panama_W
Dendropsophus_microcephalus_AJC_1507_PA_Panama_W
Dendropsophus_microcephalus_AJC_1049_CO_Antioquia_W
Dendropsophus_microcephalus_AJC_3561_CO_Tolima_W
Dendropsophus_microcephalus_AJC_2254_CO_Cundinamarca_W
Dendropsophus_microcephalus_AJC_2349_CO_Guajira_W
Dendropsophus_microcephalus_AJC_3869_CO_Santander_W
Dendropsophus_microcephalus_AJC_4035_CO_Santander_W
Dendropsophus_microcephalus_AJC_2352_CO_Guajira_W
Dendropsophus_microcephalus_AJC_4038_CO_Santander_W
Dendropsophus_microcephalus_AJC_3887_CO_Santander_W
Dendropsophus_microcephalus_AJC_4633_CO_Antioquia_W
Dendropsophus_microcephalus_AJC_4613a_CO_Antioquia_W
Dendropsophus_microcephalus_AJC_4613b_CO_Antioquia_W
Dendropsophus_microcephalus_AJC_3177_VE_Bolivar_E
Dendropsophus_microcephalus_AJC_3179_VE_Bolivar_E
Dendropsophus_microcephalus_EBEF_4_VE_Apure_E
Dendropsophus_microcephalus_EBEF_1_VE_Apure_E
Dendropsophus_microcephalus_EBEF_3_VE_Apure_E
Dendropsophus_microcephalus_EBEF_2_VE_Apure_E
Dendropsophus_microcephalus_AJC_3178_VE_Bolivar_E
Dendropsophus_ebraccatus_AJC_3502_OG
Dendropsophus_subocularis_AJC_3847_OG
Pseudis_paradoxa_AJC_2382_OG
Scarthyla_vigilans_AJC_4612_OG
1
0.99
1
0.88
1
1
1
1
40
35
30
25
20
15
10
5
0
Million years before present (Ma)

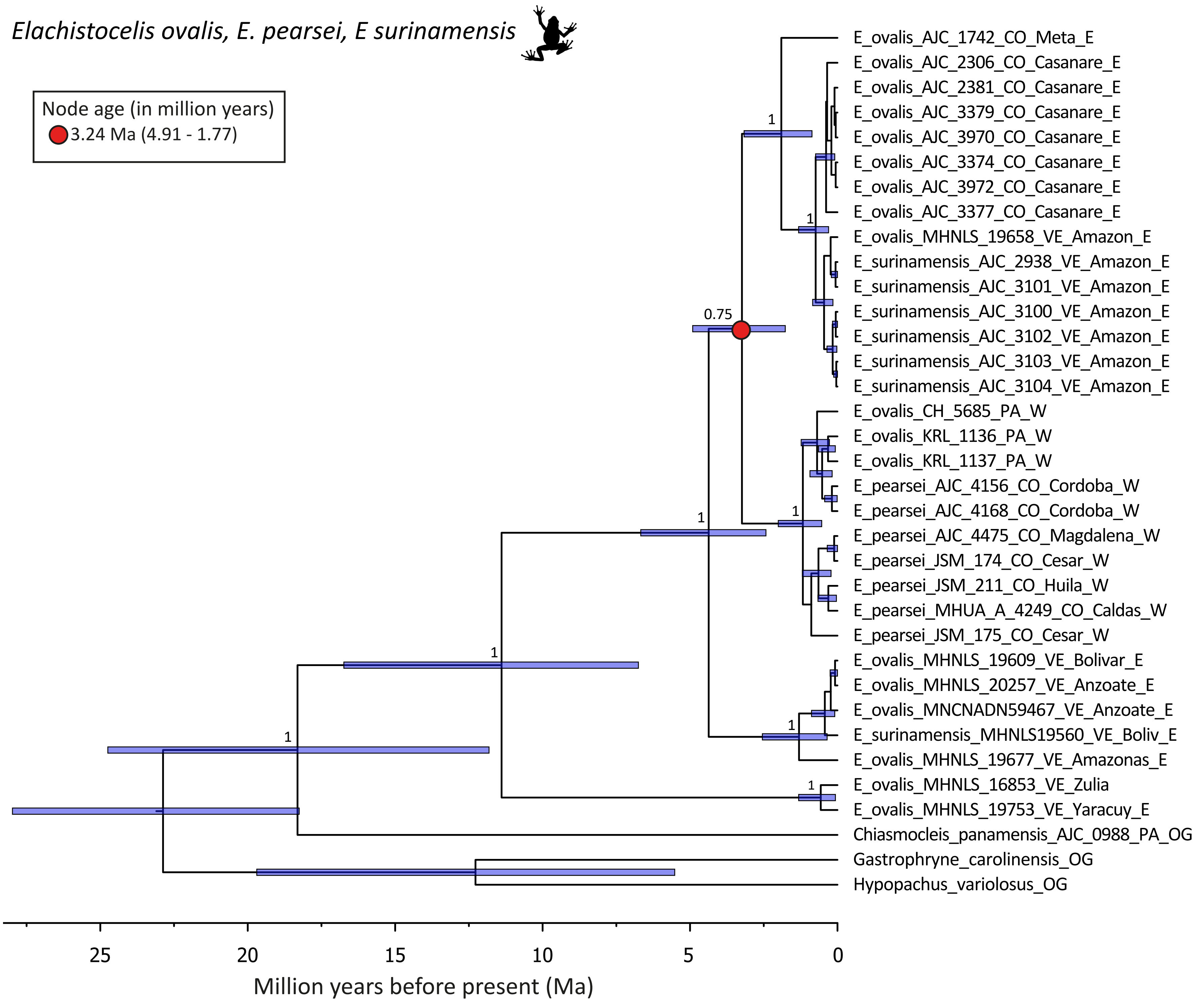
Elachistocelis ovalis, E. pearsei, E surinamensis
Node age (in million years)
3.24 Ma (4.91 - 1.77)
E_ovalis_AJC_1742_CO_Meta_E
E_ovalis_AJC_2306_CO_Casanare_E
E_ovalis_AJC_2381_CO_Casanare_E
E_ovalis_AJC_3379_CO_Casanare_E
E_ovalis_AJC_3970_CO_Casanare_E
E_ovalis_AJC_3374_CO_Casanare_E
E_ovalis_AJC_3972_CO_Casanare_E
E_ovalis_AJC_3377_CO_Casanare_E
E_ovalis_MHNLS_19658_VE_Amazon_E
E_surinamensis_AJC_2938_VE_Amazon_E
E_surinamensis_AJC_3101_VE_Amazon_E
E_surinamensis_AJC_3100_VE_Amazon_E
E_surinamensis_AJC_3102_VE_Amazon_E
E_surinamensis_AJC_3103_VE_Amazon_E
E_surinamensis_AJC_3104_VE_Amazon_E
E_ovalis_CH_5685_PA_W
E_ovalis_KRL_1136_PA_W
E_ovalis_KRL_1137_PA_W
E_pearsei_AJC_4156_CO_Cordoba_W
E_pearsei_AJC_4168_CO_Cordoba_W
E_pearsei_AJC_4475_CO_Magdalena_W
E_pearsei_JSM_174_CO_Cesar_W
E_pearsei_JSM_211_CO_Huila_W
E_pearsei_MHUA_A_4249_CO_Caldas_W
E_pearsei_JSM_175_CO_Cesar_W
E_ovalis_MHNLS_19609_VE_Bolivar_E
E_ovalis_MHNLS_20257_VE_Anzoate_E
E_ovalis_MNCNADN59467_VE_Anzoate_E
E_surinamensis_MHNLS19560_VE_Boliv_E
E_ovalis_MHNLS_19677_VE_Amazonas_E
E_ovalis_MHNLS_16853_VE_Zulia
E_ovalis_MHNLS_19753_VE_Yaracuy_E
Chiasmocleis_panamensis_AJC_0988_PA_OG
Gastrophryne_carolinensis_OG
Hypopachus_variolosus_OG
1
1
0.75
1
1
1
1
1
1
25
20
15
10
5
0
Million years before present (Ma)

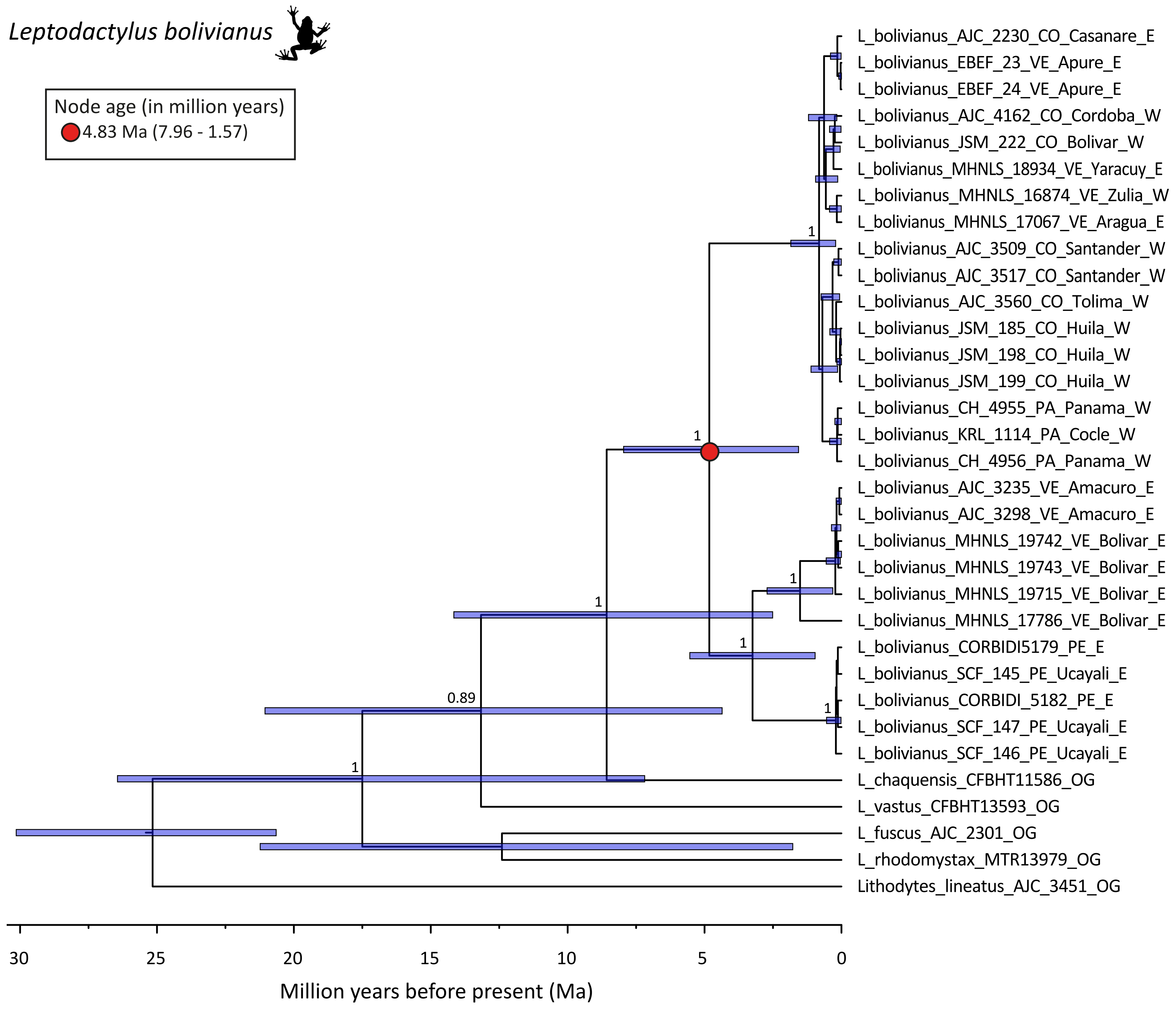
Leptodactylus bolivianus
Node age (in million years)
4.83 Ma (7.96 - 1.57)
L_bolivianus_AJC_2230_CO_Casanare_E
L_bolivianus_EBEF_23_VE_Apure_E
L_bolivianus_EBEF_24_VE_Apure_E
L_bolivianus_AJC_4162_CO_Cordoba_W
L_bolivianus_JSM_222_CO_Bolivar_W
L_bolivianus_MHNLS_18934_VE_Yaracuy_E
L_bolivianus_MHNLS_16874_VE_Zulia_W
L_bolivianus_MHNLS_17067_VE_Aragua_E
L_bolivianus_AJC_3509_CO_Santander_W
L_bolivianus_AJC_3517_CO_Santander_W
L_bolivianus_AJC_3560_CO_Tolima_W
L_bolivianus_JSM_185_CO_Huila_W
L_bolivianus_JSM_198_CO_Huila_W
L_bolivianus_JSM_199_CO_Huila_W
L_bolivianus_CH_4955_PA_Panama_W
L_bolivianus_KRL_1114_PA_Cocle_W
L_bolivianus_CH_4956_PA_Panama_W
L_bolivianus_AJC_3235_VE_Amacuro_E
L_bolivianus_AJC_3298_VE_Amacuro_E
L_bolivianus_MHNLS_19742_VE_Bolivar_E
L_bolivianus_MHNLS_19743_VE_Bolivar_E
L_bolivianus_MHNLS_19715_VE_Bolivar_E
L_bolivianus_MHNLS_17786_VE_Bolivar_E
L_bolivianus_CORBIDI5179_PE_E
L_bolivianus_SCF_145_PE_Ucayali_E
L_bolivianus_CORBIDI_5182_PE_E
L_bolivianus_SCF_147_PE_Ucayali_E
L_bolivianus_SCF_146_PE_Ucayali_E
L_chaquensis_CFBHT11586_OG
L_vastus_CFBHT13593_OG
L_fuscus_AJC_2301_OG
L_rhodomystax_MTR13979_OG
Lithodytes_lineatus_AJC_3451_OG
1
1
1
1
1
1
0.89
1
30
25
20
15
10
5
0
Million years before present (Ma)

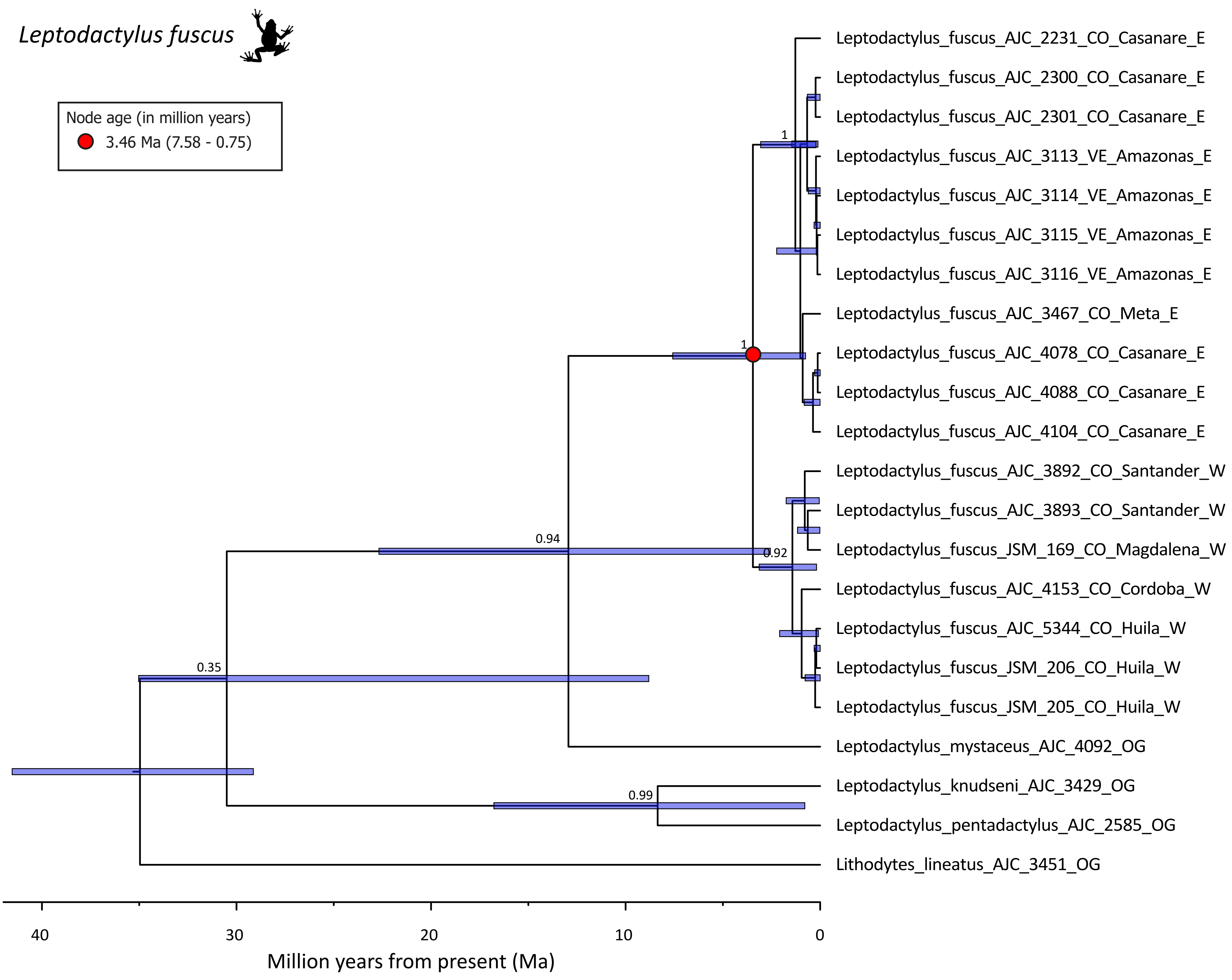
Leptodactylus fuscus
Node age (in million years)
3.46 Ma (7.58 - 0.75)
Leptodactylus_fuscus_AJC_2231_CO_Casanare_E
Leptodactylus_fuscus_AJC_2300_CO_Casanare_E
Leptodactylus_fuscus_AJC_2301_CO_Casanare_E
Leptodactylus_fuscus_AJC_3113_VE_Amazonas_E
Leptodactylus_fuscus_AJC_3114_VE_Amazonas_E
Leptodactylus_fuscus_AJC_3115_VE_Amazonas_E
Leptodactylus_fuscus_AJC_3116_VE_Amazonas_E
Leptodactylus_fuscus_AJC_3467_CO_Meta_E
Leptodactylus_fuscus_AJC_4078_CO_Casanare_E
Leptodactylus_fuscus_AJC_4088_CO_Casanare_E
Leptodactylus_fuscus_AJC_4104_CO_Casanare_E
Leptodactylus_fuscus_AJC_3892_CO_Santander_W
Leptodactylus_fuscus_AJC_3893_CO_Santander_W
Leptodactylus_fuscus_JSM_169_CO_Magdalena_W
Leptodactylus_fuscus_AJC_4153_CO_Cordoba_W
Leptodactylus_fuscus_AJC_5344_CO_Huila_W
Leptodactylus_fuscus_JSM_206_CO_Huila_W
Leptodactylus_fuscus_JSM_205_CO_Huila_W
Leptodactylus_mystaceus_AJC_4092_OG
Leptodactylus_knudseni_AJC_3429_OG
Leptodactylus_pentadactylus_AJC_2585_OG
Lithodytes_lineatus_AJC_3451_OG
1
1
0.94
0.92
0.35
0.99
40
30
20
10
0
Million years from present (Ma)

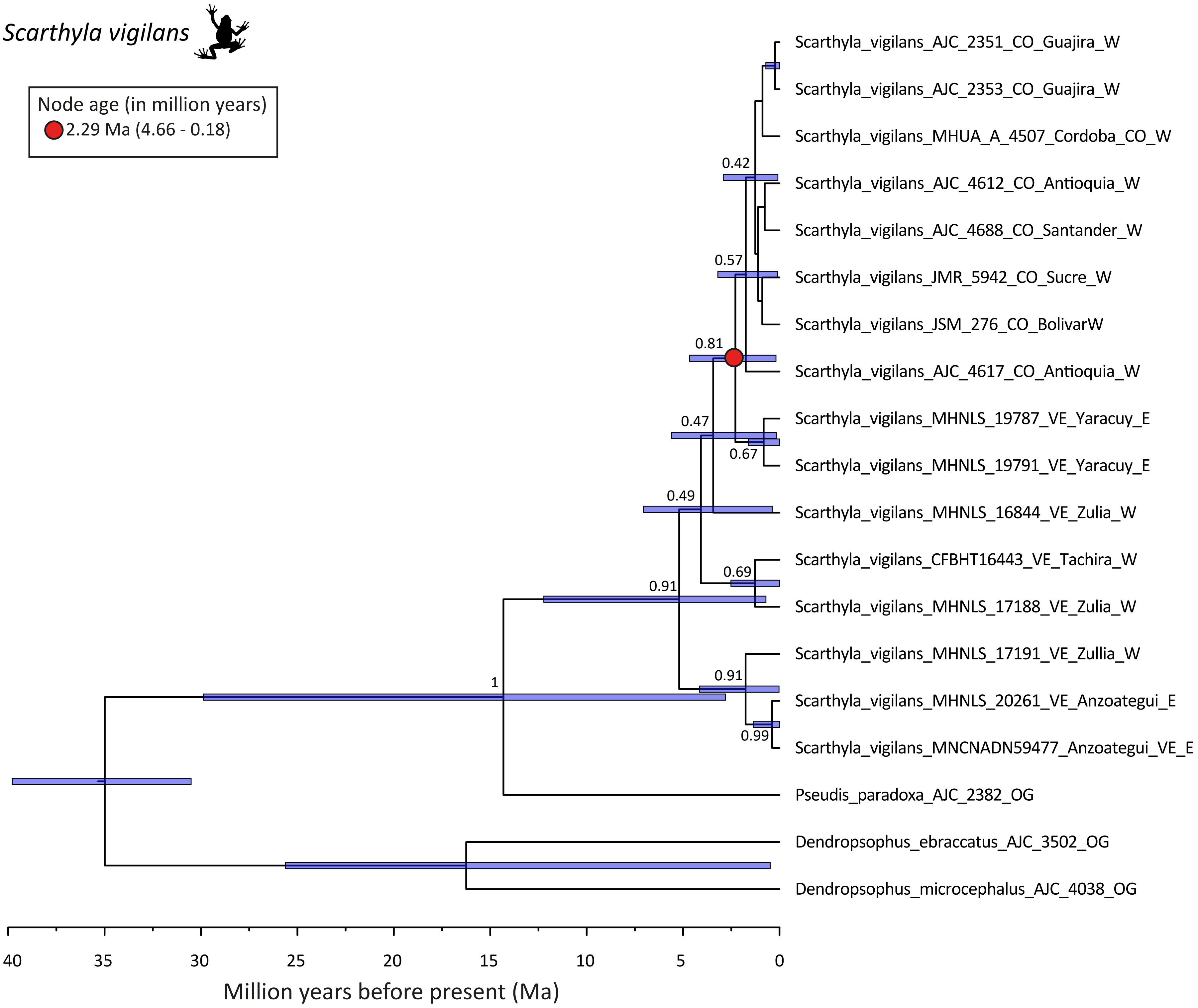

Scarthyla vigilans
Node age (in million years)
2.29 Ma (4.66 - 0.18)
Scarthyla_vigilans_AJC_2351_CO_Guajira_W
Scarthyla_vigilans_AJC_2353_CO_Guajira_W
Scarthyla_vigilans_MHUA_A_4507_Cordoba_CO_W
Scarthyla_vigilans_AJC_4612_CO_Antioquia_W
Scarthyla_vigilans_AJC_4688_CO_Santander_W
Scarthyla_vigilans_JMR_5942_CO_Sucre_W
Scarthyla_vigilans_JSM_276_CO_BolivarW
Scarthyla_vigilans_AJC_4617_CO_Antioquia_W
Scarthyla_vigilans_MHNLS_19787_VE_Yaracuy_E
Scarthyla_vigilans_MHNLS_19791_VE_Yaracuy_E
Scarthyla_vigilans_MHNLS_16844_VE_Zulia_W
Scarthyla_vigilans_CFBHT16443_VE_Tachira_W
Scarthyla_vigilans_MHNLS_17188_VE_Zulia_W
Scarthyla_vigilans_MHNLS_17191_VE_Zullia_W
Scarthyla_vigilans_MHNLS_20261_VE_Anzoategui_E
Scarthyla_vigilans_MNCNADN59477_Anzoategui_VE_E
Pseudis_paradoxa_AJC_2382_OG
Dendropsophus_ebraccatus_AJC_3502_OG
Dendropsophus_microcephalus_AJC_4038_OG
0.42
0.57
0.81
0.47
0.67
0.49
0.69
0.91
1
0.91
0.99
40
35
30
25
20
15
10
5
0
Million years before present (Ma)

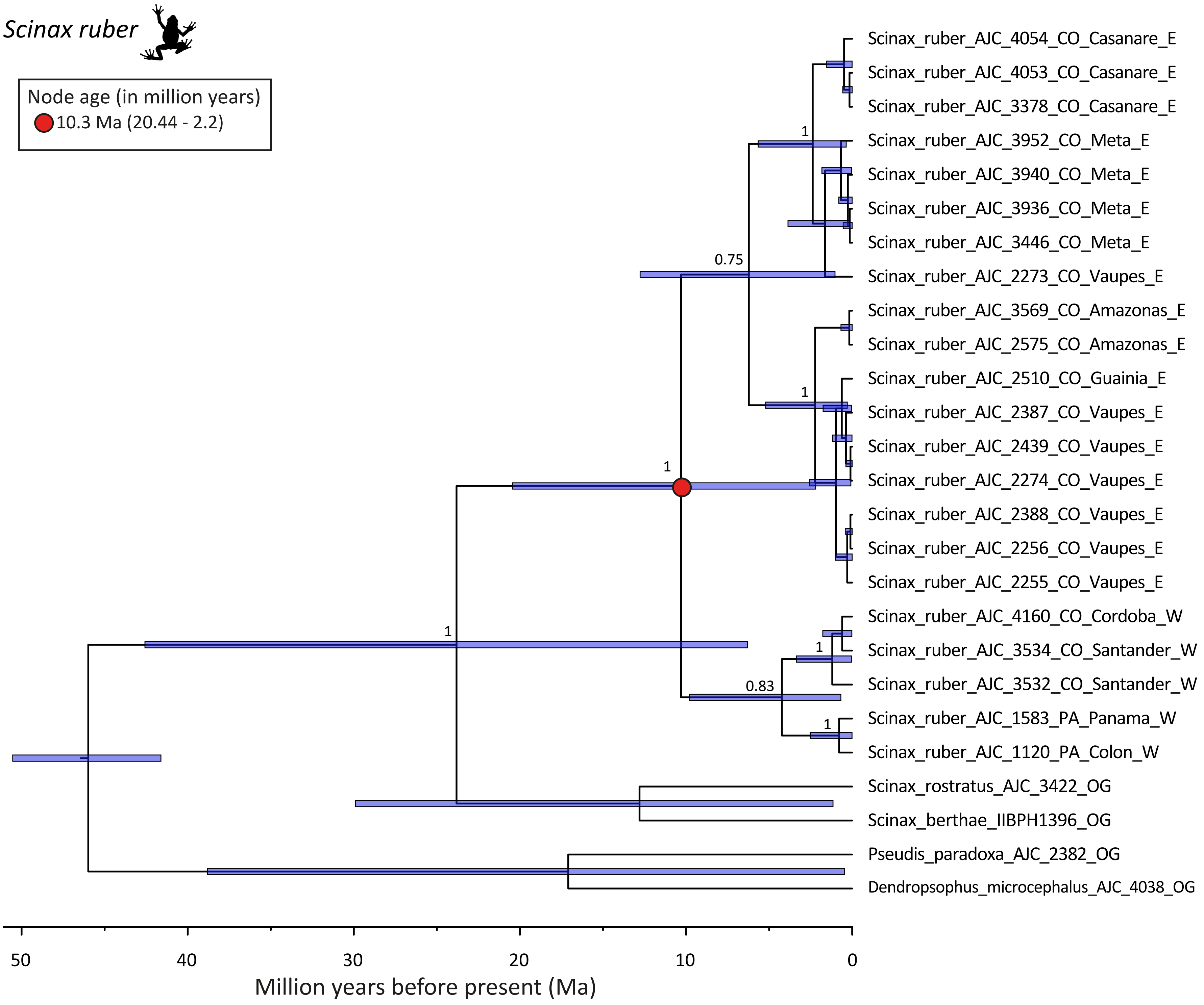

Scinax ruber
Node age (in million years)
10.3 Ma (20.44 - 2.2)
Scinax_ruber_AJC_4054_CO_Casanare_E
Scinax_ruber_AJC_4053_CO_Casanare_E
Scinax_ruber_AJC_3378_CO_Casanare_E
Scinax_ruber_AJC_3952_CO_Meta_E
Scinax_ruber_AJC_3940_CO_Meta_E
Scinax_ruber_AJC_3936_CO_Meta_E
Scinax_ruber_AJC_3446_CO_Meta_E
Scinax_ruber_AJC_2273_CO_Vaupes_E
Scinax_ruber_AJC_3569_CO_Amazonas_E
Scinax_ruber_AJC_2575_CO_Amazonas_E
Scinax_ruber_AJC_2510_CO_Guainia_E
Scinax_ruber_AJC_2387_CO_Vaupes_E
Scinax_ruber_AJC_2439_CO_Vaupes_E
Scinax_ruber_AJC_2274_CO_Vaupes_E
Scinax_ruber_AJC_2388_CO_Vaupes_E
Scinax_ruber_AJC_2256_CO_Vaupes_E
Scinax_ruber_AJC_2255_CO_Vaupes_E
Scinax_ruber_AJC_4160_CO_Cordoba_W
Scinax_ruber_AJC_3534_CO_Santander_W
Scinax_ruber_AJC_3532_CO_Santander_W
Scinax_ruber_AJC_1583_PA_Panama_W
Scinax_ruber_AJC_1120_PA_Colon_W
Scinax_rostratus_AJC_3422_OG
Scinax_berthae_IIBPH1396_OG
Pseudis_paradoxa_AJC_2382_OG
Dendropsophus_microcephalus_AJC_4038_OG
1
0.75
1
1
1
1
0.83
1
50
40
30
20
10
0
Million years before present (Ma)

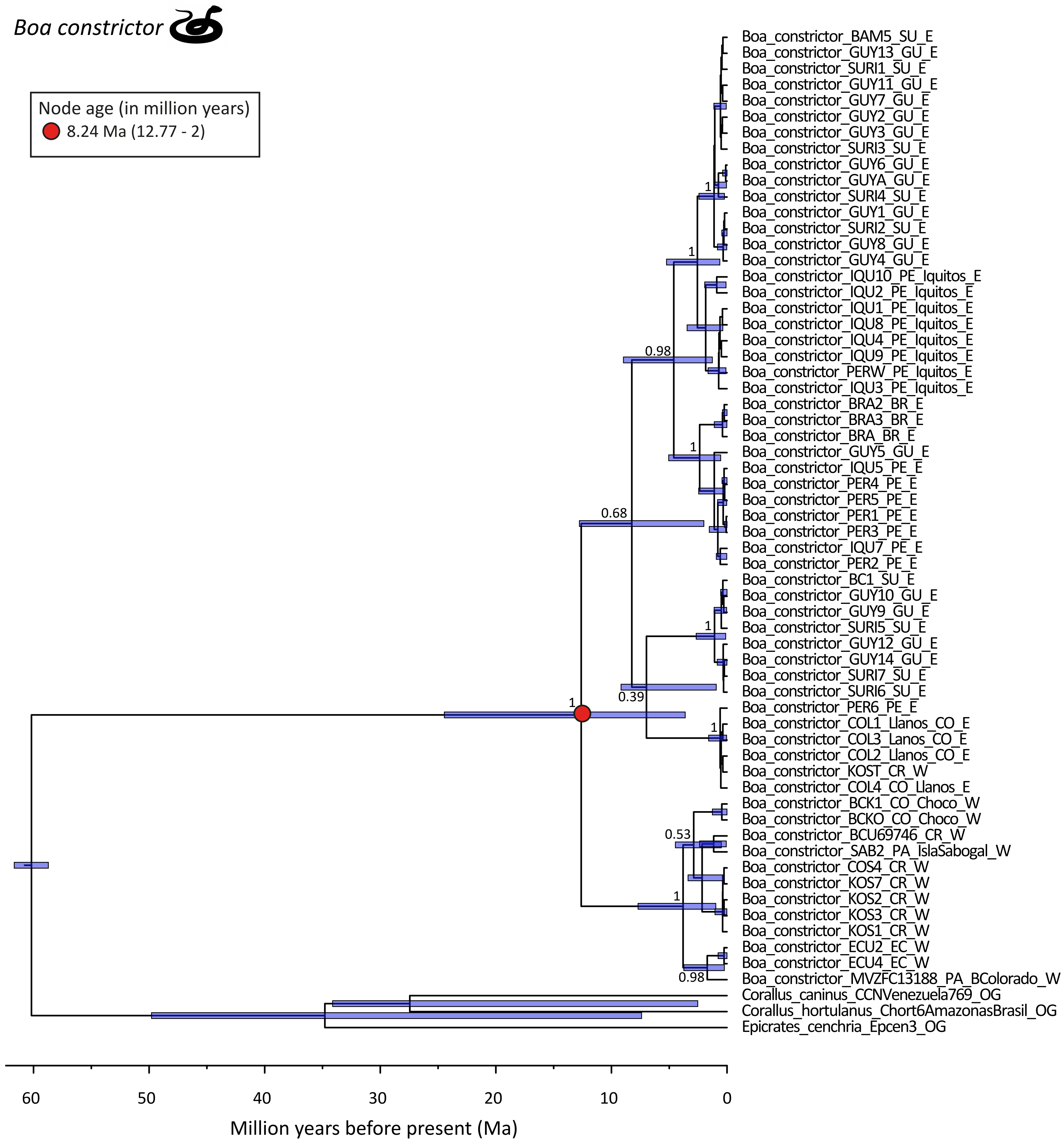

Boa constrictor
Node age (in million years)
8.24 Ma (12.77 - 2)
Boa_constrictor_BAM5_SU_E
Boa_constrictor_GUY13_GU_E
Boa_constrictor_SURI1_SU_E
Boa_constrictor_GUY11_GU_E
Boa_constrictor_GUY7_GU_E
Boa_constrictor_GUY2_GU_E
Boa_constrictor_GUY3_GU_E
Boa_constrictor_SURI3_SU_E
Boa_constrictor_GUY6_GU_E
Boa_constrictor_GUYA_GU_E
Boa_constrictor_SURI4_SU_E
Boa_constrictor_GUY1_GU_E
Boa_constrictor_SURI2_SU_E
Boa_constrictor_GUY8_GU_E
Boa_constrictor_GUY4_GU_E
Boa_constrictor_IQU10_PE_Iquitos_E
Boa_constrictor_IQU2_PE_Iquitos_E
Boa_constrictor_IQU1_PE_Iquitos_E
Boa_constrictor_IQU8_PE_Iquitos_E
Boa_constrictor_IQU4_PE_Iquitos_E
Boa_constrictor_IQU9_PE_Iquitos_E
Boa_constrictor_PERW_PE_Iquitos_E
Boa_constrictor_IQU3_PE_Iquitos_E
Boa_constrictor_BRA2_BR_E
Boa_constrictor_BRA3_BR_E
Boa_constrictor_BRA_BR_E
Boa_constrictor_GUY5_GU_E
Boa_constrictor_IQU5_PE_E
Boa_constrictor_PER4_PE_E
Boa_constrictor_PER5_PE_E
Boa_constrictor_PER1_PE_E
Boa_constrictor_PER3_PE_E
Boa_constrictor_IQU7_PE_E
Boa_constrictor_PER2_PE_E
Boa_constrictor_BC1_SU_E
Boa_constrictor_GUY10_GU_E
Boa_constrictor_GUY9_GU_E
Boa_constrictor_SURI5_SU_E
Boa_constrictor_GUY12_GU_E
Boa_constrictor_GUY14_GU_E
Boa_constrictor_SURI7_SU_E
Boa_constrictor_SURI6_SU_E
Boa_constrictor_PER6_PE_E
Boa_constrictor_COL1_Llanos_CO_E
Boa_constrictor_COL3_Lanos_CO_E
Boa_constrictor_COL2_Llanos_CO_E
Boa_constrictor_KOST_CR_W
Boa_constrictor_COL4_CO_Llanos_E
Boa_constrictor_BCK1_CO_Choco_W
Boa_constrictor_BCKO_CO_Choco_W
Boa_constrictor_BCU69746_CR_W
Boa_constrictor_SAB2_PA_IslaSabogal_W
Boa_constrictor_COS4_CR_W
Boa_constrictor_KOS7_CR_W
Boa_constrictor_KOS2_CR_W
Boa_constrictor_KOS3_CR_W
Boa_constrictor_KOS1_CR_W
Boa_constrictor_ECU2_EC_W
Boa_constrictor_ECU4_EC_W
Boa_constrictor_MVZFC13188_PA_BColorado_W
Corallus_caninus_CCNVenezuela769_OG
Corallus_hortulanus_Chort6AmazonasBrasil_OG
Epicrates_cenchria_Epcen3_OG
1
1
0.98
1
0.68
1
1
0.39
1
0.53
1
0.98
60
50
40
30
20
10
0
Million years before present (Ma)

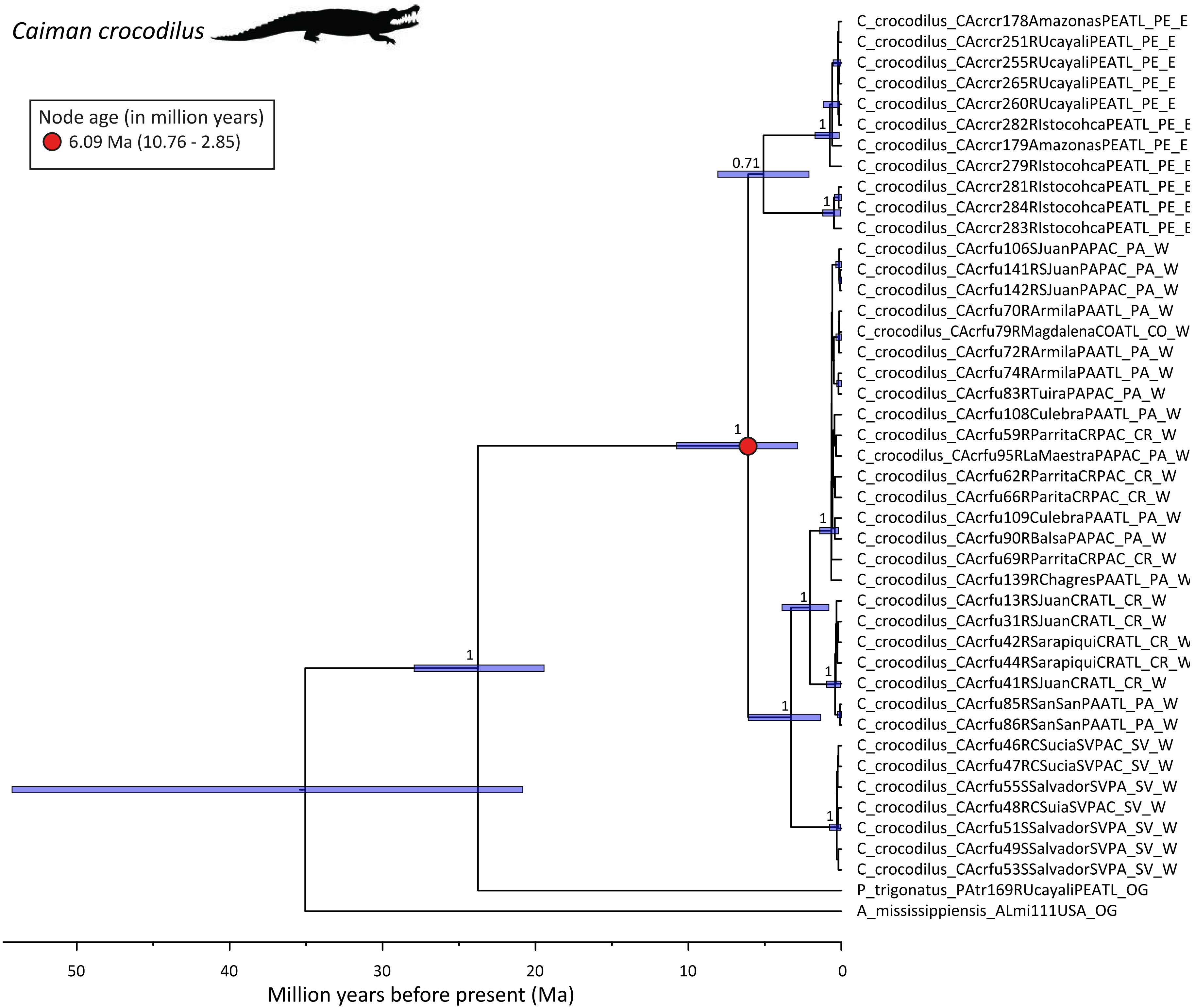

Caiman crocodilus
Node age (in million years)
6.09 Ma (10.76 - 2.85)
C_crocodilus_CAcrcr178AmazonasPEATL_PE_E
C_crocodilus_CAcrcr251RUcayaliPEATL_PE_E
C_crocodilus_CAcrcr255RUcayaliPEATL_PE_E
C_crocodilus_CAcrcr265RUcayaliPEATL_PE_E
C_crocodilus_CAcrcr260RUcayaliPEATL_PE_E
C_crocodilus_CAcrcr282RIstocohcaPEATL_PE_E
C_crocodilus_CAcrcr179AmazonasPEATL_PE_E
C_crocodilus_CAcrcr279RIstocohcaPEATL_PE_E
C_crocodilus_CAcrcr281RIstocohcaPEATL_PE_E
C_crocodilus_CAcrcr284RIstocohcaPEATL_PE_E
C_crocodilus_CAcrcr283RIstocohcaPEATL_PE_E
C_crocodilus_CAcrfu106SJuanPAPAC_PA_W
C_crocodilus_CAcrfu141RSJuanPAPAC_PA_W
C_crocodilus_CAcrfu142RSJuanPAPAC_PA_W
C_crocodilus_CAcrfu70RArmilaPAATL_PA_W
C_crocodilus_CAcrfu79RMagdalenaCOATL_CO_W
C_crocodilus_CAcrfu72RArmilaPAATL_PA_W
C_crocodilus_CAcrfu74RArmilaPAATL_PA_W
C_crocodilus_CAcrfu83RTuiraPAPAC_PA_W
C_crocodilus_CAcrfu108CulebraPAATL_PA_W
C_crocodilus_CAcrfu59RParritaCRPAC_CR_W
C_crocodilus_CAcrfu95RLaMaestraPAPAC_PA_W
C_crocodilus_CAcrfu62RParritaCRPAC_CR_W
C_crocodilus_CAcrfu66RParitaCRPAC_CR_W
C_crocodilus_CAcrfu109CulebraPAATL_PA_W
C_crocodilus_CAcrfu90RBalsaPAPAC_PA_W
C_crocodilus_CAcrfu69RParritaCRPAC_CR_W
C_crocodilus_CAcrfu139RChagresPAATL_PA_W
C_crocodilus_CAcrfu13RSJuanCRATL_CR_W
C_crocodilus_CAcrfu31RSJuanCRATL_CR_W
C_crocodilus_CAcrfu42RSarapiquiCRATL_CR_W
C_crocodilus_CAcrfu44RSarapiquiCRATL_CR_W
C_crocodilus_CAcrfu41RSJuanCRATL_CR_W
C_crocodilus_CAcrfu85RSanSanPAATL_PA_W
C_crocodilus_CAcrfu86RSanSanPAATL_PA_W
C_crocodilus_CAcrfu46RCSuciaSVPAC_SV_W
C_crocodilus_CAcrfu47RCSuciaSVPAC_SV_W
C_crocodilus_CAcrfu55SSalvadorSVPA_SV_W
C_crocodilus_CAcrfu48RCSuiaSVPAC_SV_W
C_crocodilus_CAcrfu51SSalvadorSVPA_SV_W
C_crocodilus_CAcrfu49SSalvadorSVPA_SV_W
C_crocodilus_CAcrfu53SSalvadorSVPA_SV_W
P_trigonatus_PAtr169RUcayaliPEATL_OG
A_mississippiensis_ALmi111USA_OG
1
0.71
1
1
1
1
1
1
1
1
50
40
30
20
10
0
Million years before present (Ma)

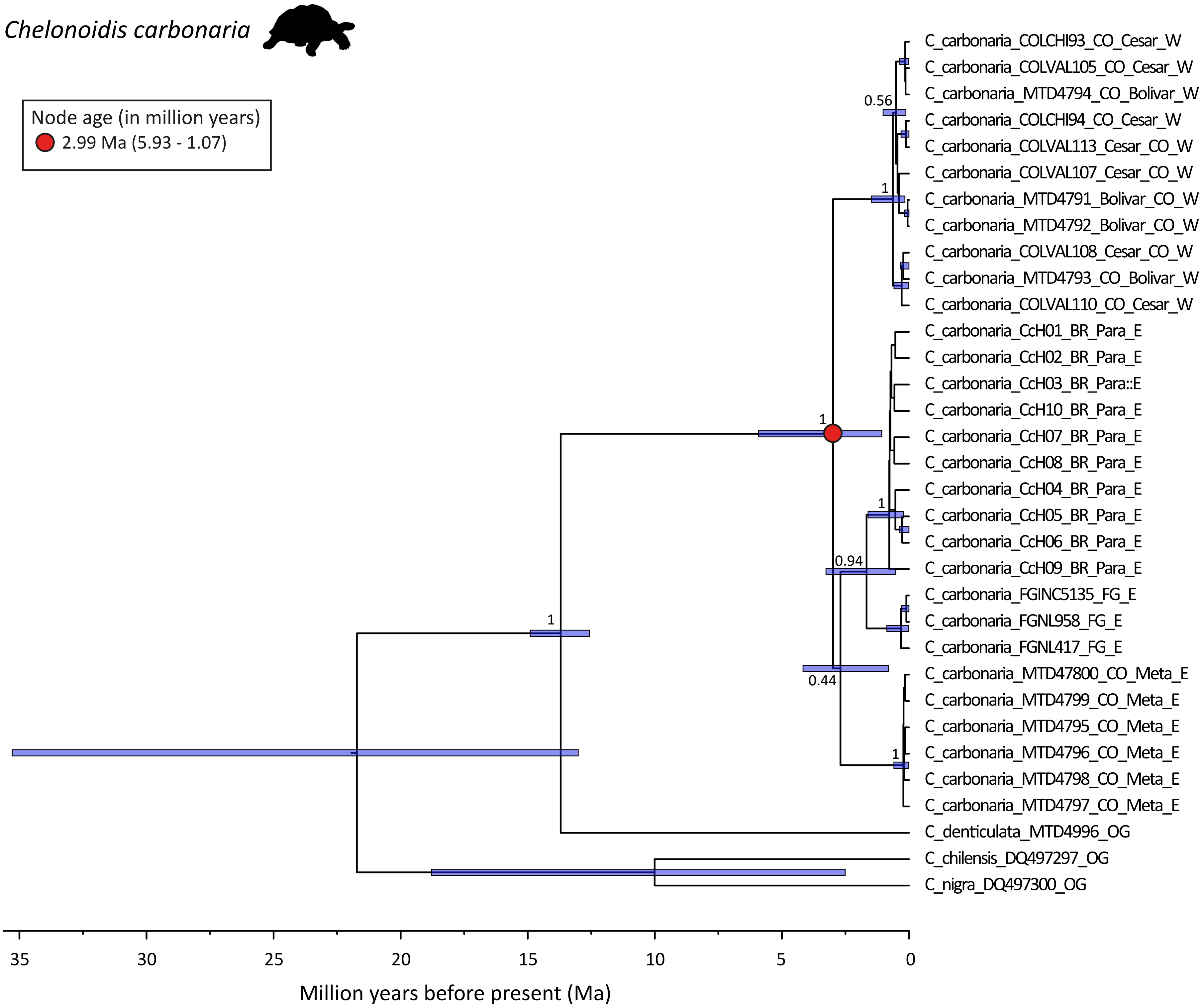

Chelonoidis carbonaria
Node age (in million years)
2.99 Ma (5.93 - 1.07)
C_carbonaria_COLCHI93_CO_Cesar_W
C_carbonaria_COLVAL105_CO_Cesar_W
C_carbonaria_MTD4794_CO_Bolivar_W
C_carbonaria_COLCHI94_CO_Cesar_W
C_carbonaria_COLVAL113_Cesar_CO_W
C_carbonaria_COLVAL107_Cesar_CO_W
C_carbonaria_MTD4791_Bolivar_CO_W
C_carbonaria_MTD4792_Bolivar_CO_W
C_carbonaria_COLVAL108_Cesar_CO_W
C_carbonaria_MTD4793_CO_Bolivar_W
C_carbonaria_COLVAL110_CO_Cesar_W
C_carbonaria_CcH01_BR_Para_E
C_carbonaria_CcH02_BR_Para_E
C_carbonaria_CcH03_BR_Para::E
C_carbonaria_CcH10_BR_Para_E
C_carbonaria_CcH07_BR_Para_E
C_carbonaria_CcH08_BR_Para_E
C_carbonaria_CcH04_BR_Para_E
C_carbonaria_CcH05_BR_Para_E
C_carbonaria_CcH06_BR_Para_E
C_carbonaria_CcH09_BR_Para_E
C_carbonaria_FGINC5135_FG_E
C_carbonaria_FGNL958_FG_E
C_carbonaria_FGNL417_FG_E
C_carbonaria_MTD47800_CO_Meta_E
C_carbonaria_MTD4799_CO_Meta_E
C_carbonaria_MTD4795_CO_Meta_E
C_carbonaria_MTD4796_CO_Meta_E
C_carbonaria_MTD4798_CO_Meta_E
C_carbonaria_MTD4797_CO_Meta_E
C_denticulata_MTD4996_OG
C_chilensis_DQ497297_OG
C_nigra_DQ497300_OG
0.56
1
1
1
0.94
0.44
1
1
35
30
25
20
15
10
5
0
Million years before present (Ma)

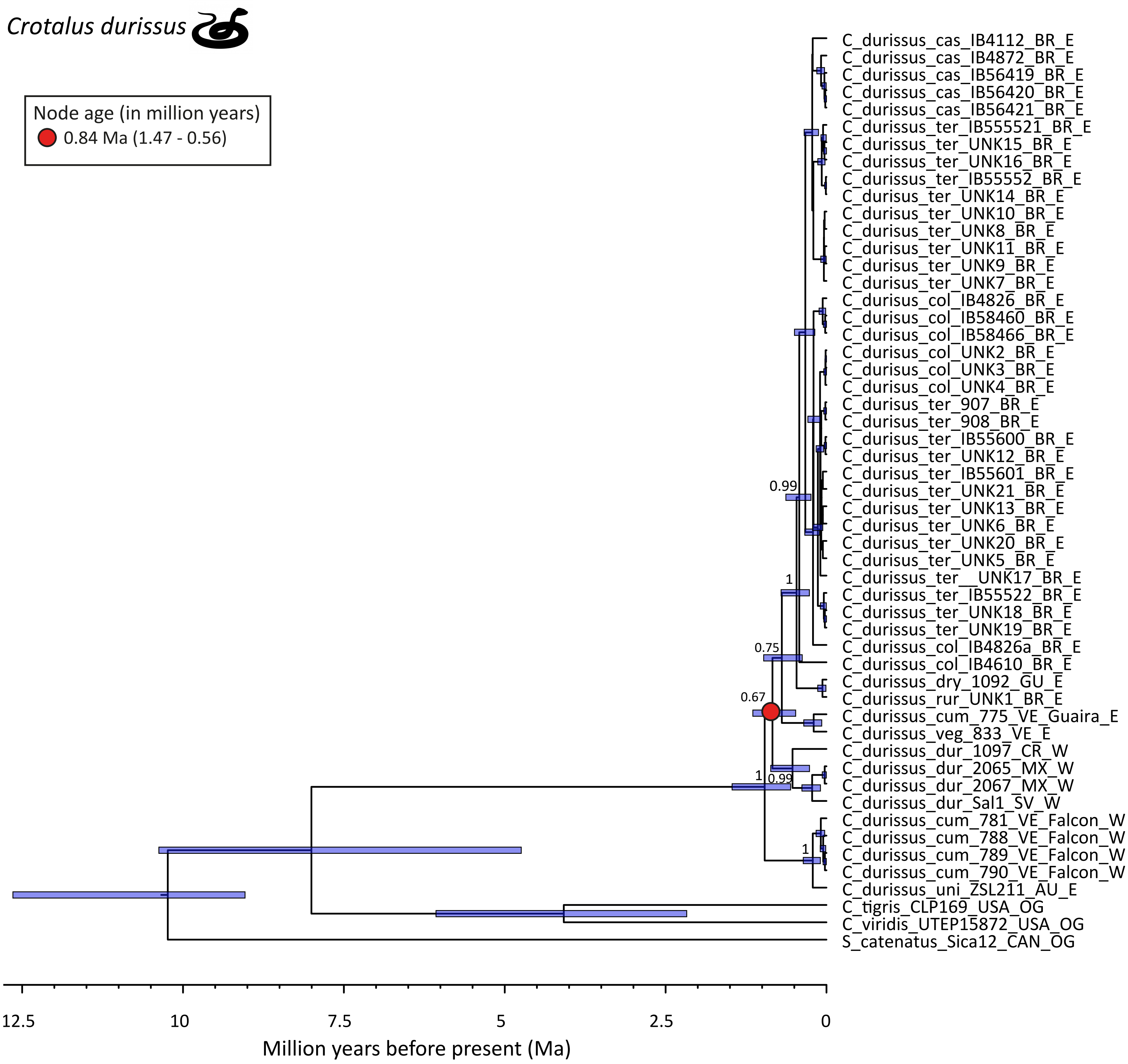

Crotalus durissus
Node age (in million years)
0.84 Ma (1.47 - 0.56)
C_durissus_cas_IB4112_BR_E
C_durissus_cas_IB4872_BR_E
C_durissus_cas_IB56419_BR_E
C_durissus_cas_IB56420_BR_E
C_durissus_cas_IB56421_BR_E
C_durissus_ter_IB555521_BR_E
C_durissus_ter_UNK15_BR_E
C_durissus_ter_UNK16_BR_E
C_durissus_ter_IB55552_BR_E
C_durisus_ter_UNK14_BR_E
C_durisus_ter_UNK10_BR_E
C_durisus_ter_UNK8_BR_E
C_durisus_ter_UNK11_BR_E
C_durisus_ter_UNK9_BR_E
C_durisus_ter_UNK7_BR_E
C_durisus_col_IB4826_BR_E
C_durisus_col_IB58460_BR_E
C_durisus_col_IB58466_BR_E
C_durisus_col_UNK2_BR_E
C_durisus_col_UNK3_BR_E
C_durisus_col_UNK4_BR_E
C_durisus_ter_907_BR_E
C_durisus_ter_908_BR_E
C_durisus_ter_IB55600_BR_E
C_durisus_ter_UNK12_BR_E
C_durisus_ter_IB55601_BR_E
C_durisus_ter_UNK21_BR_E
C_durisus_ter_UNK13_BR_E
C_durisus_ter_UNK6_BR_E
C_durisus_ter_UNK20_BR_E
C_durisus_ter_UNK5_BR_E
C_durissus_ter__UNK17_BR_E
C_durissus_ter_IB55522_BR_E
C_durissus_ter_UNK18_BR_E
C_durissus_ter_UNK19_BR_E
C_durissus_col_IB4826a_BR_E
C_durissus_col_IB4610_BR_E
C_durissus_dry_1092_GU_E
C_durissus_rur_UNK1_BR_E
C_durissus_cum_775_VE_Guaira_E
C_durissus_veg_833_VE_E
C_durissus_dur_1097_CR_W
C_durissus_dur_2065_MX_W
C_durissus_dur_2067_MX_W
C_durissus_dur_Sal1_SV_W
C_durissus_cum_781_VE_Falcon_W
C_durissus_cum_788_VE_Falcon_W
C_durissus_cum_789_VE_Falcon_W
C_durissus_cum_790_VE_Falcon_W
C_durissus_uni_ZSL211_AU_E
C_tigris_CLP169_USA_OG
C_viridis_UTEP15872_USA_OG
S_catenatus_Sica12_CAN_OG
0.99
1
0.75
0.67
1
0.99
1
12.5
10
7.5
5
2.5
0
Million years before present (Ma)

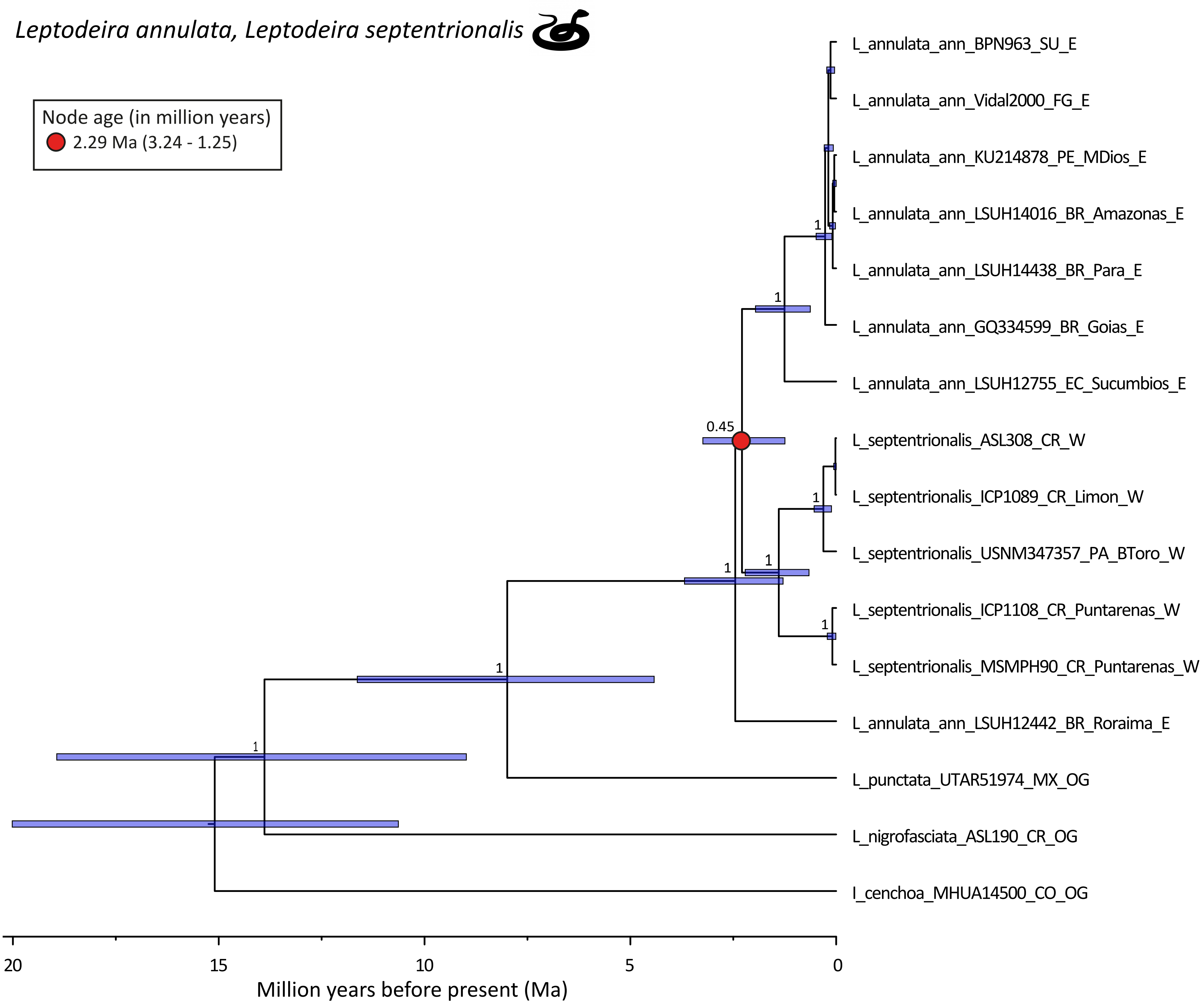

Leptodeira annulata, Leptodeira septentrionalis
Node age (in million years)
2.29 Ma (3.24 - 1.25)
L_annulata_ann_BPN963_SU_E
L_annulata_ann_Vidal2000_FG_E
L_annulata_ann_KU214878_PE_MDios_E
L_annulata_ann_LSUH14016_BR_Amazonas_E
L_annulata_ann_LSUH14438_BR_Para_E
L_annulata_ann_GQ334599_BR_Goias_E
L_annulata_ann_LSUH12755_EC_Sucumbios_E
L_septentrionalis_ASL308_CR_W
L_septentrionalis_ICP1089_CR_Limon_W
L_septentrionalis_USNM347357_PA_BToro_W
L_septentrionalis_ICP1108_CR_Puntarenas_W
L_septentrionalis_MSMPH90_CR_Puntarenas_W
L_annulata_ann_LSUH12442_BR_Roraima_E
L_punctata_UTAR51974_MX_OG
L_nigrofasciata_ASL190_CR_OG
I_cenchoa_MHUA14500_CO_OG
0.45
1
20
15
10
5
0
Million years before present (Ma)

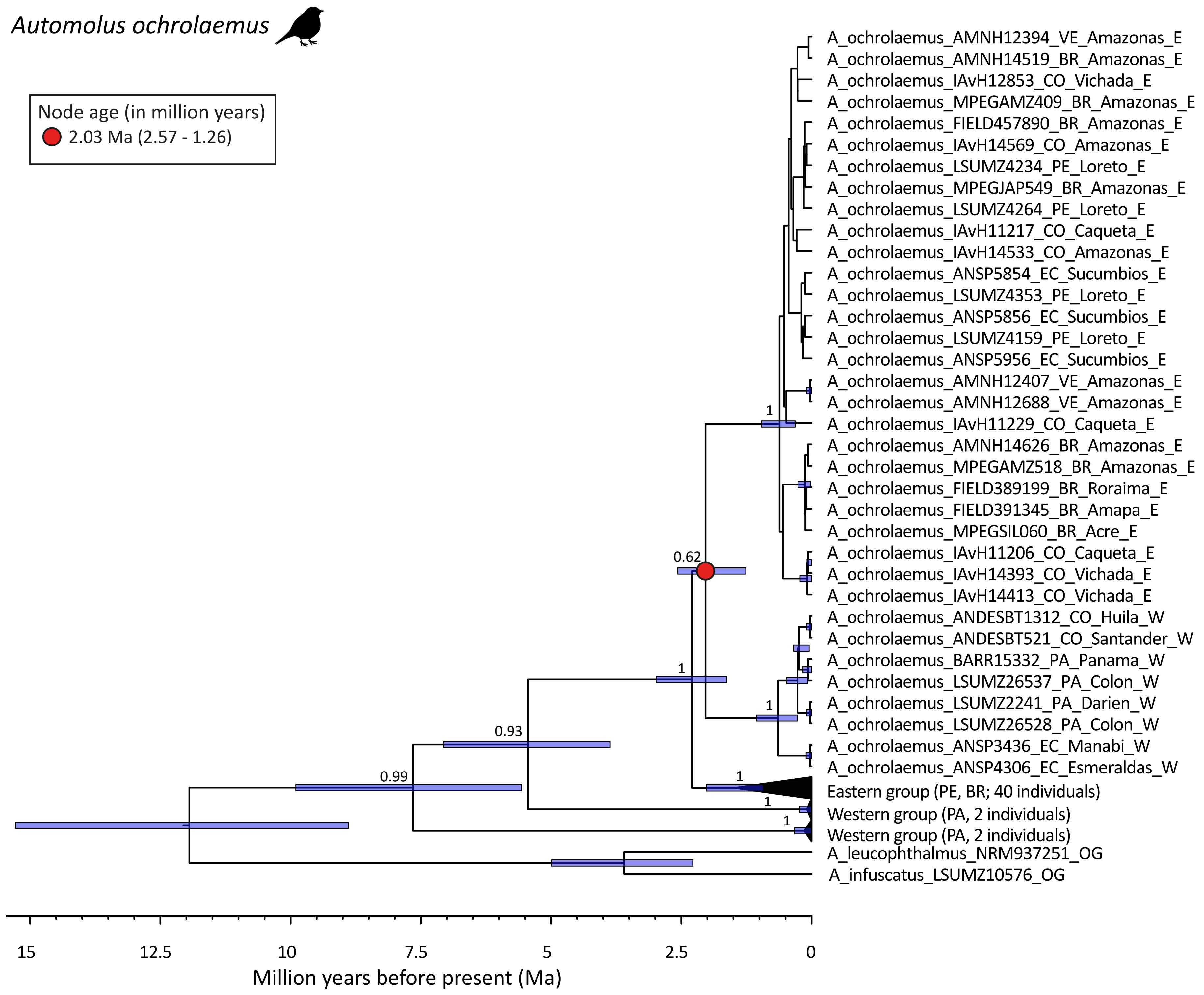

Automolus ochrolaemus
Node age (in million years)
2.03 Ma (2.57 - 1.26)
A_ochrolaemus_AMNH12394_VE_Amazonas_E
A_ochrolaemus_AMNH14519_BR_Amazonas_E
A_ochrolaemus_IAvH12853_CO_Vichada_E
A_ochrolaemus_MPEGAMZ409_BR_Amazonas_E
A_ochrolaemus_FIELD457890_BR_Amazonas_E
A_ochrolaemus_IAvH14569_CO_Amazonas_E
A_ochrolaemus_LSUMZ4234_PE_Loreto_E
A_ochrolaemus_MPEGJAP549_BR_Amazonas_E
A_ochrolaemus_LSUMZ4264_PE_Loreto_E
A_ochrolaemus_IAvH11217_CO_Caqueta_E
A_ochrolaemus_IAvH14533_CO_Amazonas_E
A_ochrolaemus_ANSP5854_EC_Sucumbios_E
A_ochrolaemus_LSUMZ4353_PE_Loreto_E
A_ochrolaemus_ANSP5856_EC_Sucumbios_E
A_ochrolaemus_LSUMZ4159_PE_Loreto_E
A_ochrolaemus_ANSP5956_EC_Sucumbios_E
A_ochrolaemus_AMNH12407_VE_Amazonas_E
A_ochrolaemus_AMNH12688_VE_Amazonas_E
A_ochrolaemus_IAvH11229_CO_Caqueta_E
A_ochrolaemus_AMNH14626_BR_Amazonas_E
A_ochrolaemus_MPEGAMZ518_BR_Amazonas_E
A_ochrolaemus_FIELD389199_BR_Roraima_E
A_ochrolaemus_FIELD391345_BR_Amapa_E
A_ochrolaemus_MPEGSIL060_BR_Acre_E
A_ochrolaemus_IAvH11206_CO_Caqueta_E
A_ochrolaemus_IAvH14393_CO_Vichada_E
A_ochrolaemus_IAvH14413_CO_Vichada_E
A_ochrolaemus_ANDESBT1312_CO_Huila_W
A_ochrolaemus_ANDESBT521_CO_Santander_W
A_ochrolaemus_BARR15332_PA_Panama_W
A_ochrolaemus_LSUMZ26537_PA_Colon_W
A_ochrolaemus_LSUMZ2241_PA_Darien_W
A_ochrolaemus_LSUMZ26528_PA_Colon_W
A_ochrolaemus_ANSP3436_EC_Manabi_W
A_ochrolaemus_ANSP4306_EC_Esmeraldas_W
Eastern group (PE, BR; 40 individuals)
Western group (PA, 2 individuals)
Western group (PA, 2 individuals)
A_leucophthalmus_NRM937251_OG
A_infuscatus_LSUMZ10576_OG
1
0.62
1
1
0.93
0.99
1
1
1
1
15
12.5
10
7.5
5
2.5
0
Million years before present (Ma)

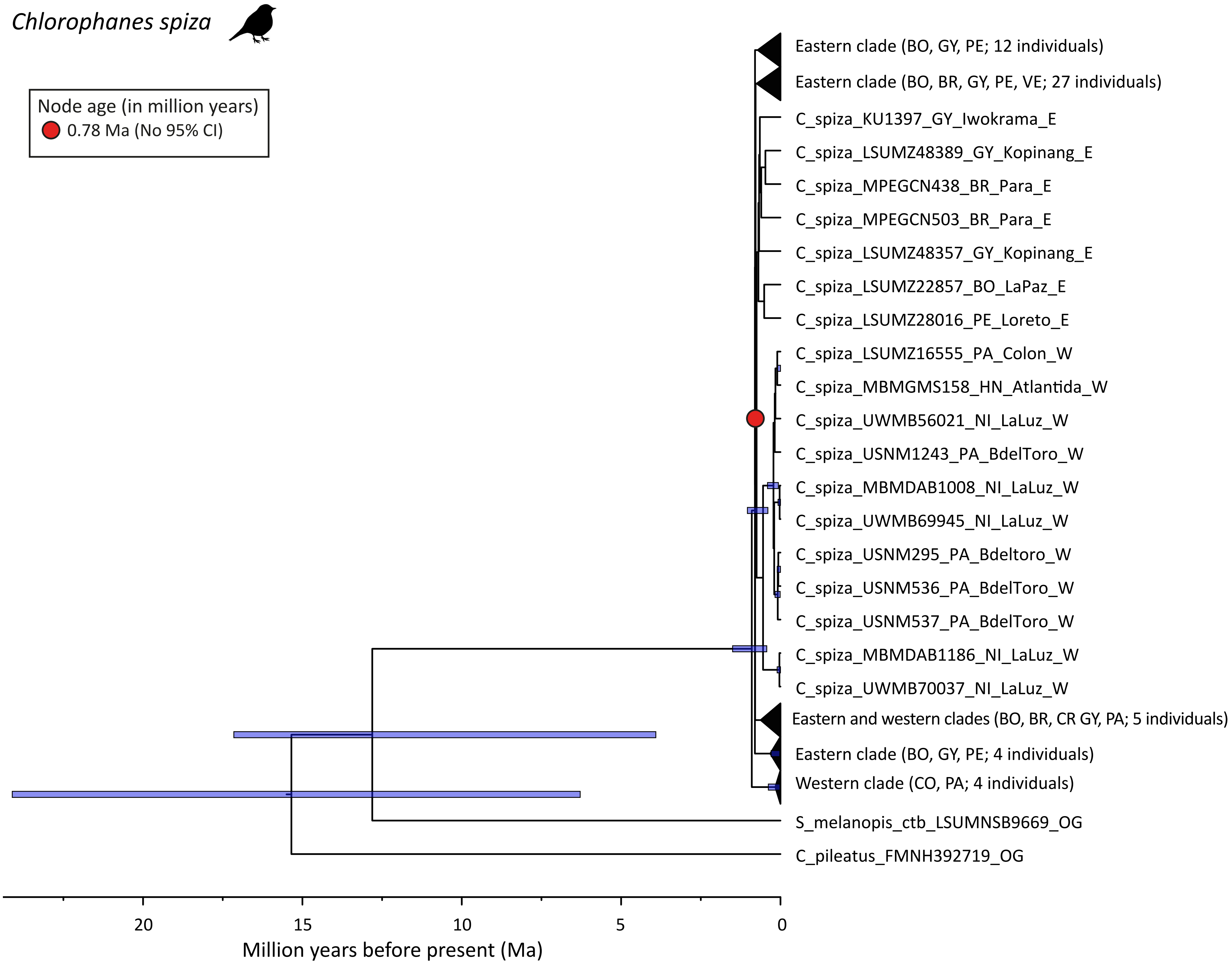
Chlorophanes spiza
Node age (in million years)
0.78 Ma (No 95% CI)
Eastern clade (BO, GY, PE; 12 individuals)
Eastern clade (BO, BR, GY, PE, VE; 27 individuals)
C_spiza_KU1397_GY_Iwokrama_E
C_spiza_LSUMZ48389_GY_Kopinang_E
C_spiza_MPEGCN438_BR_Para_E
C_spiza_MPEGCN503_BR_Para_E
C_spiza_LSUMZ48357_GY_Kopinang_E
C_spiza_LSUMZ22857_BO_LaPaz_E
C_spiza_LSUMZ28016_PE_Loreto_E
C_spiza_LSUMZ16555_PA_Colon_W
C_spiza_MBMGMS158_HN_Atlantida_W
C_spiza_UWMB56021_NI_LaLuz_W
C_spiza_USNM1243_PA_BdelToro_W
C_spiza_MBMDAB1008_NI_LaLuz_W
C_spiza_UWMB69945_NI_LaLuz_W
C_spiza_USNM295_PA_Bdeltoro_W
C_spiza_USNM536_PA_BdelToro_W
C_spiza_USNM537_PA_BdelToro_W
C_spiza_MBMDAB1186_NI_LaLuz_W
C_spiza_UWMB70037_NI_LaLuz_W
Eastern and western clades (BO, BR, CR GY, PA; 5 individuals)
Eastern clade (BO, GY, PE; 4 individuals)
Western clade (CO, PA; 4 individuals)
S_melanopis_ctb_LSUMNSB9669_OG
C_pileatus_FMNH392719_OG
20
15
10
5
0
Million years before present (Ma)

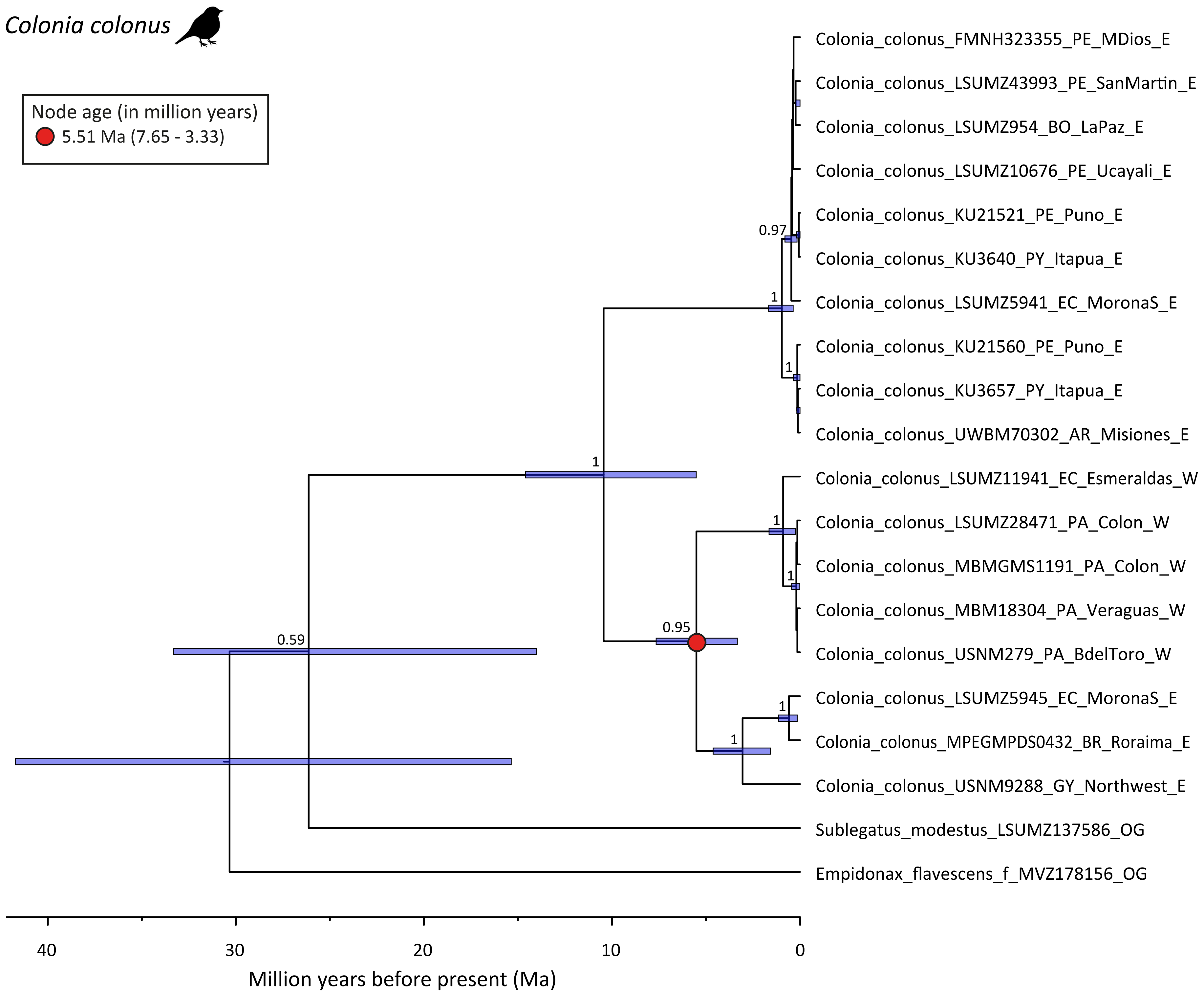
Colonia colonus
Node age (in million years)
5.51 Ma (7.65 - 3.33)
Colonia_colonus_FMNH323355_PE_MDios_E
Colonia_colonus_LSUMZ43993_PE_SanMartin_E
Colonia_colonus_LSUMZ954_BO_LaPaz_E
Colonia_colonus_LSUMZ10676_PE_Ucayali_E
Colonia_colonus_KU21521_PE_Puno_E
Colonia_colonus_KU3640_PY_Itapua_E
Colonia_colonus_LSUMZ5941_EC_MoronaS_E
Colonia_colonus_KU21560_PE_Puno_E
Colonia_colonus_KU3657_PY_Itapua_E
Colonia_colonus_UWBM70302_AR_Misiones_E
Colonia_colonus_LSUMZ11941_EC_Esmeraldas_W
Colonia_colonus_LSUMZ28471_PA_Colon_W
Colonia_colonus_MBMGMS1191_PA_Colon_W
Colonia_colonus_MBM18304_PA_Veraguas_W
Colonia_colonus_USNM279_PA_BdelToro_W
Colonia_colonus_LSUMZ5945_EC_MoronaS_E
Colonia_colonus_MPEGMPDS0432_BR_Roraima_E
Colonia_colonus_USNM9288_GY_Northwest_E
Sublegatus_modestus_LSUMZ137586_OG
Empidonax_flavescens_f_MVZ178156_OG
0.97
1
1
1
1
1
0.95
1
1
0.59
40
30
20
10
0
Million years before present (Ma)

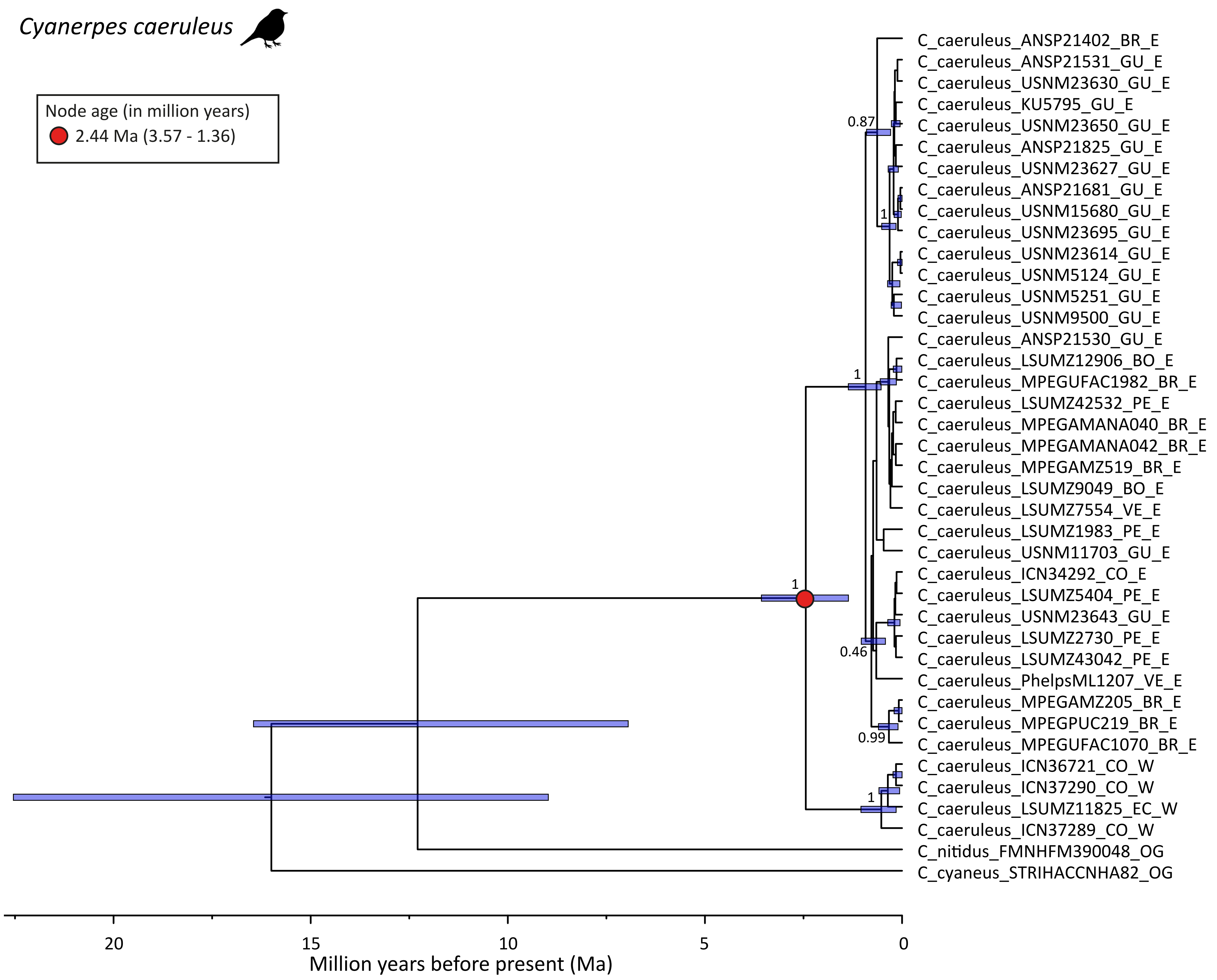

Cyanerpes caeruleus
Node age (in million years)
2.44 Ma (3.57 - 1.36)
C_caeruleus_ANSP21402_BR_E
C_caeruleus_ANSP21531_GU_E
C_caeruleus_USNM23630_GU_E
C_caeruleus_KU5795_GU_E
C_caeruleus_USNM23650_GU_E
C_caeruleus_ANSP21825_GU_E
C_caeruleus_USNM23627_GU_E
C_caeruleus_ANSP21681_GU_E
C_caeruleus_USNM15680_GU_E
C_caeruleus_USNM23695_GU_E
C_caeruleus_USNM23614_GU_E
C_caeruleus_USNM5124_GU_E
C_caeruleus_USNM5251_GU_E
C_caeruleus_USNM9500_GU_E
C_caeruleus_ANSP21530_GU_E
C_caeruleus_LSUMZ12906_BO_E
C_caeruleus_MPEGUFAC1982_BR_E
C_caeruleus_LSUMZ42532_PE_E
C_caeruleus_MPEGAMANA040_BR_E
C_caeruleus_MPEGAMANA042_BR_E
C_caeruleus_MPEGAMZ519_BR_E
C_caeruleus_LSUMZ9049_BO_E
C_caeruleus_LSUMZ7554_VE_E
C_caeruleus_LSUMZ1983_PE_E
C_caeruleus_USNM11703_GU_E
C_caeruleus_ICN34292_CO_E
C_caeruleus_LSUMZ5404_PE_E
C_caeruleus_USNM23643_GU_E
C_caeruleus_LSUMZ2730_PE_E
C_caeruleus_LSUMZ43042_PE_E
C_caeruleus_PhelpsML1207_VE_E
C_caeruleus_MPEGAMZ205_BR_E
C_caeruleus_MPEGPUC219_BR_E
C_caeruleus_MPEGUFAC1070_BR_E
C_caeruleus_ICN36721_CO_W
C_caeruleus_ICN37290_CO_W
C_caeruleus_LSUMZ11825_EC_W
C_caeruleus_ICN37289_CO_W
C_nitidus_FMNHFM390048_OG
C_cyaneus_STRIHACCNHA82_OG
0.87
1
1
1
0.46
0.99
1
20
15
10
5
0
Million years before present (Ma)

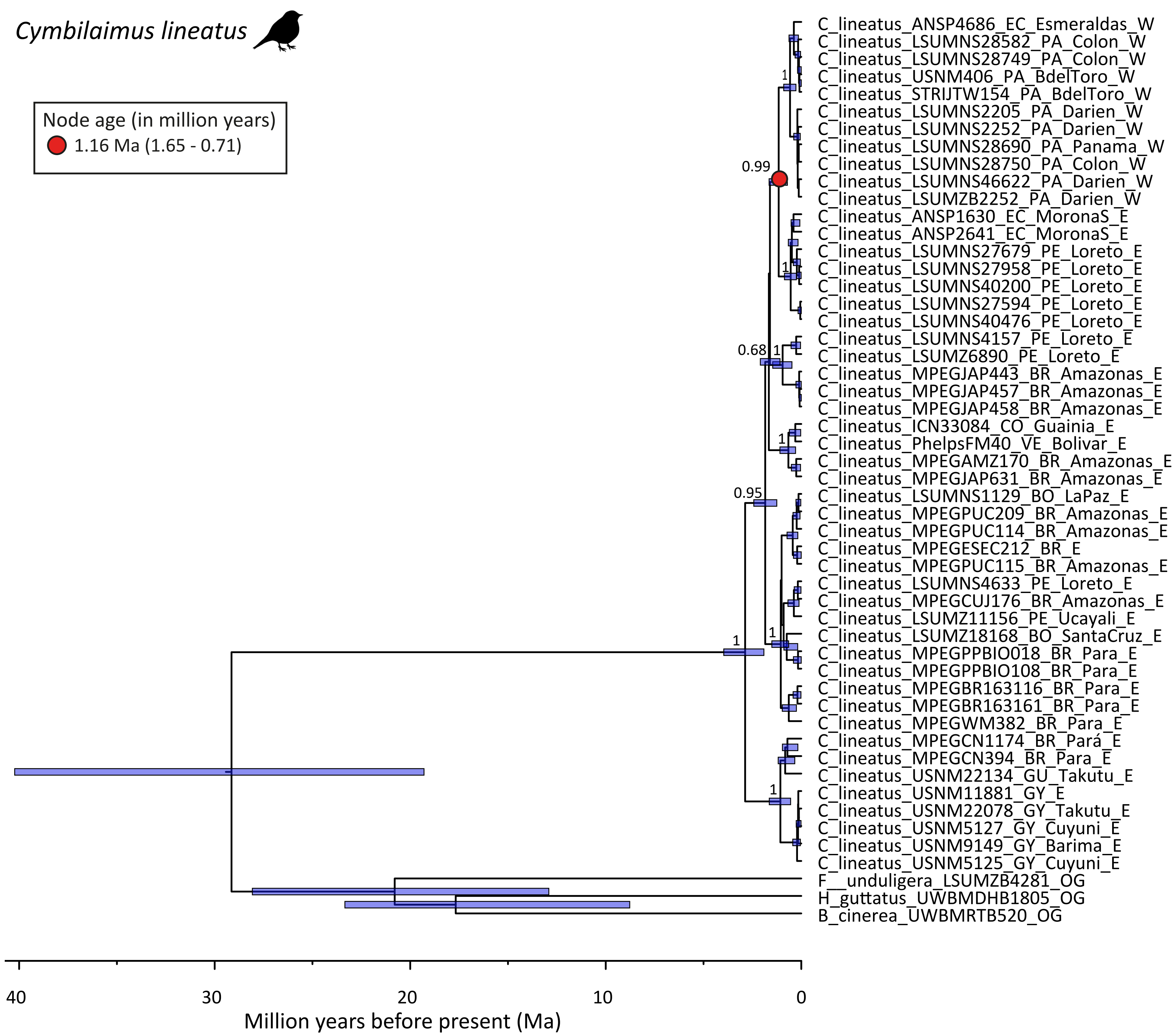

Cymbilaimus lineatus
Node age (in million years)
1.16 Ma (1.65 - 0.71)
C_lineatus_ANSP4686_EC_Esmeraldas_W
C_lineatus_LSUMNS28582_PA_Colon_W
C_lineatus_LSUMNS28749_PA_Colon_W
C_lineatus_USNM406_PA_BdelToro_W
C_lineatus_STRIJTW154_PA_BdelToro_W
C_lineatus_LSUMNS2205_PA_Darien_W
C_lineatus_LSUMNS2252_PA_Darien_W
C_lineatus_LSUMNS28690_PA_Panama_W
C_lineatus_LSUMNS28750_PA_Colon_W
C_lineatus_LSUMNS46622_PA_Darien_W
C_lineatus_LSUMZB2252_PA_Darien_W
C_lineatus_ANSP1630_EC_MoronaS_E
C_lineatus_ANSP2641_EC_MoronaS_E
C_lineatus_LSUMNS27679_PE_Loreto_E
C_lineatus_LSUMNS27958_PE_Loreto_E
C_lineatus_LSUMNS40200_PE_Loreto_E
C_lineatus_LSUMNS27594_PE_Loreto_E
C_lineatus_LSUMNS40476_PE_Loreto_E
C_lineatus_LSUMNS4157_PE_Loreto_E
C_lineatus_LSUMZ6890_PE_Loreto_E
C_lineatus_MPEGJAP443_BR_Amazonas_E
C_lineatus_MPEGJAP457_BR_Amazonas_E
C_lineatus_MPEGJAP458_BR_Amazonas_E
C_lineatus_ICN33084_CO_Guainia_E
C_lineatus_PhelpsFM40_VE_Bolivar_E
C_lineatus_MPEGAMZ170_BR_Amazonas_E
C_lineatus_MPEGJAP631_BR_Amazonas_E
C_lineatus_LSUMNS1129_BO_LaPaz_E
C_lineatus_MPEGPUC209_BR_Amazonas_E
C_lineatus_MPEGPUC114_BR_Amazonas_E
C_lineatus_MPEGESEC212_BR_E
C_lineatus_MPEGPUC115_BR_Amazonas_E
C_lineatus_LSUMNS4633_PE_Loreto_E
C_lineatus_MPEGCUJ176_BR_Amazonas_E
C_lineatus_LSUMZ11156_PE_Ucayali_E
C_lineatus_LSUMZ18168_BO_SantaCruz_E
C_lineatus_MPEGPPBIO018_BR_Para_E
C_lineatus_MPEGPPBIO108_BR_Para_E
C_lineatus_MPEGBR163116_BR_Para_E
C_lineatus_MPEGBR163161_BR_Para_E
C_lineatus_MPEGWM382_BR_Para_E
C_lineatus_MPEGCN1174_BR_Pará_E
C_lineatus_MPEGCN394_BR_Para_E
C_lineatus_USNM22134_GU_Takutu_E
C_lineatus_USNM11881_GY_E
C_lineatus_USNM22078_GY_Takutu_E
C_lineatus_USNM5127_GY_Cuyuni_E
C_lineatus_USNM9149_GY_Barima_E
C_lineatus_USNM5125_GY_Cuyuni_E
F__unduligera_LSUMZB4281_OG
H_guttatus_UWBMDHB1805_OG
B_cinerea_UWBMRTB520_OG
1
0.99
1
0.68
1
1
0.95
1
1
1
1
40
30
20
10
0
Million years before present (Ma)

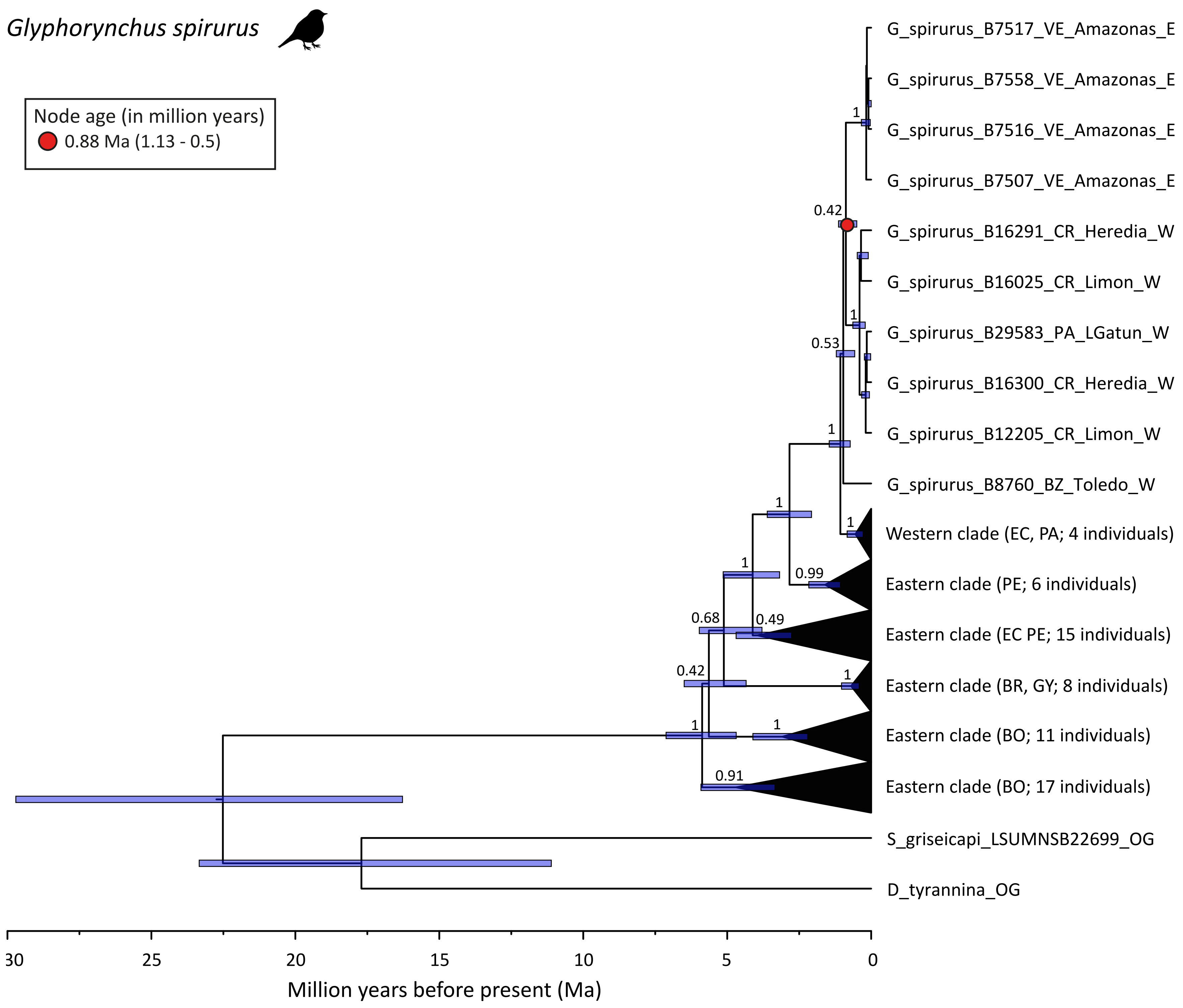

Glyphorynchus spirurus
Node age (in million years)
0.88 Ma (1.13 - 0.5)
G_spirurus_B7517_VE_Amazonas_E
G_spirurus_B7558_VE_Amazonas_E
G_spirurus_B7516_VE_Amazonas_E
G_spirurus_B7507_VE_Amazonas_E
G_spirurus_B16291_CR_Heredia_W
G_spirurus_B16025_CR_Limon_W
G_spirurus_B29583_PA_LGatun_W
G_spirurus_B16300_CR_Heredia_W
G_spirurus_B12205_CR_Limon_W
G_spirurus_B8760_BZ_Toledo_W
Western clade (EC, PA; 4 individuals)
Eastern clade (PE; 6 individuals)
Eastern clade (EC PE; 15 individuals)
Eastern clade (BR, GY; 8 individuals)
Eastern clade (BO; 11 individuals)
Eastern clade (BO; 17 individuals)
S_griseicapi_LSUMNSB22699_OG
D_tyrannina_OG
1
0.42
1
0.53
1
1
1
0.99
0.68
0.49
0.42
1
1
1
0.91
1
30
25
20
15
10
5
0
Million years before present (Ma)

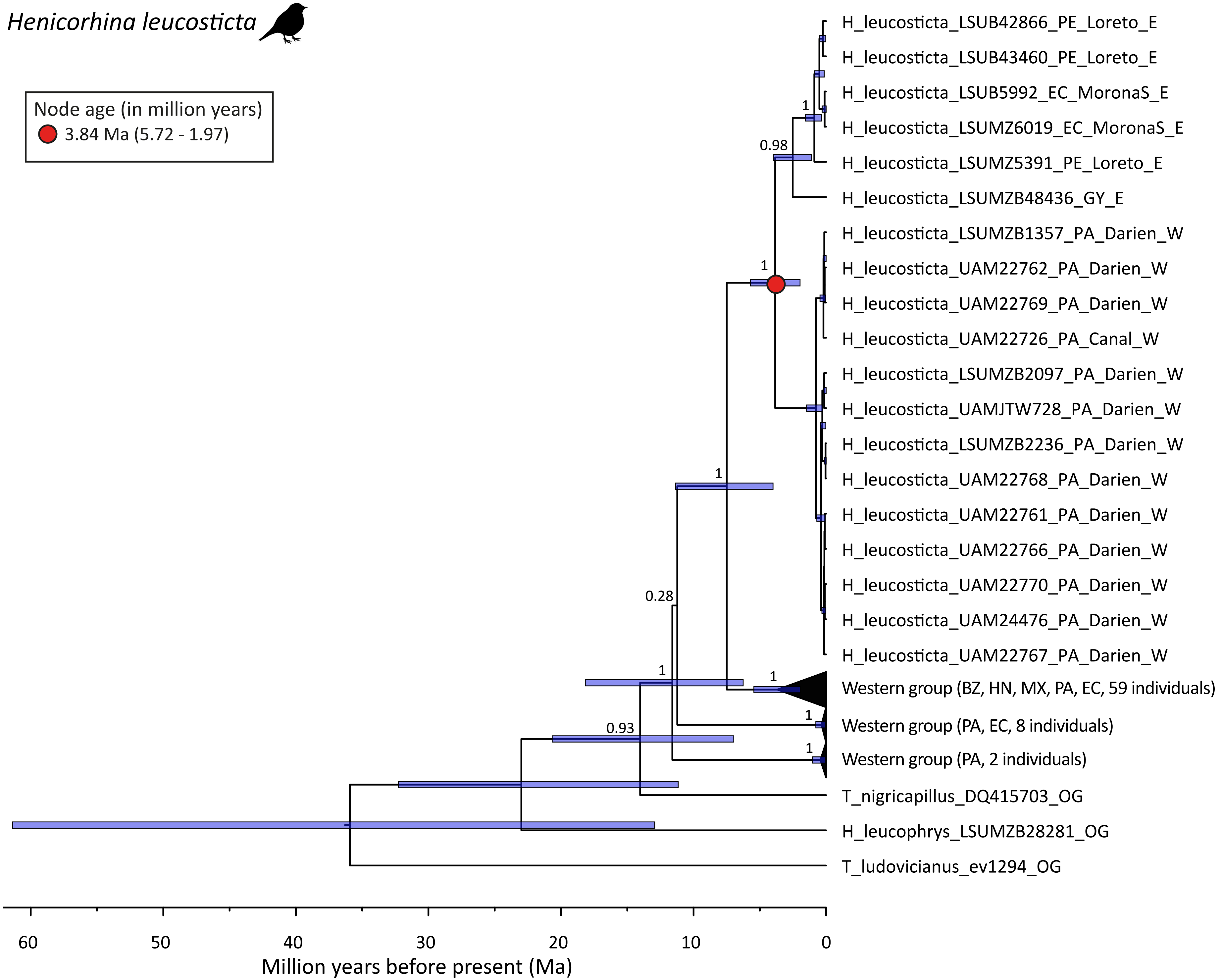
Henicorhina leucosticta
Node age (in million years)
3.84 Ma (5.72 - 1.97)
H_leucosticta_LSUB42866_PE_Loreto_E
H_leucosticta_LSUB43460_PE_Loreto_E
H_leucosticta_LSUB5992_EC_MoronaS_E
H_leucosticta_LSUMZ6019_EC_MoronaS_E
H_leucosticta_LSUMZ5391_PE_Loreto_E
H_leucosticta_LSUMZB48436_GY_E
H_leucosticta_LSUMZB1357_PA_Darien_W
H_leucosticta_UAM22762_PA_Darien_W
H_leucosticta_UAM22769_PA_Darien_W
H_leucosticta_UAM22726_PA_Canal_W
H_leucosticta_LSUMZB2097_PA_Darien_W
H_leucosticta_UAMJTW728_PA_Darien_W
H_leucosticta_LSUMZB2236_PA_Darien_W
H_leucosticta_UAM22768_PA_Darien_W
H_leucosticta_UAM22761_PA_Darien_W
H_leucosticta_UAM22766_PA_Darien_W
H_leucosticta_UAM22770_PA_Darien_W
H_leucosticta_UAM24476_PA_Darien_W
H_leucosticta_UAM22767_PA_Darien_W
Western group (BZ, HN, MX, PA, EC, 59 individuals)
Western group (PA, EC, 8 individuals)
Western group (PA, 2 individuals)
T_nigricapillus_DQ415703_OG
H_leucophrys_LSUMZB28281_OG
T_ludovicianus_ev1294_OG
1
0.98
1
1
0.28
1
1
1
0.93
1
60
50
40
30
20
10
0
Million years before present (Ma)

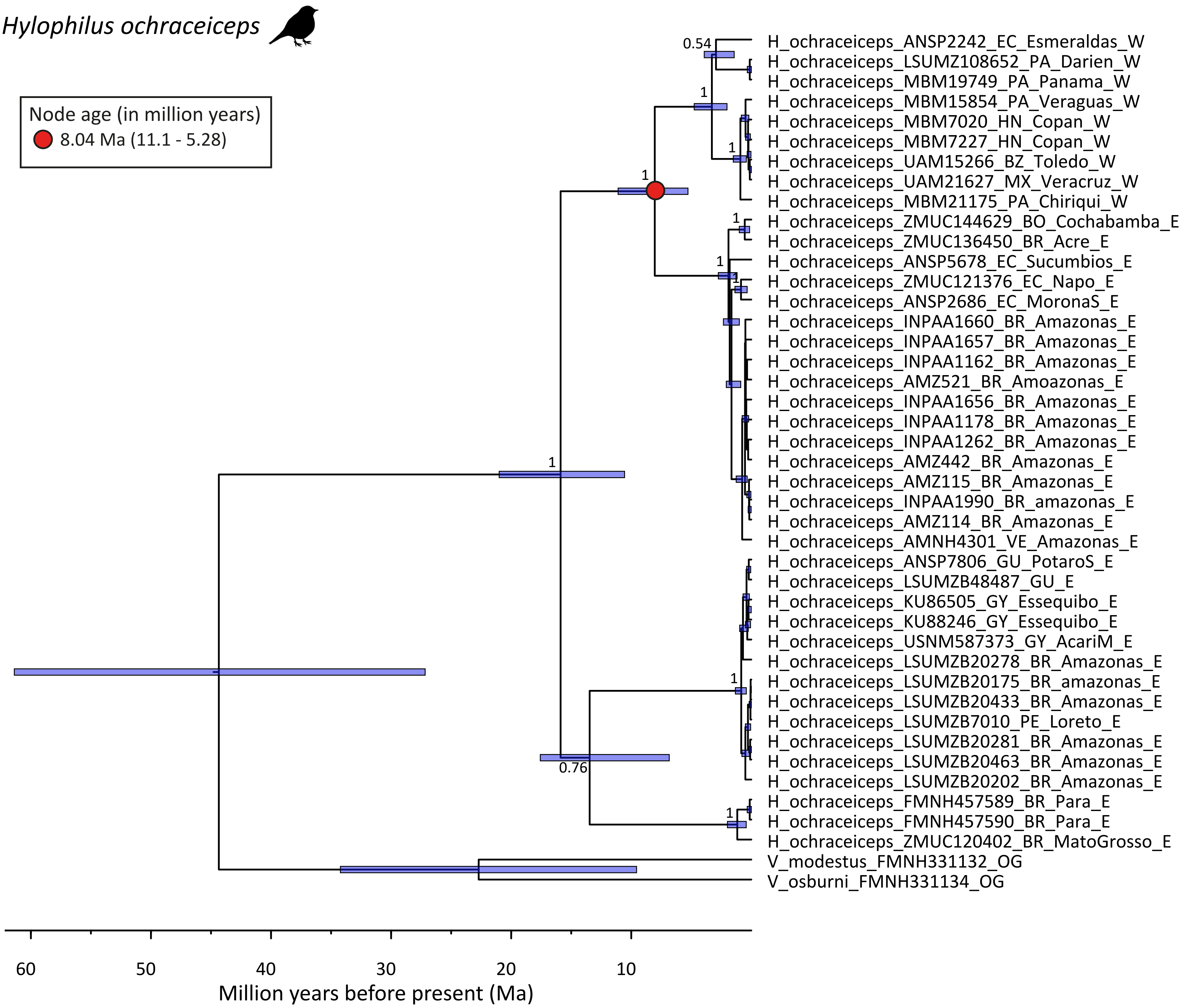

Hylophilus ochraceiceps
Node age (in million years)
8.04 Ma (11.1 - 5.28)
0.54
1
0.76
H_ochraceiceps_ANSP2242_EC_Esmeraldas_W
H_ochraceiceps_LSUMZ108652_PA_Darien_W
H_ochraceiceps_MBM19749_PA_Panama_W
H_ochraceiceps_MBM15854_PA_Veraguas_W
H_ochraceiceps_MBM7020_HN_Copan_W
H_ochraceiceps_MBM7227_HN_Copan_W
H_ochraceiceps_UAM15266_BZ_Toledo_W
H_ochraceiceps_UAM21627_MX_Veracruz_W
H_ochraceiceps_MBM21175_PA_Chiriqui_W
H_ochraceiceps_ZMUC144629_BO_Cochabamba_E
H_ochraceiceps_ZMUC136450_BR_Acre_E
H_ochraceiceps_ANSP5678_EC_Sucumbios_E
H_ochraceiceps_ZMUC121376_EC_Napo_E
H_ochraceiceps_ANSP2686_EC_MoronaS_E
H_ochraceiceps_INPAA1660_BR_Amazonas_E
H_ochraceiceps_INPAA1657_BR_Amazonas_E
H_ochraceiceps_INPAA1162_BR_Amazonas_E
H_ochraceiceps_AMZ521_BR_Amoazonas_E
H_ochraceiceps_INPAA1656_BR_Amazonas_E
H_ochraceiceps_INPAA1178_BR_Amazonas_E
H_ochraceiceps_INPAA1262_BR_Amazonas_E
H_ochraceiceps_AMZ442_BR_Amazonas_E
H_ochraceiceps_AMZ115_BR_Amazonas_E
H_ochraceiceps_INPAA1990_BR_amazonas_E
H_ochraceiceps_AMZ114_BR_Amazonas_E
H_ochraceiceps_AMNH4301_VE_Amazonas_E
H_ochraceiceps_ANSP7806_GU_PotaroS_E
H_ochraceiceps_LSUMZB48487_GU_E
H_ochraceiceps_KU86505_GY_Essequibo_E
H_ochraceiceps_KU88246_GY_Essequibo_E
H_ochraceiceps_USNM587373_GY_AcariM_E
H_ochraceiceps_LSUMZB20278_BR_Amazonas_E
H_ochraceiceps_LSUMZB20175_BR_amazonas_E
H_ochraceiceps_LSUMZB20433_BR_Amazonas_E
H_ochraceiceps_LSUMZB7010_PE_Loreto_E
H_ochraceiceps_LSUMZB20281_BR_Amazonas_E
H_ochraceiceps_LSUMZB20463_BR_Amazonas_E
H_ochraceiceps_LSUMZB20202_BR_Amazonas_E
H_ochraceiceps_FMNH457589_BR_Para_E
H_ochraceiceps_FMNH457590_BR_Para_E
H_ochraceiceps_ZMUC120402_BR_MatoGrosso_E
V_modestus_FMNH331132_OG
V_osburni_FMNH331134_OG
60
50
40
30
20
10
Million years before present (Ma)

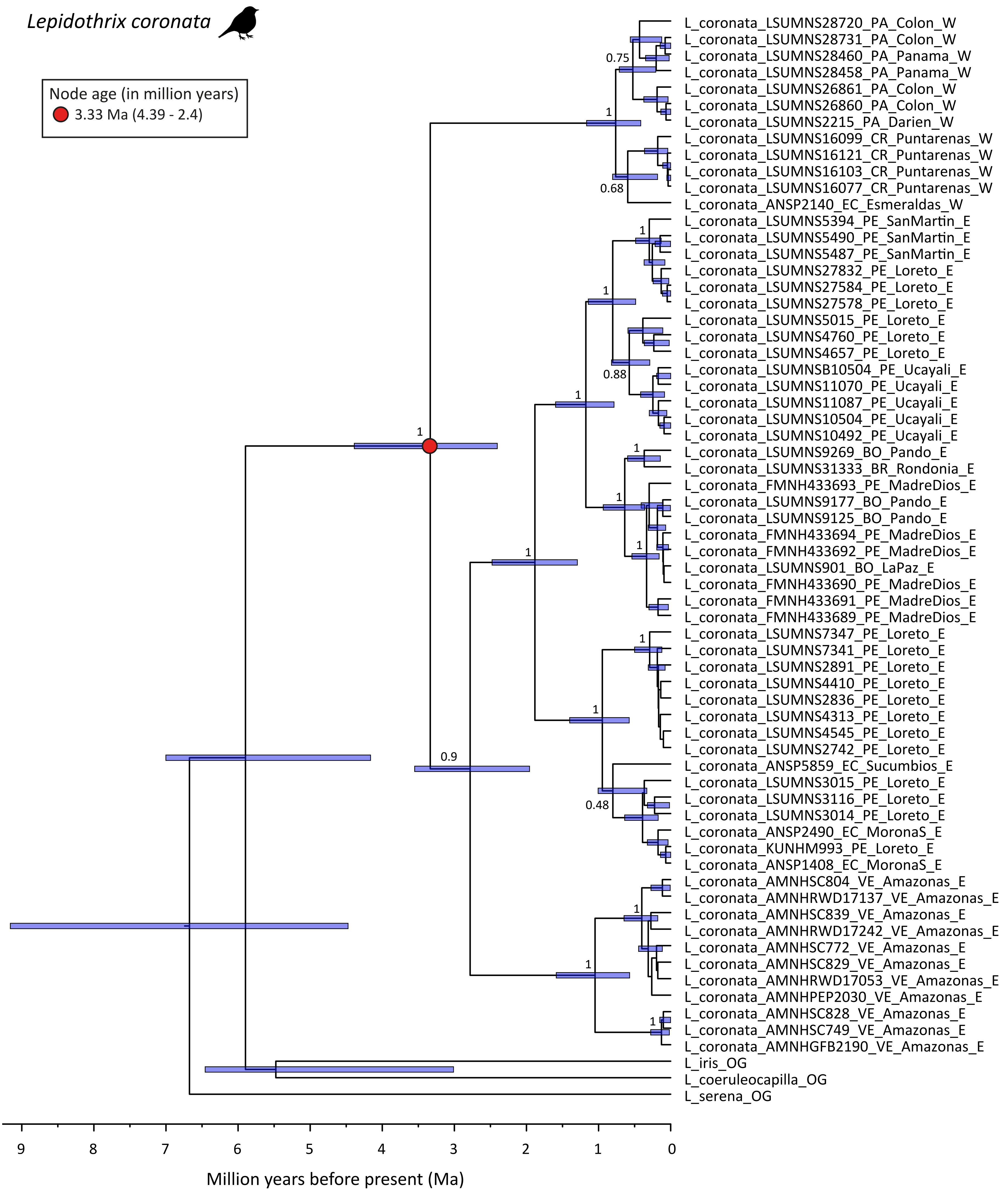
Lepidothrix coronata
Node age (in million years)
3.33 Ma (4.39 - 2.4)
L_coronata_LSUMNS28720_PA_Colon_W
L_coronata_LSUMNS28731_PA_Colon_W
L_coronata_LSUMNS28460_PA_Panama_W
L_coronata_LSUMNS28458_PA_Panama_W
L_coronata_LSUMNS26861_PA_Colon_W
L_coronata_LSUMNS26860_PA_Colon_W
L_coronata_LSUMNS2215_PA_Darien_W
L_coronata_LSUMNS16099_CR_Puntarenas_W
L_coronata_LSUMNS16121_CR_Puntarenas_W
L_coronata_LSUMNS16103_CR_Puntarenas_W
L_coronata_LSUMNS16077_CR_Puntarenas_W
L_coronata_ANSP2140_EC_Esmeraldas_W
L_coronata_LSUMNS5394_PE_SanMartin_E
L_coronata_LSUMNS5490_PE_SanMartin_E
L_coronata_LSUMNS5487_PE_SanMartin_E
L_coronata_LSUMNS27832_PE_Loreto_E
L_coronata_LSUMNS27584_PE_Loreto_E
L_coronata_LSUMNS27578_PE_Loreto_E
L_coronata_LSUMNS5015_PE_Loreto_E
L_coronata_LSUMNS4760_PE_Loreto_E
L_coronata_LSUMNS4657_PE_Loreto_E
L_coronata_LSUMNSB10504_PE_Ucayali_E
L_coronata_LSUMNS11070_PE_Ucayali_E
L_coronata_LSUMNS11087_PE_Ucayali_E
L_coronata_LSUMNS10504_PE_Ucayali_E
L_coronata_LSUMNS10492_PE_Ucayali_E
L_coronata_LSUMNS9269_BO_Pando_E
L_coronata_LSUMNS31333_BR_Rondonia_E
L_coronata_FMNH433693_PE_MadreDios_E
L_coronata_LSUMNS9177_BO_Pando_E
L_coronata_LSUMNS9125_BO_Pando_E
L_coronata_FMNH433694_PE_MadreDios_E
L_coronata_FMNH433692_PE_MadreDios_E
L_coronata_LSUMNS901_BO_LaPaz_E
L_coronata_FMNH433690_PE_MadreDios_E
L_coronata_FMNH433691_PE_MadreDios_E
L_coronata_FMNH433689_PE_MadreDios_E
L_coronata_LSUMNS7347_PE_Loreto_E
L_coronata_LSUMNS7341_PE_Loreto_E
L_coronata_LSUMNS2891_PE_Loreto_E
L_coronata_LSUMNS4410_PE_Loreto_E
L_coronata_LSUMNS2836_PE_Loreto_E
L_coronata_LSUMNS4313_PE_Loreto_E
L_coronata_LSUMNS4545_PE_Loreto_E
L_coronata_LSUMNS2742_PE_Loreto_E
L_coronata_ANSP5859_EC_Sucumbios_E
L_coronata_LSUMNS3015_PE_Loreto_E
L_coronata_LSUMNS3116_PE_Loreto_E
L_coronata_LSUMNS3014_PE_Loreto_E
L_coronata_ANSP2490_EC_MoronaS_E
L_coronata_KUNHM993_PE_Loreto_E
L_coronata_ANSP1408_EC_MoronaS_E
L_coronata_AMNHSC804_VE_Amazonas_E
L_coronata_AMNHRWD17137_VE_Amazonas_E
L_coronata_AMNHSC839_VE_Amazonas_E
L_coronata_AMNHRWD17242_VE_Amazonas_E
L_coronata_AMNHSC772_VE_Amazonas_E
L_coronata_AMNHSC829_VE_Amazonas_E
L_coronata_AMNHRWD17053_VE_Amazonas_E
L_coronata_AMNHPEP2030_VE_Amazonas_E
L_coronata_AMNHSC828_VE_Amazonas_E
L_coronata_AMNHSC749_VE_Amazonas_E
L_coronata_AMNHGFB2190_VE_Amazonas_E
L_iris_OG
L_coeruleocapilla_OG
L_serena_OG
0.75
1
0.68
1
1
0.88
1
1
1
1
1
1
1
1
0.9
1
0.48
1
1
1
9
8
7
6
5
4
3
2
1
0
Million years before present (Ma)

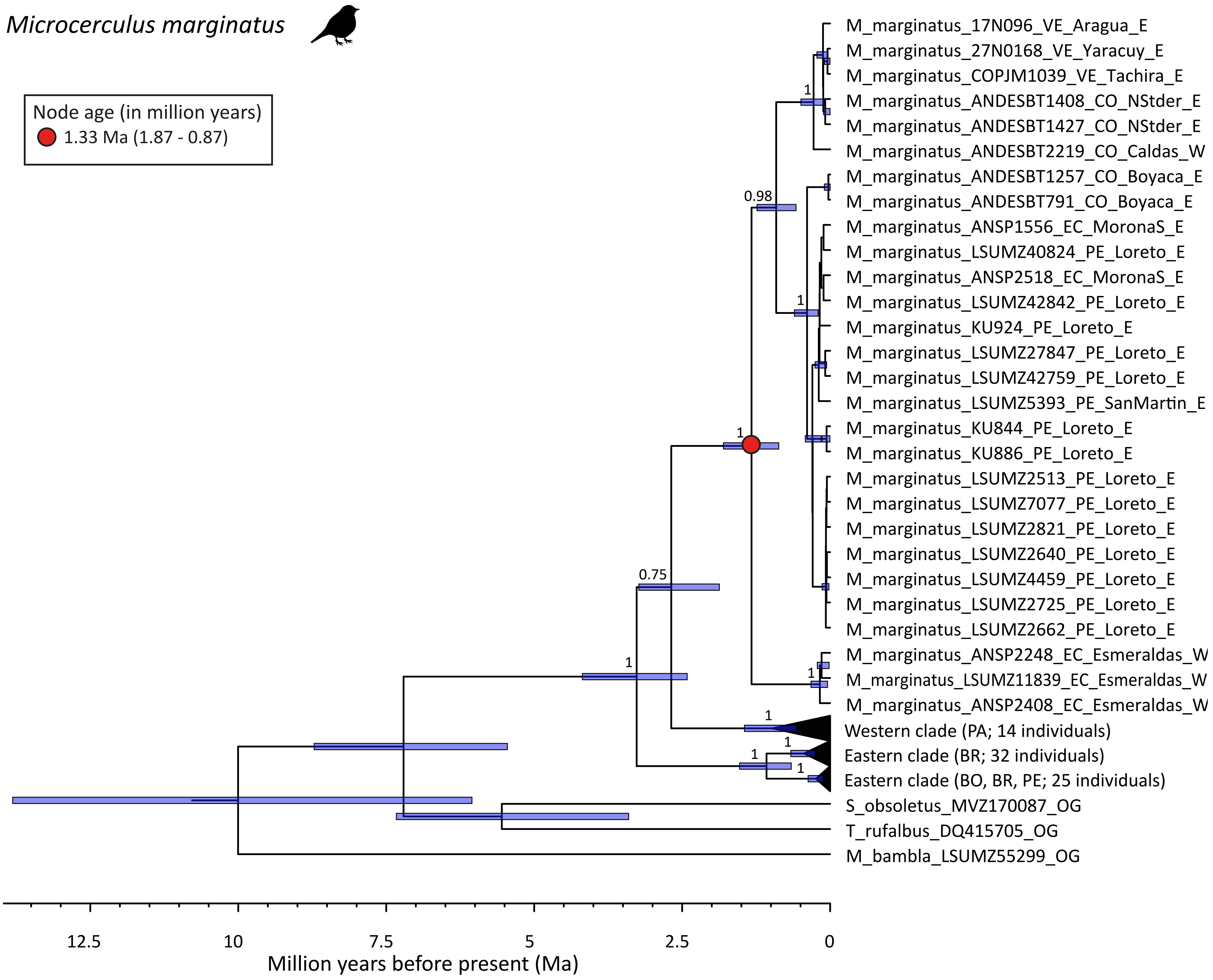

Microcerculus marginatus
Node age (in million years)
1.33 Ma (1.87 - 0.87)
M_marginatus_17N096_VE_Aragua_E
M_marginatus_27N0168_VE_Yaracuy_E
M_marginatus_COPJM1039_VE_Tachira_E
M_marginatus_ANDESBT1408_CO_NStder_E
M_marginatus_ANDESBT1427_CO_NStder_E
M_marginatus_ANDESBT2219_CO_Caldas_W
M_marginatus_ANDESBT1257_CO_Boyaca_E
M_marginatus_ANDESBT791_CO_Boyaca_E
M_marginatus_ANSP1556_EC_MoronaS_E
M_marginatus_LSUMZ40824_PE_Loreto_E
M_marginatus_ANSP2518_EC_MoronaS_E
M_marginatus_LSUMZ42842_PE_Loreto_E
M_marginatus_KU924_PE_Loreto_E
M_marginatus_LSUMZ27847_PE_Loreto_E
M_marginatus_LSUMZ42759_PE_Loreto_E
M_marginatus_LSUMZ5393_PE_SanMartin_E
M_marginatus_KU844_PE_Loreto_E
M_marginatus_KU886_PE_Loreto_E
M_marginatus_LSUMZ2513_PE_Loreto_E
M_marginatus_LSUMZ7077_PE_Loreto_E
M_marginatus_LSUMZ2821_PE_Loreto_E
M_marginatus_LSUMZ2640_PE_Loreto_E
M_marginatus_LSUMZ4459_PE_Loreto_E
M_marginatus_LSUMZ2725_PE_Loreto_E
M_marginatus_LSUMZ2662_PE_Loreto_E
M_marginatus_ANSP2248_EC_Esmeraldas_W
M_marginatus_LSUMZ11839_EC_Esmeraldas_W
M_marginatus_ANSP2408_EC_Esmeraldas_W
Western clade (PA; 14 individuals)
Eastern clade (BR; 32 individuals)
Eastern clade (BO, BR, PE; 25 individuals)
S_obsoletus_MVZ170087_OG
T_rufalbus_DQ415705_OG
M_bambla_LSUMZ55299_OG
1
0.98
1
1
0.75
1
1
1
1
1
1
12.5
10
7.5
5
2.5
0
Million years before present (Ma)

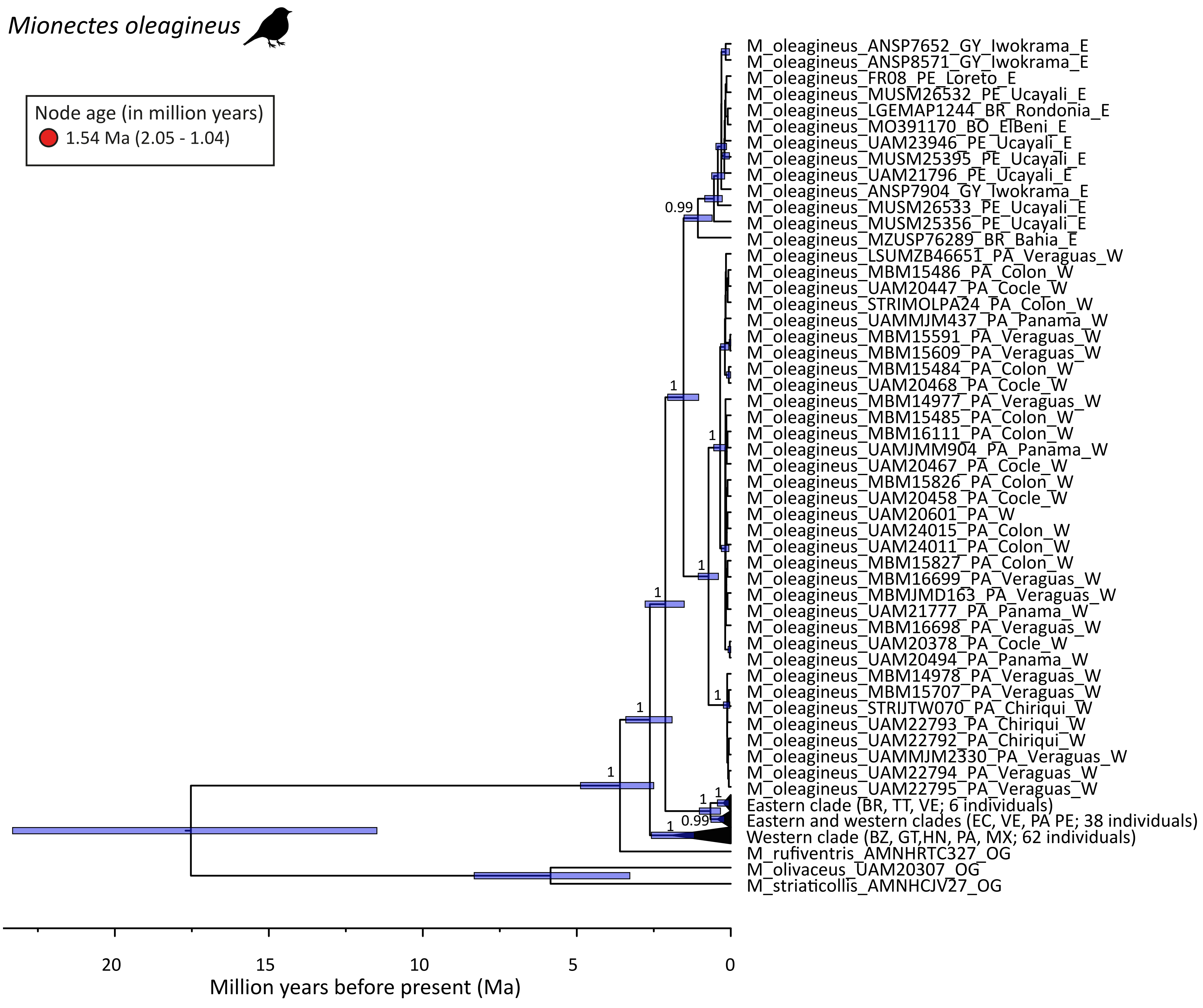

Mionectes oleagineus
Node age (in million years)
1.54 Ma (2.05 - 1.04)
M_oleagineus_ANSP7652_GY_Iwokrama_E
M_oleagineus_ANSP8571_GY_Iwokrama_E
M_oleagineus_FR08_PE_Loreto_E
M_oleagineus_MUSM26532_PE_Ucayali_E
M_oleagineus_LGEMAP1244_BR_Rondonia_E
M_oleagineus_MO391170_BO_ElBeni_E
M_oleagineus_UAM23946_PE_Ucayali_E
M_oleagineus_MUSM25395_PE_Ucayali_E
M_oleagineus_UAM21796_PE_Ucayali_E
M_oleagineus_ANSP7904_GY_Iwokrama_E
M_oleagineus_MUSM26533_PE_Ucayali_E
M_oleagineus_MUSM25356_PE_Ucayali_E
M_oleagineus_MZUSP76289_BR_Bahia_E
M_oleagineus_LSUMZB46651_PA_Veraguas_W
M_oleagineus_MBM15486_PA_Colon_W
M_oleagineus_UAM20447_PA_Cocle_W
M_oleagineus_STRIMOLPA24_PA_Colon_W
M_oleagineus_UAMMJM437_PA_Panama_W
M_oleagineus_MBM15591_PA_Veraguas_W
M_oleagineus_MBM15609_PA_Veraguas_W
M_oleagineus_MBM15484_PA_Colon_W
M_oleagineus_UAM20468_PA_Cocle_W
M_oleagineus_MBM14977_PA_Veraguas_W
M_oleagineus_MBM15485_PA_Colon_W
M_oleagineus_MBM16111_PA_Colon_W
M_oleagineus_UAMJMM904_PA_Panama_W
M_oleagineus_UAM20467_PA_Cocle_W
M_oleagineus_MBM15826_PA_Colon_W
M_oleagineus_UAM20458_PA_Cocle_W
M_oleagineus_UAM20601_PA_W
M_oleagineus_UAM24015_PA_Colon_W
M_oleagineus_UAM24011_PA_Colon_W
M_oleagineus_MBM15827_PA_Colon_W
M_oleagineus_MBM16699_PA_Veraguas_W
M_oleagineus_MBMJMD163_PA_Veraguas_W
M_oleagineus_UAM21777_PA_Panama_W
M_oleagineus_MBM16698_PA_Veraguas_W
M_oleagineus_UAM20378_PA_Cocle_W
M_oleagineus_UAM20494_PA_Panama_W
M_oleagineus_MBM14978_PA_Veraguas_W
M_oleagineus_MBM15707_PA_Veraguas_W
M_oleagineus_STRIJTW070_PA_Chiriqui_W
M_oleagineus_UAM22793_PA_Chiriqui_W
M_oleagineus_UAM22792_PA_Chiriqui_W
M_oleagineus_UAMMJM2330_PA_Veraguas_W
M_oleagineus_UAM22794_PA_Veraguas_W
M_oleagineus_UAM22795_PA_Veraguas_W
Eastern clade (BR, TT, VE; 6 individuals)
Eastern and western clades (EC, VE, PA PE; 38 individuals)
Western clade (BZ, GT,HN, PA, MX; 62 individuals)
M_rufiventris_AMNHRTC327_OG
M_olivaceus_UAM20307_OG
M_striaticollis_AMNHCJV27_OG
0.99
1
1
1
1
1
1
1
1
0.99
1
20
15
10
5
0
Million years before present (Ma)

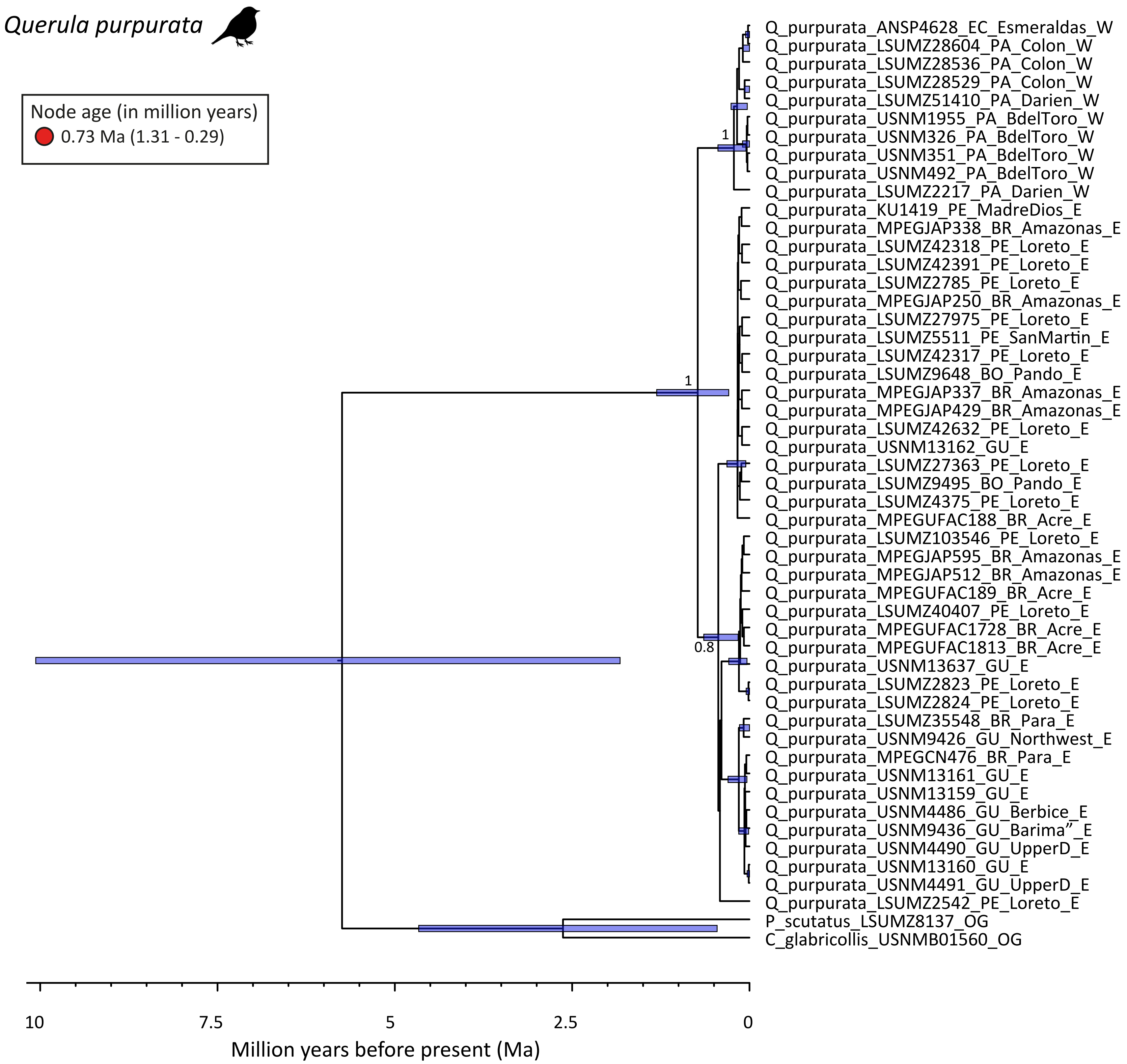
Querula purpurata
Node age (in million years)
0.73 Ma (1.31 - 0.29)
1
1
0.8
Q_purpurata_ANSP4628_EC_Esmeraldas_W
Q_purpurata_LSUMZ28604_PA_Colon_W
Q_purpurata_LSUMZ28536_PA_Colon_W
Q_purpurata_LSUMZ28529_PA_Colon_W
Q_purpurata_LSUMZ51410_PA_Darien_W
Q_purpurata_USNM1955_PA_BdelToro_W
Q_purpurata_USNM326_PA_BdelToro_W
Q_purpurata_USNM351_PA_BdelToro_W
Q_purpurata_USNM492_PA_BdelToro_W
Q_purpurata_LSUMZ2217_PA_Darien_W
Q_purpurata_KU1419_PE_MadreDios_E
Q_purpurata_MPEGJAP338_BR_Amazonas_E
Q_purpurata_LSUMZ42318_PE_Loreto_E
Q_purpurata_LSUMZ42391_PE_Loreto_E
Q_purpurata_LSUMZ2785_PE_Loreto_E
Q_purpurata_MPEGJAP250_BR_Amazonas_E
Q_purpurata_LSUMZ27975_PE_Loreto_E
Q_purpurata_LSUMZ5511_PE_SanMartin_E
Q_purpurata_LSUMZ42317_PE_Loreto_E
Q_purpurata_LSUMZ9648_BO_Pando_E
Q_purpurata_MPEGJAP337_BR_Amazonas_E
Q_purpurata_MPEGJAP429_BR_Amazonas_E
Q_purpurata_LSUMZ42632_PE_Loreto_E
Q_purpurata_USNM13162_GU_E
Q_purpurata_LSUMZ27363_PE_Loreto_E
Q_purpurata_LSUMZ9495_BO_Pando_E
Q_purpurata_LSUMZ4375_PE_Loreto_E
Q_purpurata_MPEGUFAC188_BR_Acre_E
Q_purpurata_LSUMZ103546_PE_Loreto_E
Q_purpurata_MPEGJAP595_BR_Amazonas_E
Q_purpurata_MPEGJAP512_BR_Amazonas_E
Q_purpurata_MPEGUFAC189_BR_Acre_E
Q_purpurata_LSUMZ40407_PE_Loreto_E
Q_purpurata_MPEGUFAC1728_BR_Acre_E
Q_purpurata_MPEGUFAC1813_BR_Acre_E
Q_purpurata_USNM13637_GU_E
Q_purpurata_LSUMZ2823_PE_Loreto_E
Q_purpurata_LSUMZ2824_PE_Loreto_E
Q_purpurata_LSUMZ35548_BR_Para_E
Q_purpurata_USNM9426_GU_Northwest_E
Q_purpurata_MPEGCN476_BR_Para_E
Q_purpurata_USNM13161_GU_E
Q_purpurata_USNM13159_GU_E
Q_purpurata_USNM4486_GU_Berbice_E
Q_purpurata_USNM9436_GU_Barima"_E
Q_purpurata_USNM4490_GU_UpperD_E
Q_purpurata_USNM13160_GU_E
Q_purpurata_USNM4491_GU_UpperD_E
Q_purpurata_LSUMZ2542_PE_Loreto_E
P_scutatus_LSUMZ8137_OG
C_glabricollis_USNMB01560_OG
10
7.5
5
2.5
0
Million years before present (Ma)

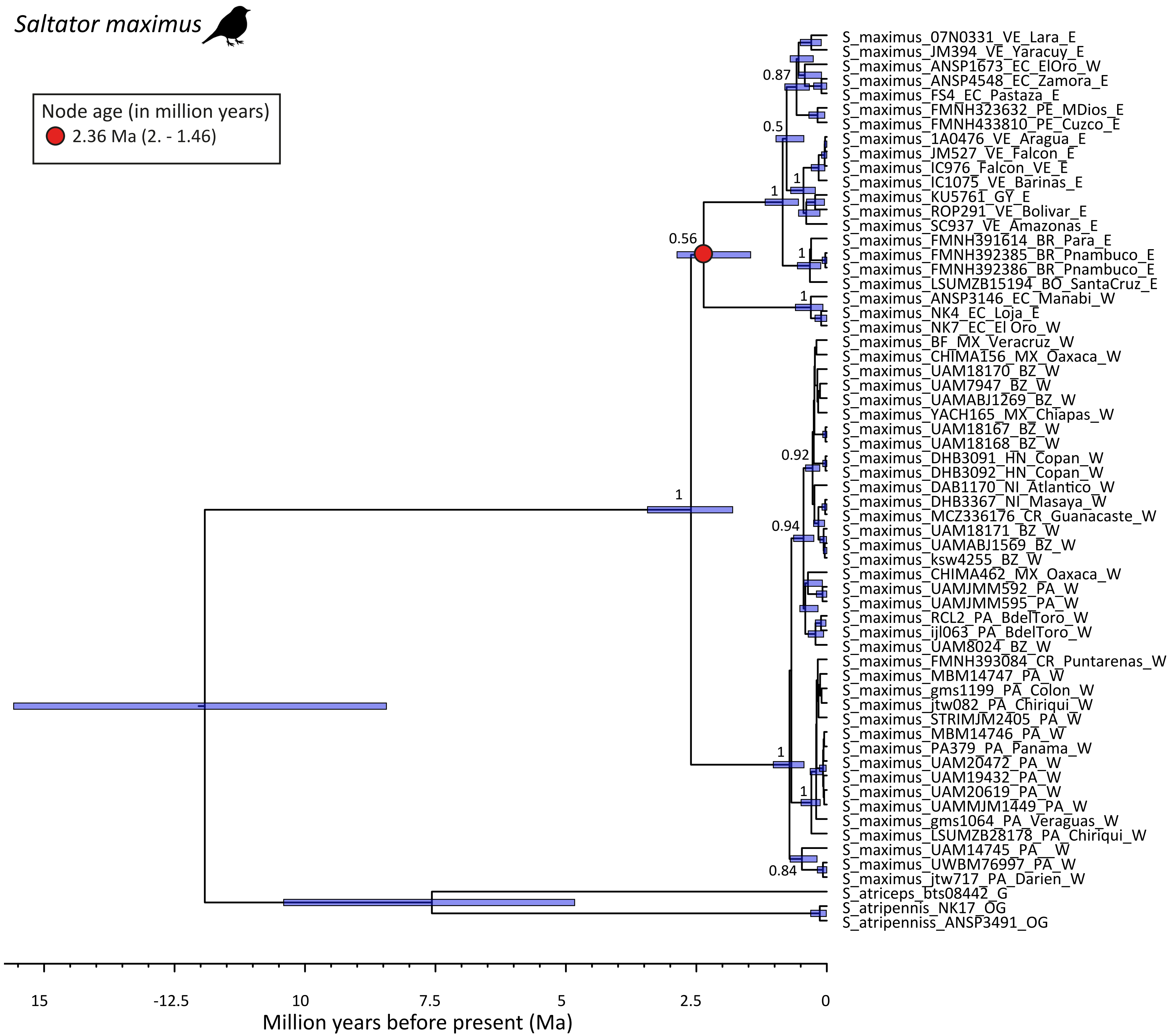

Saltator maximus
Node age (in million years)
2.36 Ma (2. - 1.46)
S_maximus_07N0331_VE_Lara_E
S_maximus_JM394_VE_Yaracuy_E
S_maximus_ANSP1673_EC_ElOro_W
S_maximus_ANSP4548_EC_Zamora_E
S_maximus_FS4_EC_Pastaza_E
S_maximus_FMNH323632_PE_MDios_E
S_maximus_FMNH433810_PE_Cuzco_E
S_maximus_1A0476_VE_Aragua_E
S_maximus_JM527_VE_Falcon_E
S_maximus_IC976_Falcon_VE_E
S_maximus_IC1075_VE_Barinas_E
S_maximus_KU5761_GY_E
S_maximus_ROP291_VE_Bolivar_E
S_maximus_SC937_VE_Amazonas_E
S_maximus_FMNH391614_BR_Para_E
S_maximus_FMNH392385_BR_Pnambuco_E
S_maximus_FMNH392386_BR_Pnambuco_E
S_maximus_LSUMZB15194_BO_SantaCruz_E
S_maximus_ANSP3146_EC_Manabi_W
S_maximus_NK4_EC_Loja_E
S_maximus_NK7_EC_El Oro_W
S_maximus_BF_MX_Veracruz_W
S_maximus_CHIMA156_MX_Oaxaca_W
S_maximus_UAM18170_BZ_W
S_maximus_UAM7947_BZ_W
S_maximus_UAMABJ1269_BZ_W
S_maximus_YACH165_MX_Chiapas_W
S_maximus_UAM18167_BZ_W
S_maximus_UAM18168_BZ_W
S_maximus_DHB3091_HN_Copan_W
S_maximus_DHB3092_HN_Copan_W
S_maximus_DAB1170_NI_Atlantico_W
S_maximus_DHB3367_NI_Masaya_W
S_maximus_MCZ336176_CR_Guanacaste_W
S_maximus_UAM18171_BZ_W
S_maximus_UAMABJ1569_BZ_W
S_maximus_ksw4255_BZ_W
S_maximus_CHIMA462_MX_Oaxaca_W
S_maximus_UAMJMM592_PA_W
S_maximus_UAMJMM595_PA_W
S_maximus_RCL2_PA_BdelToro_W
S_maximus_ijl063_PA_BdelToro_W
S_maximus_UAM8024_BZ_W
S_maximus_FMNH393084_CR_Puntarenas_W
S_maximus_MBM14747_PA_W
S_maximus_gms1199_PA_Colon_W
S_maximus_jtw082_PA_Chiriqui_W
S_maximus_STRIMJM2405_PA_W
S_maximus_MBM14746_PA_W
S_maximus_PA379_PA_Panama_W
S_maximus_UAM20472_PA_W
S_maximus_UAM19432_PA_W
S_maximus_UAM20619_PA_W
S_maximus_UAMMJM1449_PA_W
S_maximus_gms1064_PA_Veraguas_W
S_maximus_LSUMZB28178_PA_Chiriqui_W
S_maximus_UAM14745_PA__W
S_maximus_UWBM76997_PA_W
S_maximus_jtw717_PA_Darien_W
S_atriceps_bts08442_G
S_atripennis_NK17_OG
S_atripenniss_ANSP3491_OG
0.87
0.5
1
1
0.56
1
1
1
0.92
0.94
1
1
0.84
15
-12.5
10
7.5
5
2.5
0
Million years before present (Ma)

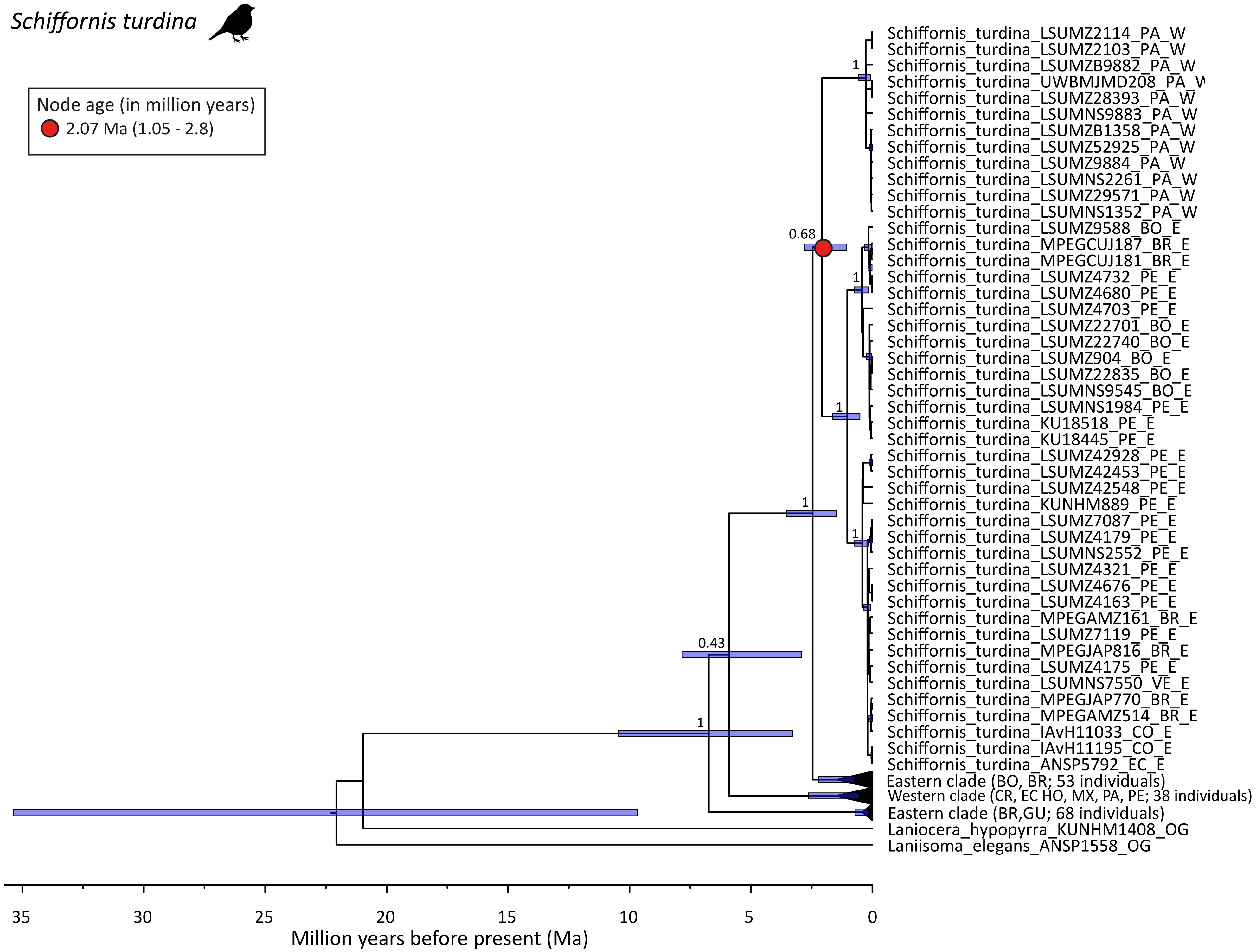

Schiffornis turdina
Node age (in million years)
2.07 Ma (1.05 - 2.8)
Schiffornis_turdina_LSUMZ2114_PA_W
Schiffornis_turdina_LSUMZ2103_PA_W
Schiffornis_turdina_LSUMZB9882_PA_W
Schiffornis_turdina_UWBMJMD208_PA_
Schiffornis_turdina_LSUMZ28393_PA_W
Schiffornis_turdina_LSUMNS9883_PA_W
Schiffornis_turdina_LSUMZB1358_PA_W
Schiffornis_turdina_LSUMZ52925_PA_W
Schiffornis_turdina_LSUMZ9884_PA_W
Schiffornis_turdina_LSUMNS2261_PA_W
Schiffornis_turdina_LSUMZ29571_PA_W
Schiffornis_turdina_LSUMNS1352_PA_W
Schiffornis_turdina_LSUMZ9588_BO_E
Schiffornis_turdina_MPEGCUJ187_BR_E
Schiffornis_turdina_MPEGCUJ181_BR_E
Schiffornis_turdina_LSUMZ4732_PE_E
Schiffornis_turdina_LSUMZ4680_PE_E
Schiffornis_turdina_LSUMZ4703_PE_E
Schiffornis_turdina_LSUMZ22701_BO_E
Schiffornis_turdina_LSUMZ22740_BO_E
Schiffornis_turdina_LSUMZ904_BO_E
Schiffornis_turdina_LSUMZ22835_BO_E
Schiffornis_turdina_LSUMNS9545_BO_E
Schiffornis_turdina_LSUMNS1984_PE_E
Schiffornis_turdina_KU18518_PE_E
Schiffornis_turdina_KU18445_PE_E
Schiffornis_turdina_LSUMZ42928_PE_E
Schiffornis_turdina_LSUMZ42453_PE_E
Schiffornis_turdina_LSUMZ42548_PE_E
Schiffornis_turdina_KUNHM889_PE_E
Schiffornis_turdina_LSUMZ7087_PE_E
Schiffornis_turdina_LSUMZ4179_PE_E
Schiffornis_turdina_LSUMNS2552_PE_E
Schiffornis_turdina_LSUMZ4321_PE_E
Schiffornis_turdina_LSUMZ4676_PE_E
Schiffornis_turdina_LSUMZ4163_PE_E
Schiffornis_turdina_MPEGAMZ161_BR_E
Schiffornis_turdina_LSUMZ7119_PE_E
Schiffornis_turdina_MPEGJAP816_BR_E
Schiffornis_turdina_LSUMZ4175_PE_E
Schiffornis_turdina_LSUMNS7550_VE_E
Schiffornis_turdina_MPEGJAP770_BR_E
Schiffornis_turdina_MPEGAMZ514_BR_E
Schiffornis_turdina_IAvH11033_CO_E
Schiffornis_turdina_IAvH11195_CO_E
Schiffornis_turdina_ANSP5792_EC_E
Eastern clade (BO, BR; 53 individuals)
Western clade (CR, EC HO, MX, PA, PE; 38 individuals)
Eastern clade (BR,GU; 68 individuals)
Laniocera_hypopyrra_KUNHM1408_OG
Laniisoma_elegans_ANSP1558_OG
1
0.68
1
1
1
1
0.43
1
35
30
25
20
15
10
5
0
Million years before present (Ma)

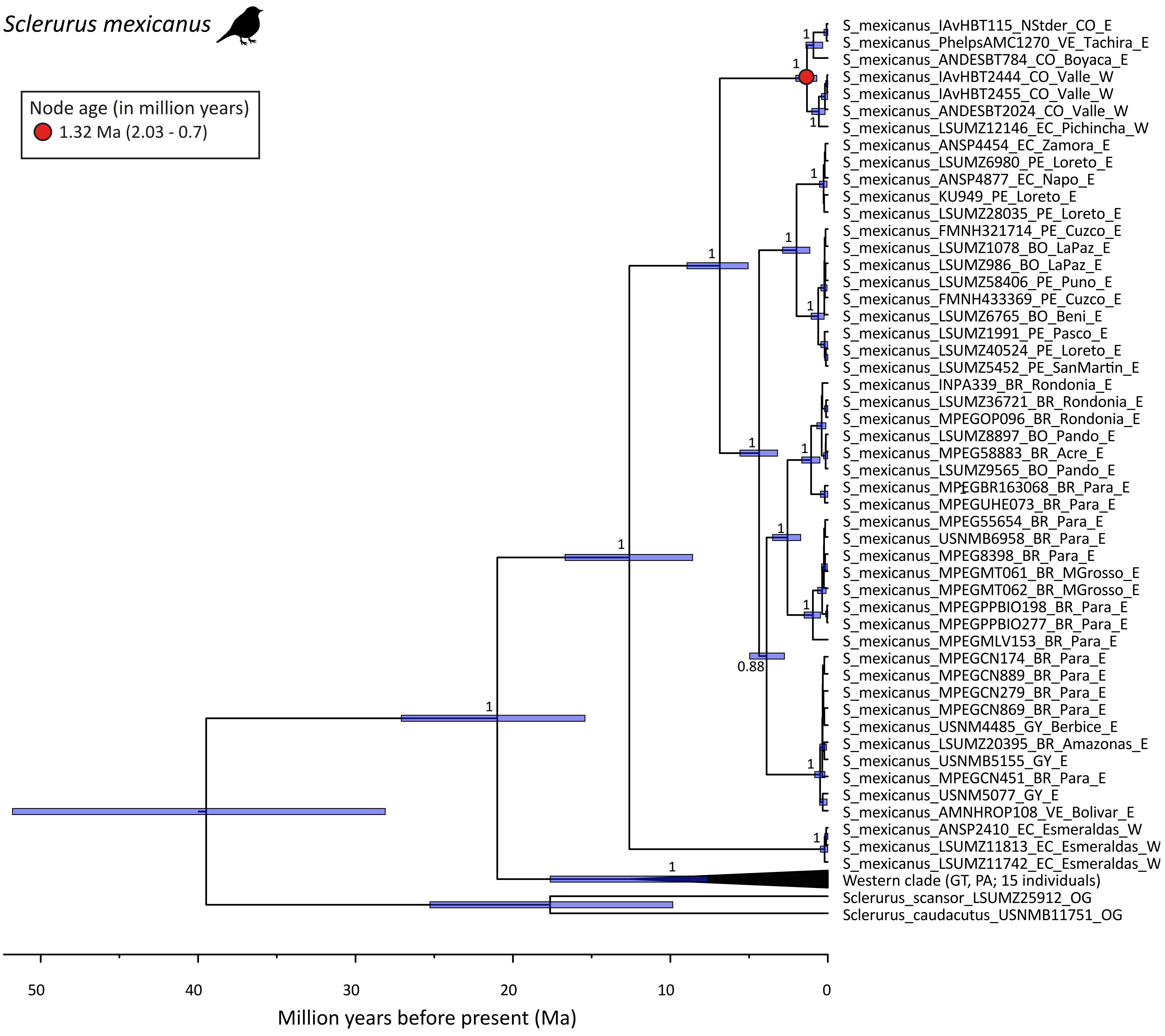

Sclerurus mexicanus
Node age (in million years)
1.32 Ma (2.03 - 0.7)
S_mexicanus_IAvHBT115_NStder_CO_E
S_mexicanus_PhelpsAMC1270_VE_Tachira_E
S_mexicanus_ANDESBT784_CO_Boyaca_E
S_mexicanus_IAvHBT2444_CO_Valle_W
S_mexicanus_IAvHBT2455_CO_Valle_W
S_mexicanus_ANDESBT2024_CO_Valle_W
S_mexicanus_LSUMZ12146_EC_Pichincha_W
S_mexicanus_ANSP4454_EC_Zamora_E
S_mexicanus_LSUMZ6980_PE_Loreto_E
S_mexicanus_ANSP4877_EC_Napo_E
S_mexicanus_KU949_PE_Loreto_E
S_mexicanus_LSUMZ28035_PE_Loreto_E
S_mexicanus_FMNH321714_PE_Cuzco_E
S_mexicanus_LSUMZ1078_BO_LaPaz_E
S_mexicanus_LSUMZ986_BO_LaPaz_E
S_mexicanus_LSUMZ58406_PE_Puno_E
S_mexicanus_FMNH433369_PE_Cuzco_E
S_mexicanus_LSUMZ6765_BO_Beni_E
S_mexicanus_LSUMZ1991_PE_Pasco_E
S_mexicanus_LSUMZ40524_PE_Loreto_E
S_mexicanus_LSUMZ5452_PE_SanMartin_E
S_mexicanus_INPA339_BR_Rondonia_E
S_mexicanus_LSUMZ36721_BR_Rondonia_E
S_mexicanus_MPEGOP096_BR_Rondonia_E
S_mexicanus_LSUMZ8897_BO_Pando_E
S_mexicanus_MPEG58883_BR_Acre_E
S_mexicanus_LSUMZ9565_BO_Pando_E
S_mexicanus_MPEGBR163068_BR_Para_E
S_mexicanus_MPEGUHE073_BR_Para_E
S_mexicanus_MPEG55654_BR_Para_E
S_mexicanus_USNMB6958_BR_Para_E
S_mexicanus_MPEG8398_BR_Para_E
S_mexicanus_MPEGMT061_BR_MGrosso_E
S_mexicanus_MPEGMT062_BR_MGrosso_E
S_mexicanus_MPEGPPBIO198_BR_Para_E
S_mexicanus_MPEGPPBIO277_BR_Para_E
S_mexicanus_MPEGMLV153_BR_Para_E
S_mexicanus_MPEGCN174_BR_Para_E
S_mexicanus_MPEGCN889_BR_Para_E
S_mexicanus_MPEGCN279_BR_Para_E
S_mexicanus_MPEGCN869_BR_Para_E
S_mexicanus_USNM4485_GY_Berbice_E
S_mexicanus_LSUMZ20395_BR_Amazonas_E
S_mexicanus_USNMB5155_GY_E
S_mexicanus_MPEGCN451_BR_Para_E
S_mexicanus_USNM5077_GY_E
S_mexicanus_AMNHROP108_VE_Bolivar_E
S_mexicanus_ANSP2410_EC_Esmeraldas_W
S_mexicanus_LSUMZ11813_EC_Esmeraldas_W
S_mexicanus_LSUMZ11742_EC_Esmeraldas_W
Western clade (GT, PA; 15 individuals)
Sclerurus_scansor_LSUMZ25912_OG
Sclerurus_caudacutus_USNMB11751_OG
0.88
50
40
30
20
10
0
Million years before present (Ma)

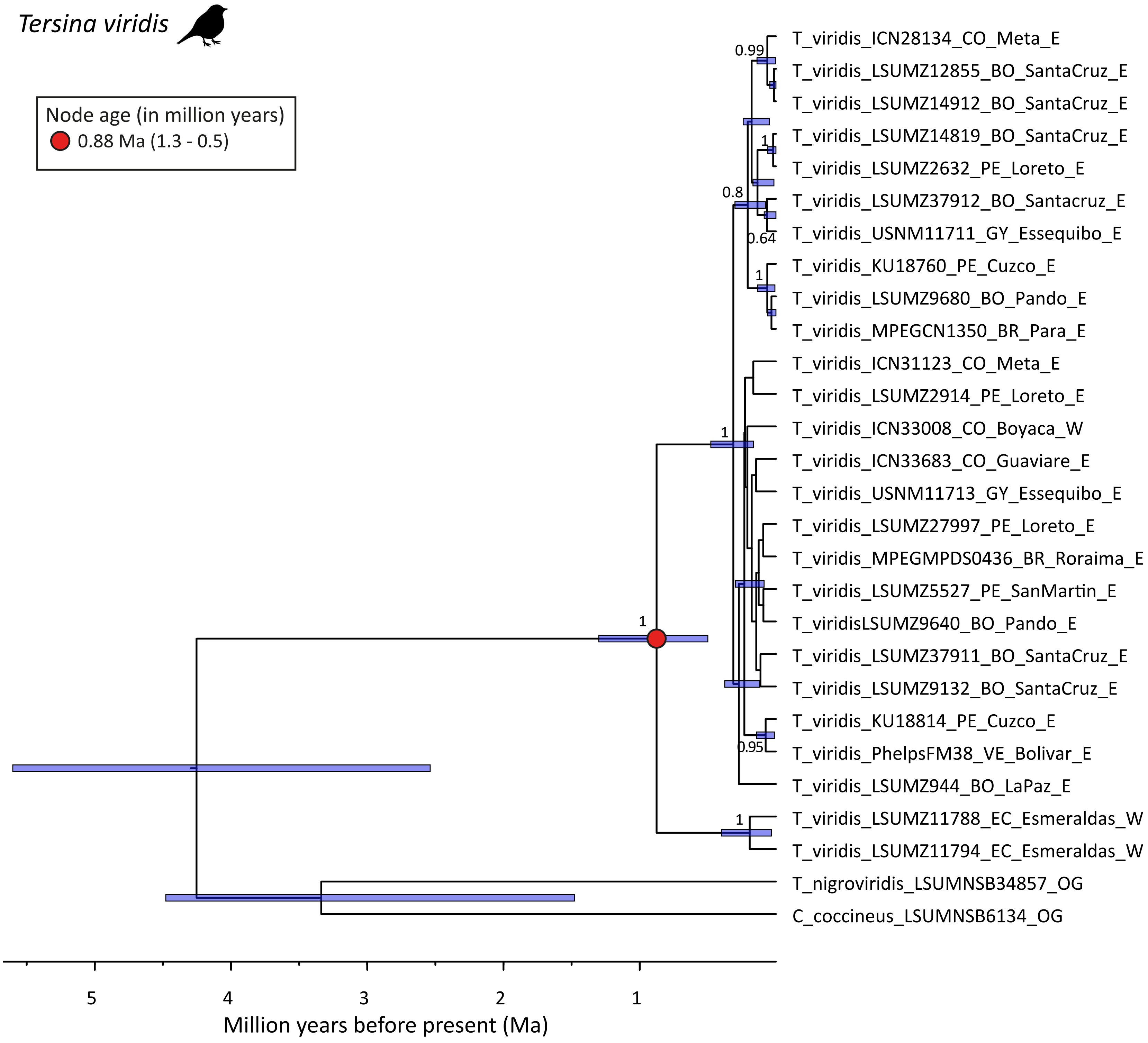

Tersina viridis
Node age (in million years)
0.88 Ma (1.3 - 0.5)
0.99
1
0.8
0.64
1
1
1
1
0.95
1
T_viridis_ICN28134_CO_Meta_E
T_viridis_LSUMZ12855_BO_SantaCruz_E
T_viridis_LSUMZ14912_BO_SantaCruz_E
T_viridis_LSUMZ14819_BO_SantaCruz_E
T_viridis_LSUMZ2632_PE_Loreto_E
T_viridis_LSUMZ37912_BO_Santacruz_E
T_viridis_USNM11711_GY_Essequibo_E
T_viridis_KU18760_PE_Cuzco_E
T_viridis_LSUMZ9680_BO_Pando_E
T_viridis_MPEGCN1350_BR_Para_E
T_viridis_ICN31123_CO_Meta_E
T_viridis_LSUMZ2914_PE_Loreto_E
T_viridis_ICN33008_CO_Boyaca_W
T_viridis_ICN33683_CO_Guaviare_E
T_viridis_USNM11713_GY_Essequibo_E
T_viridis_LSUMZ27997_PE_Loreto_E
T_viridis_MPEGMPDS0436_BR_Roraima_E
T_viridis_LSUMZ5527_PE_SanMartin_E
T_viridisLSUMZ9640_BO_Pando_E
T_viridis_LSUMZ37911_BO_SantaCruz_E
T_viridis_LSUMZ9132_BO_SantaCruz_E
T_viridis_KU18814_PE_Cuzco_E
T_viridis_PhelpsFM38_VE_Bolivar_E
T_viridis_LSUMZ944_BO_LaPaz_E
T_viridis_LSUMZ11788_EC_Esmeraldas_W
T_viridis_LSUMZ11794_EC_Esmeraldas_W
T_nigroviridis_LSUMNSB34857_OG
C_coccineus_LSUMNSB6134_OG
5
4
3
2
1
Million years before present (Ma)

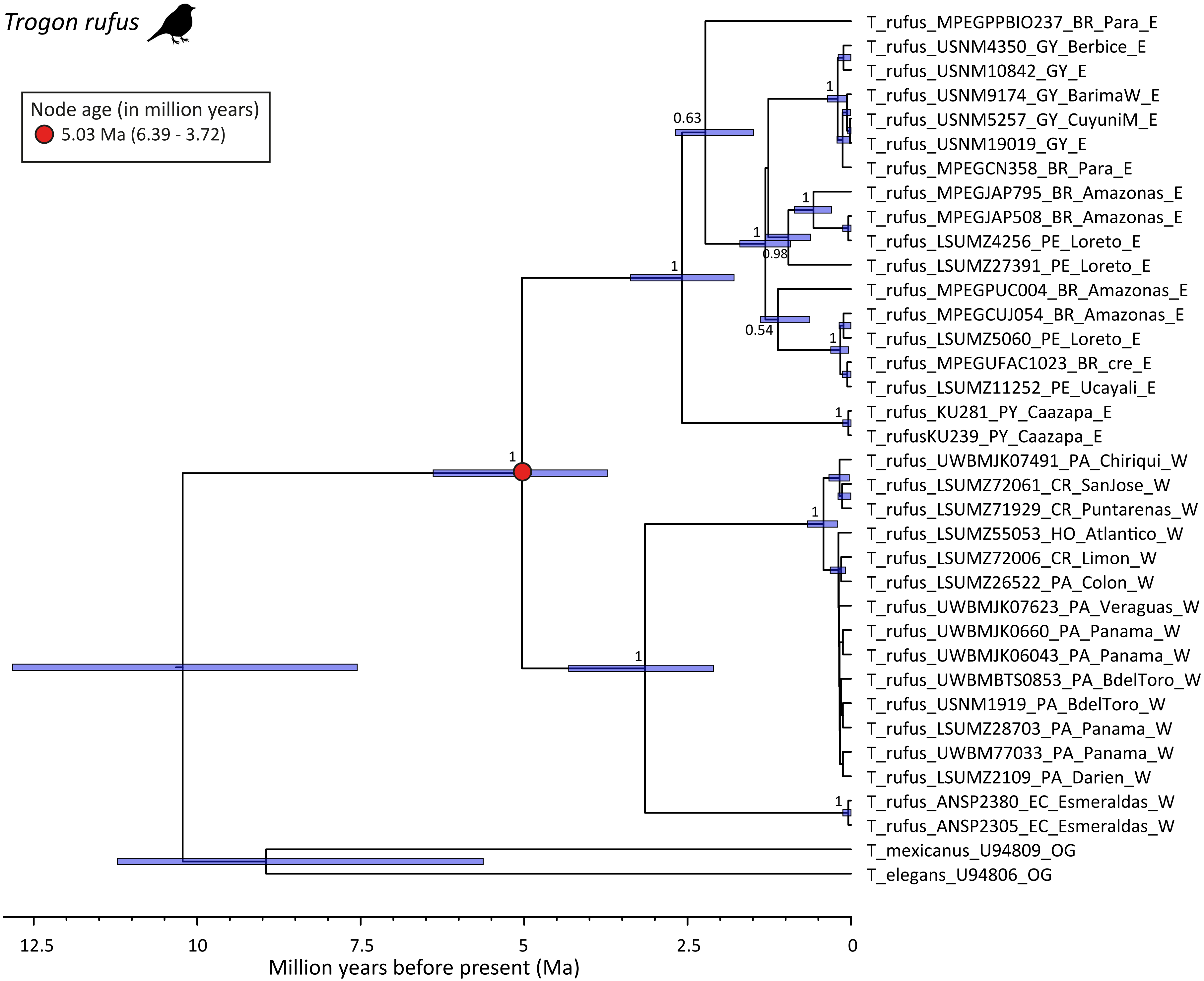
Trogon rufus
Node age (in million years)
5.03 Ma (6.39 - 3.72)
T_rufus_MPEGPPBIO237_BR_Para_E
T_rufus_USNM4350_GY_Berbice_E
T_rufus_USNM10842_GY_E
T_rufus_USNM9174_GY_BarimaW_E
T_rufus_USNM5257_GY_CuyuniM_E
T_rufus_USNM19019_GY_E
T_rufus_MPEGCN358_BR_Para_E
T_rufus_MPEGJAP795_BR_Amazonas_E
T_rufus_MPEGJAP508_BR_Amazonas_E
T_rufus_LSUMZ4256_PE_Loreto_E
T_rufus_LSUMZ27391_PE_Loreto_E
T_rufus_MPEGPUC004_BR_Amazonas_E
T_rufus_MPEGCUJ054_BR_Amazonas_E
T_rufus_LSUMZ5060_PE_Loreto_E
T_rufus_MPEGUFAC1023_BR_cre_E
T_rufus_LSUMZ11252_PE_Ucayali_E
T_rufus_KU281_PY_Caazapa_E
T_rufusKU239_PY_Caazapa_E
T_rufus_UWBMJK07491_PA_Chiriqui_W
T_rufus_LSUMZ72061_CR_SanJose_W
T_rufus_LSUMZ71929_CR_Puntarenas_W
T_rufus_LSUMZ55053_HO_Atlantico_W
T_rufus_LSUMZ72006_CR_Limon_W
T_rufus_LSUMZ26522_PA_Colon_W
T_rufus_UWBMJK07623_PA_Veraguas_W
T_rufus_UWBMJK0660_PA_Panama_W
T_rufus_UWBMJK06043_PA_Panama_W
T_rufus_UWBMBTS0853_PA_BdelToro_W
T_rufus_USNM1919_PA_BdelToro_W
T_rufus_LSUMZ28703_PA_Panama_W
T_rufus_UWBM77033_PA_Panama_W
T_rufus_LSUMZ2109_PA_Darien_W
T_rufus_ANSP2380_EC_Esmeraldas_W
T_rufus_ANSP2305_EC_Esmeraldas_W
T_mexicanus_U94809_OG
T_elegans_U94806_OG
0.63
1
0.98
0.54
12.5
10
7.5
5
2.5
0
Million years before present (Ma)

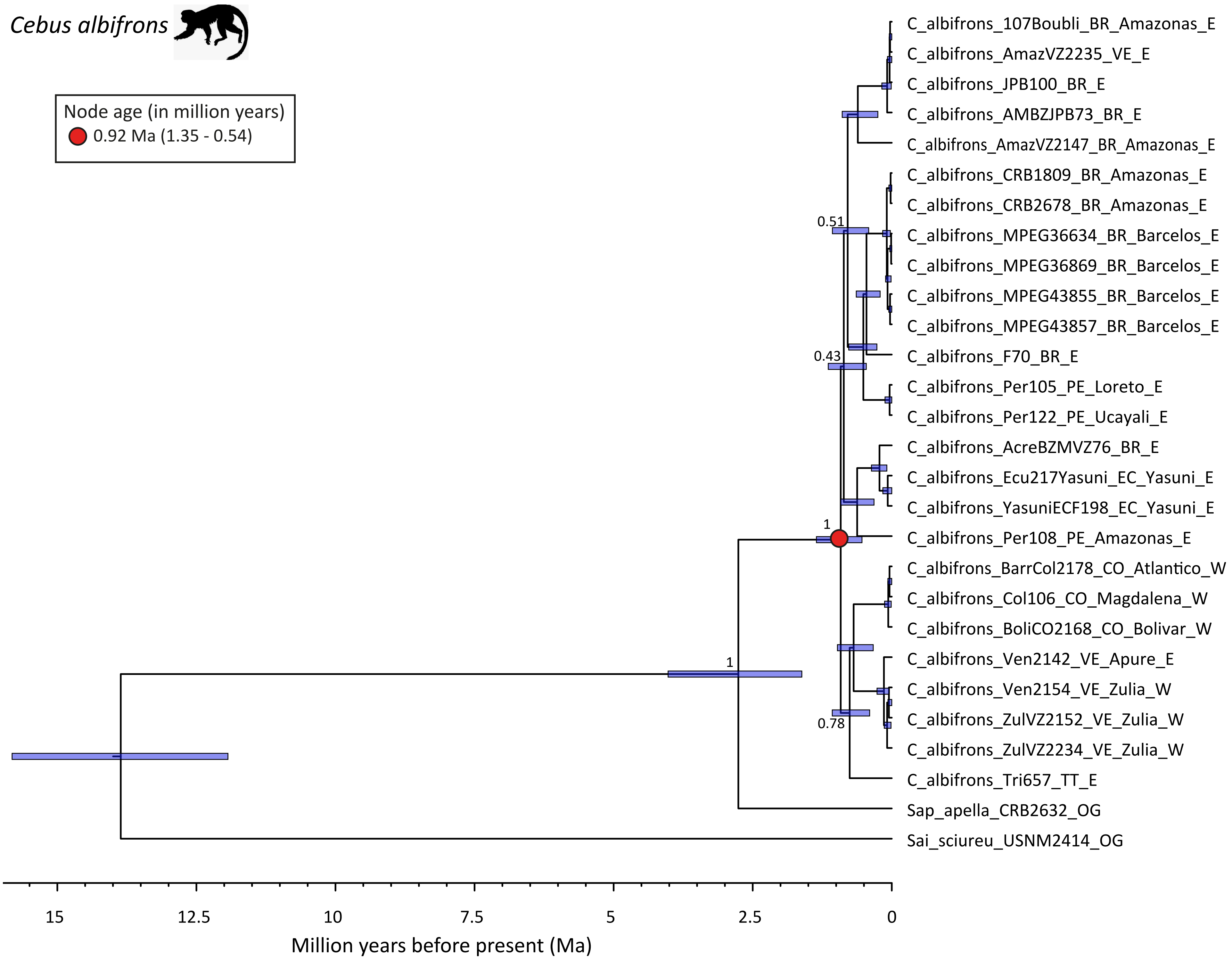

Cebus albifrons
Node age (in million years)
0.92 Ma (1.35 - 0.54)
C_albifrons_107Boubli_BR_Amazonas_E
C_albifrons_AmazVZ2235_VE_E
C_albifrons_JPB100_BR_E
C_albifrons_AMBZJPB73_BR_E
C_albifrons_AmazVZ2147_BR_Amazonas_E
C_albifrons_CRB1809_BR_Amazonas_E
C_albifrons_CRB2678_BR_Amazonas_E
C_albifrons_MPEG36634_BR_Barcelos_E
C_albifrons_MPEG36869_BR_Barcelos_E
C_albifrons_MPEG43855_BR_Barcelos_E
C_albifrons_MPEG43857_BR_Barcelos_E
C_albifrons_F70_BR_E
C_albifrons_Per105_PE_Loreto_E
C_albifrons_Per122_PE_Ucayali_E
C_albifrons_AcreBZMVZ76_BR_E
C_albifrons_Ecu217Yasuni_EC_Yasuni_E
C_albifrons_YasuniECF198_EC_Yasuni_E
C_albifrons_Per108_PE_Amazonas_E
C_albifrons_BarrCol2178_CO_Atlantico_W
C_albifrons_Col106_CO_Magdalena_W
C_albifrons_BoliCO2168_CO_Bolivar_W
C_albifrons_Ven2142_VE_Apure_E
C_albifrons_Ven2154_VE_Zulia_W
C_albifrons_ZulVZ2152_VE_Zulia_W
C_albifrons_ZulVZ2234_VE_Zulia_W
C_albifrons_Tri657_TT_E
Sap_apella_CRB2632_OG
Sai_sciureu_USNM2414_OG
0.51
0.43
1
1
0.78
15
12.5
10
7.5
5
2.5
0
Million years before present (Ma)

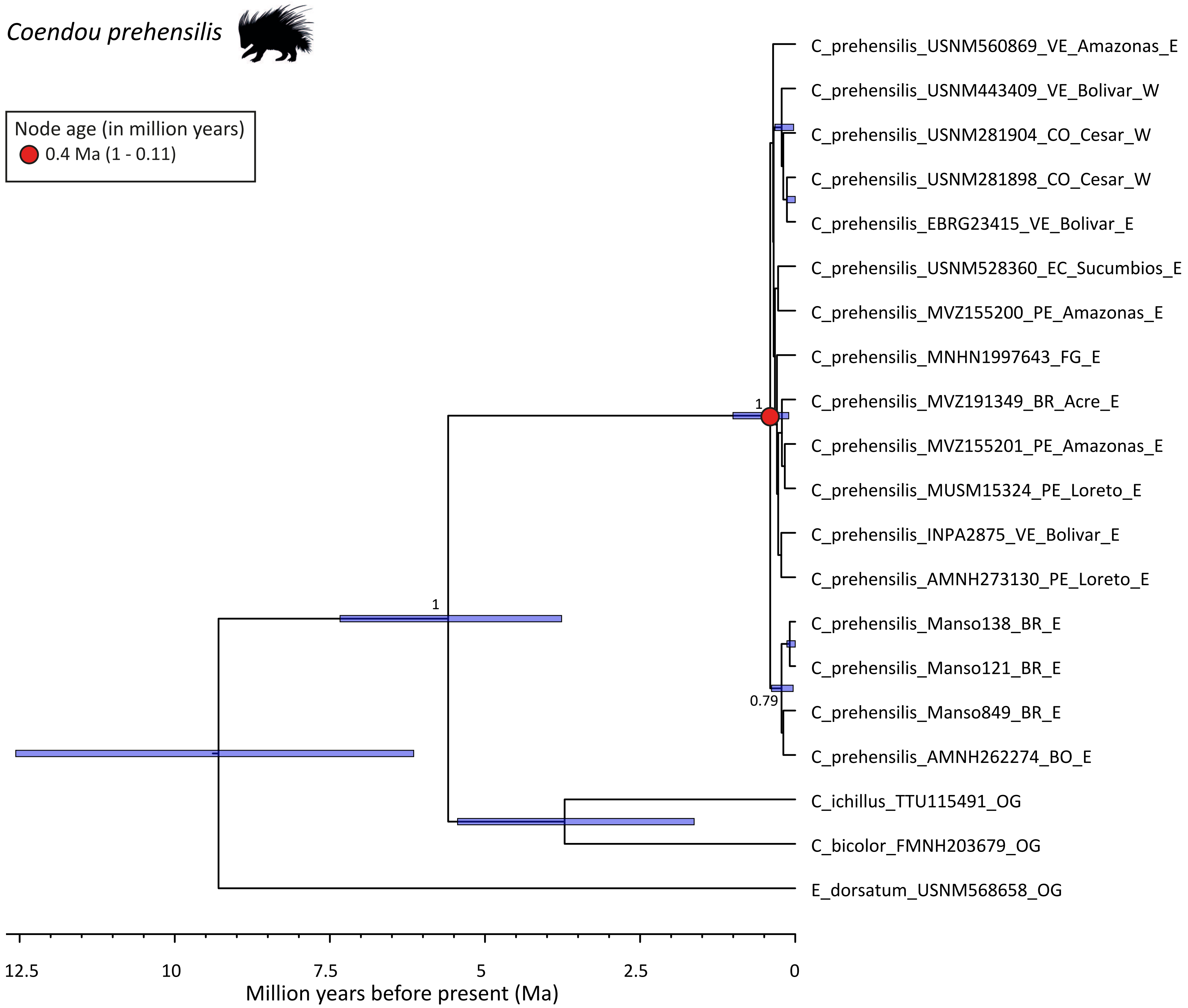
Coendou prehensilis
Node age (in million years)
0.4 Ma (1 - 0.11)
C_prehensilis_USNM560869_VE_Amazonas_E
C_prehensilis_USNM443409_VE_Bolivar_W
C_prehensilis_USNM281904_CO_Cesar_W
C_prehensilis_USNM281898_CO_Cesar_W
C_prehensilis_EBRG23415_VE_Bolivar_E
C_prehensilis_USNM528360_EC_Sucumbios_E
C_prehensilis_MVZ155200_PE_Amazonas_E
C_prehensilis_MNHN1997643_FG_E
C_prehensilis_MVZ191349_BR_Acre_E
C_prehensilis_MVZ155201_PE_Amazonas_E
C_prehensilis_MUSM15324_PE_Loreto_E
C_prehensilis_INPA2875_VE_Bolivar_E
C_prehensilis_AMNH273130_PE_Loreto_E
C_prehensilis_Manso138_BR_E
C_prehensilis_Manso121_BR_E
C_prehensilis_Manso849_BR_E
C_prehensilis_AMNH262274_BO_E
C_ichillus_TTU115491_OG
C_bicolor_FMNH203679_OG
E_dorsatum_USNM568658_OG
1
1
0.79
12.5
10
7.5
5
2.5
0
Million years before present (Ma)

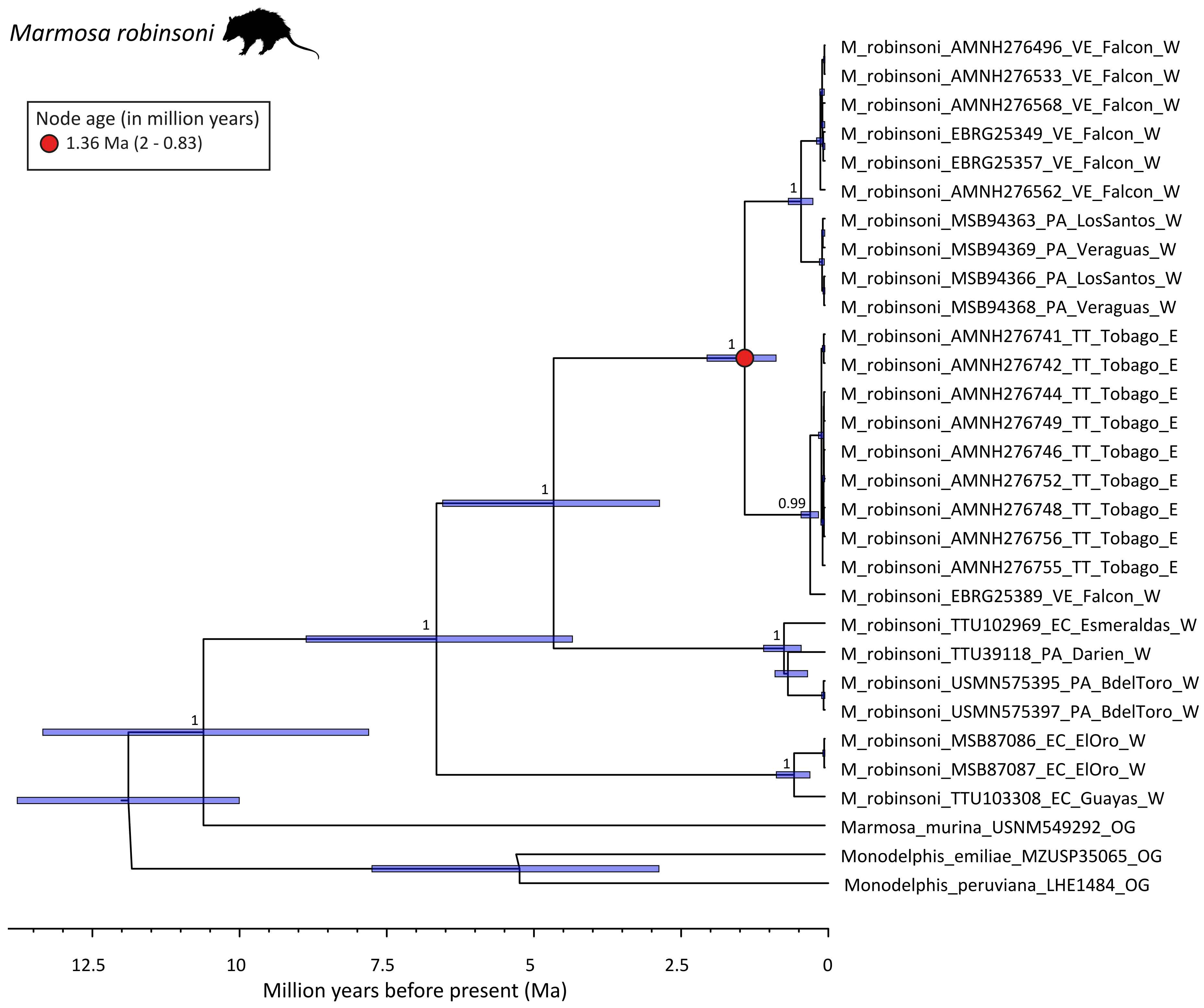

Marmosa robinsoni
Node age (in million years)
1.36 Ma (2 - 0.83)
M_robinsoni_AMNH276496_VE_Falcon_W
M_robinsoni_AMNH276533_VE_Falcon_W
M_robinsoni_AMNH276568_VE_Falcon_W
M_robinsoni_EBRG25349_VE_Falcon_W
M_robinsoni_EBRG25357_VE_Falcon_W
M_robinsoni_AMNH276562_VE_Falcon_W
M_robinsoni_MSB94363_PA_LosSantos_W
M_robinsoni_MSB94369_PA_Veraguas_W
M_robinsoni_MSB94366_PA_LosSantos_W
M_robinsoni_MSB94368_PA_Veraguas_W
M_robinsoni_AMNH276741_TT_Tobago_E
M_robinsoni_AMNH276742_TT_Tobago_E
M_robinsoni_AMNH276744_TT_Tobago_E
M_robinsoni_AMNH276749_TT_Tobago_E
M_robinsoni_AMNH276746_TT_Tobago_E
M_robinsoni_AMNH276752_TT_Tobago_E
M_robinsoni_AMNH276748_TT_Tobago_E
M_robinsoni_AMNH276756_TT_Tobago_E
M_robinsoni_AMNH276755_TT_Tobago_E
M_robinsoni_EBRG25389_VE_Falcon_W
M_robinsoni_TTU102969_EC_Esmeraldas_W
M_robinsoni_TTU39118_PA_Darien_W
M_robinsoni_USMN575395_PA_BdelToro_W
M_robinsoni_USMN575397_PA_BdelToro_W
M_robinsoni_MSB87086_EC_ElOro_W
M_robinsoni_MSB87087_EC_ElOro_W
M_robinsoni_TTU103308_EC_Guayas_W
Marmosa_murina_USNM549292_OG
Monodelphis_emiliae_MZUSP35065_OG
Monodelphis_peruviana_LHE1484_OG
1
1
0.99
1
1
1
1
1
1
12.5
10
7.5
5
2.5
0
Million years before present (Ma)

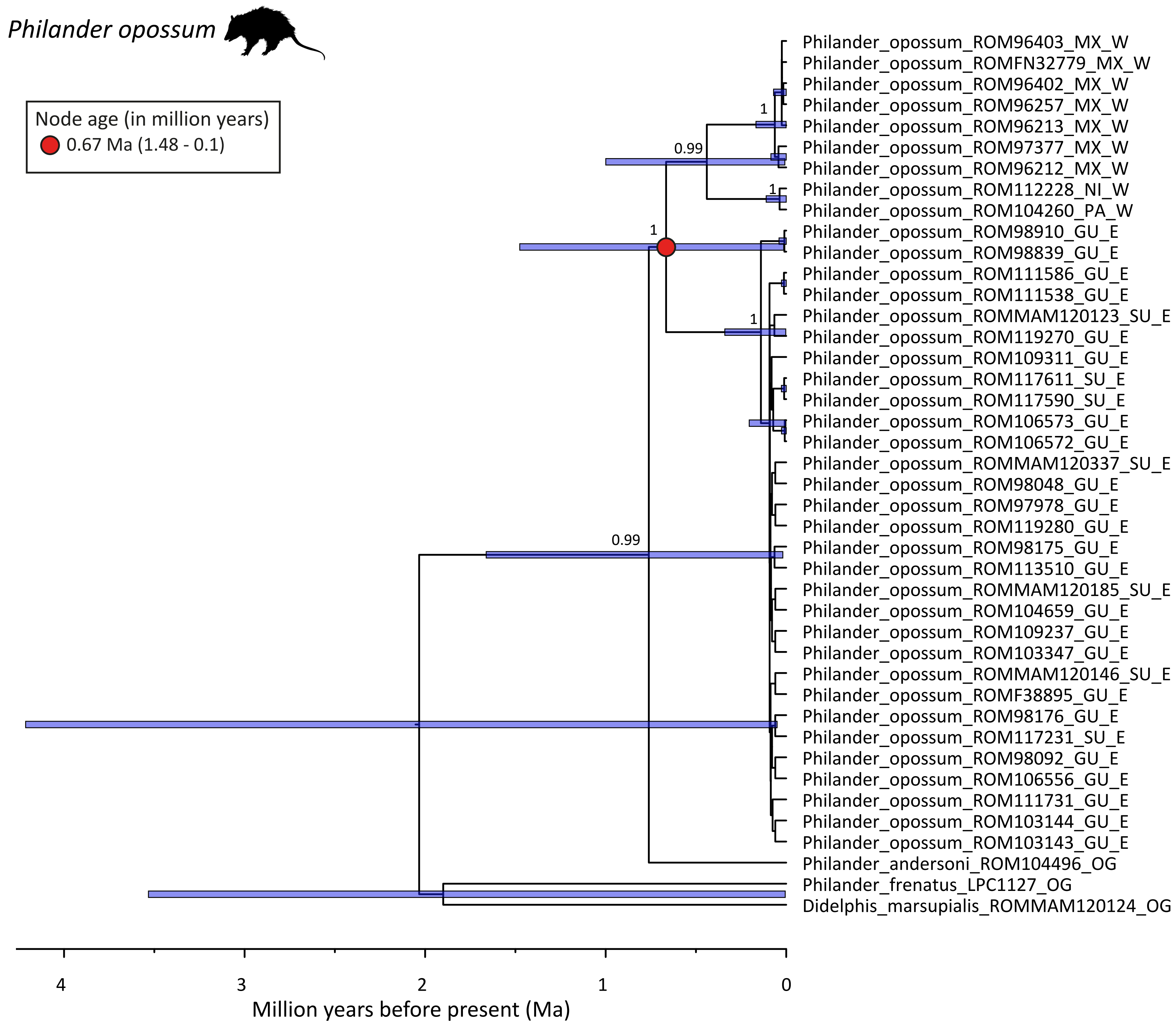

Philander opossum
Node age (in million years)
0.67 Ma (1.48 - 0.1)
Philander_opossum_ROM96403_MX_W
Philander_opossum_ROMFN32779_MX_W
Philander_opossum_ROM96402_MX_W
Philander_opossum_ROM96257_MX_W
Philander_opossum_ROM96213_MX_W
Philander_opossum_ROM97377_MX_W
Philander_opossum_ROM96212_MX_W
Philander_opossum_ROM112228_NI_W
Philander_opossum_ROM104260_PA_W
Philander_opossum_ROM98910_GU_E
Philander_opossum_ROM98839_GU_E
Philander_opossum_ROM111586_GU_E
Philander_opossum_ROM111538_GU_E
Philander_opossum_ROMMAM120123_SU_E
Philander_opossum_ROM119270_GU_E
Philander_opossum_ROM109311_GU_E
Philander_opossum_ROM117611_SU_E
Philander_opossum_ROM117590_SU_E
Philander_opossum_ROM106573_GU_E
Philander_opossum_ROM106572_GU_E
Philander_opossum_ROMMAM120337_SU_E
Philander_opossum_ROM98048_GU_E
Philander_opossum_ROM97978_GU_E
Philander_opossum_ROM119280_GU_E
Philander_opossum_ROM98175_GU_E
Philander_opossum_ROM113510_GU_E
Philander_opossum_ROMMAM120185_SU_E
Philander_opossum_ROM104659_GU_E
Philander_opossum_ROM109237_GU_E
Philander_opossum_ROM103347_GU_E
Philander_opossum_ROMMAM120146_SU_E
Philander_opossum_ROMF38895_GU_E
Philander_opossum_ROM98176_GU_E
Philander_opossum_ROM117231_SU_E
Philander_opossum_ROM98092_GU_E
Philander_opossum_ROM106556_GU_E
Philander_opossum_ROM111731_GU_E
Philander_opossum_ROM103144_GU_E
Philander_opossum_ROM103143_GU_E
Philander_andersoni_ROM104496_OG
Philander_frenatus_LPC1127_OG
Didelphis_marsupialis_ROMMAM120124_OG
1
0.99
1
1
1
0.99
4
3
2
1
0
Million years before present (Ma)

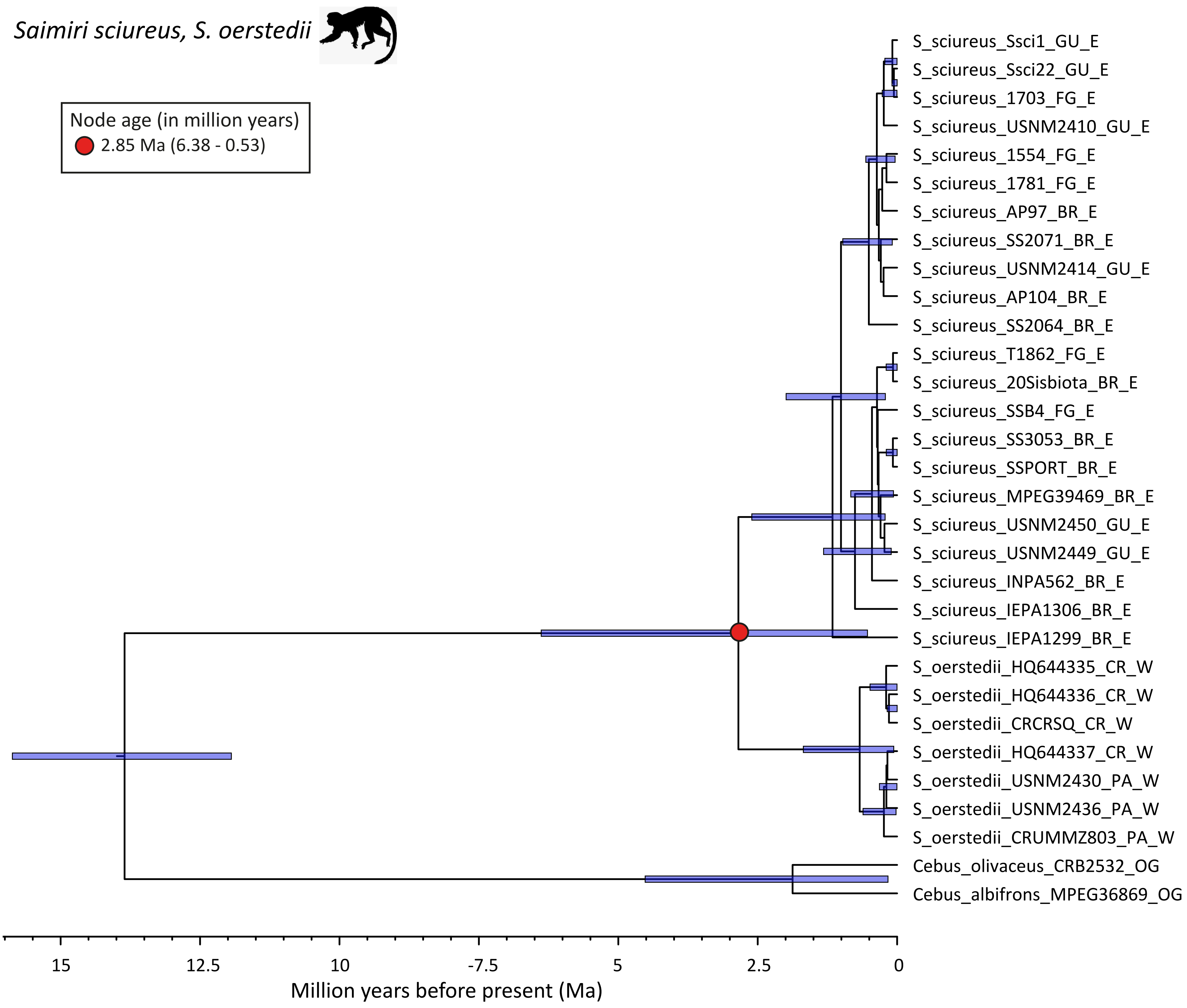

Saimiri sciureus, S. oerstedii
Node age (in million years)
2.85 Ma (6.38 - 0.53)
S_sciureus_Ssci1_GU_E
S_sciureus_Ssci22_GU_E
S_sciureus_1703_FG_E
S_sciureus_USNM2410_GU_E
S_sciureus_1554_FG_E
S_sciureus_1781_FG_E
S_sciureus_AP97_BR_E
S_sciureus_SS2071_BR_E
S_sciureus_USNM2414_GU_E
S_sciureus_AP104_BR_E
S_sciureus_SS2064_BR_E
S_sciureus_T1862_FG_E
S_sciureus_20Sisbiota_BR_E
S_sciureus_SSB4_FG_E
S_sciureus_SS3053_BR_E
S_sciureus_SSPORT_BR_E
S_sciureus_MPEG39469_BR_E
S_sciureus_USNM2450_GU_E
S_sciureus_USNM2449_GU_E
S_sciureus_INPA562_BR_E
S_sciureus_IEPA1306_BR_E
S_sciureus_IEPA1299_BR_E
S_oerstedii_HQ644335_CR_W
S_oerstedii_HQ644336_CR_W
S_oerstedii_CRCRSQ_CR_W
S_oerstedii_HQ644337_CR_W
S_oerstedii_USNM2430_PA_W
S_oerstedii_USNM2436_PA_W
S_oerstedii_CRUMMZ803_PA_W
Cebus_olivaceus_CRB2532_OG
Cebus_albifrons_MPEG36869_OG
15
12.5
10
-7.5
5
2.5
0
Million years before present (Ma)

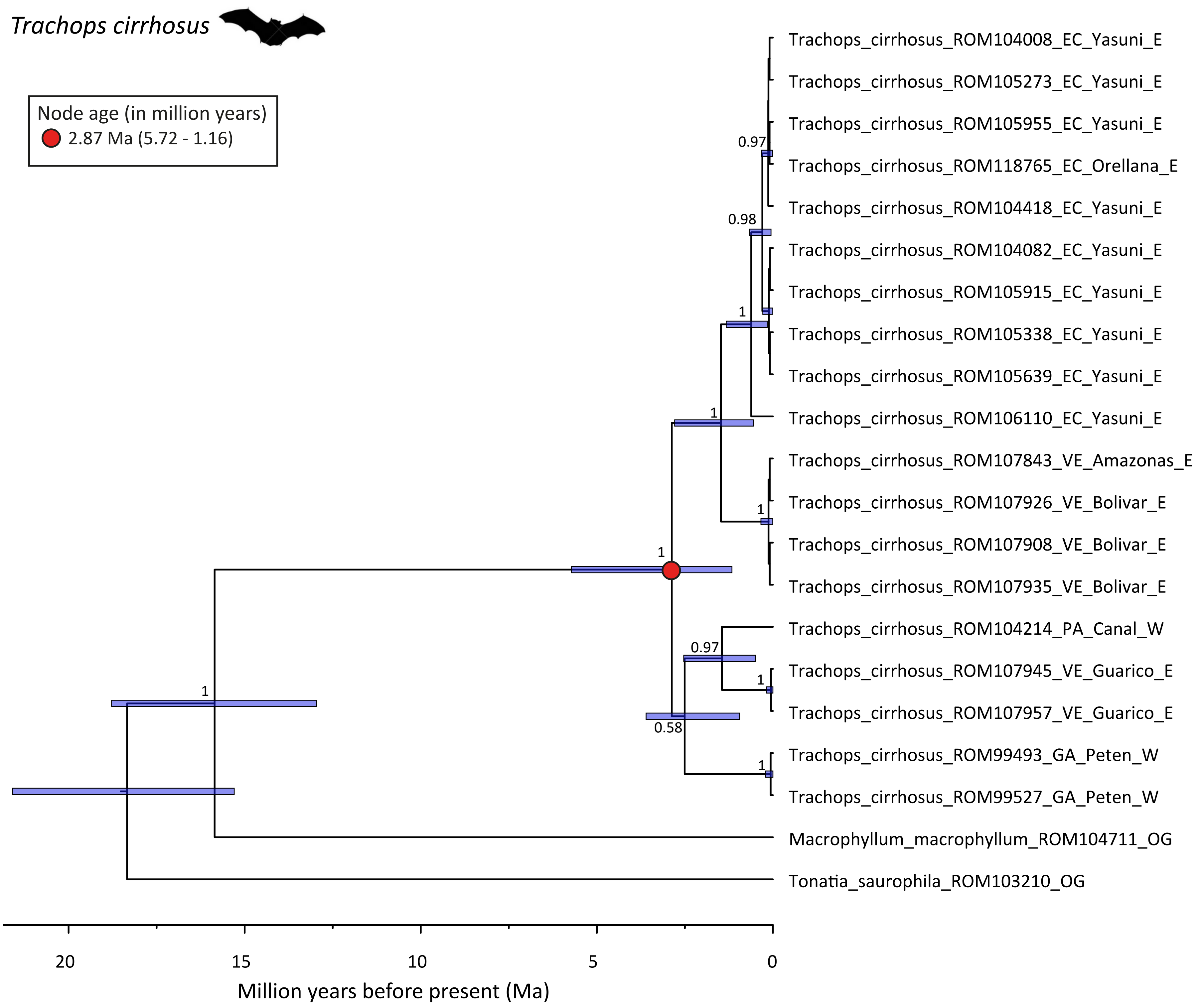
Trachops cirrhosus
Node age (in million years)
2.87 Ma (5.72 - 1.16)
Trachops_cirrhosus_ROM104008_EC_Yasuni_E
Trachops_cirrhosus_ROM105273_EC_Yasuni_E
Trachops_cirrhosus_ROM105955_EC_Yasuni_E
Trachops_cirrhosus_ROM118765_EC_Orellana_E
Trachops_cirrhosus_ROM104418_EC_Yasuni_E
Trachops_cirrhosus_ROM104082_EC_Yasuni_E
Trachops_cirrhosus_ROM105915_EC_Yasuni_E
Trachops_cirrhosus_ROM105338_EC_Yasuni_E
Trachops_cirrhosus_ROM105639_EC_Yasuni_E
Trachops_cirrhosus_ROM106110_EC_Yasuni_E
Trachops_cirrhosus_ROM107843_VE_Amazonas_E
Trachops_cirrhosus_ROM107926_VE_Bolivar_E
Trachops_cirrhosus_ROM107908_VE_Bolivar_E
Trachops_cirrhosus_ROM107935_VE_Bolivar_E
Trachops_cirrhosus_ROM104214_PA_Canal_W
Trachops_cirrhosus_ROM107945_VE_Guarico_E
Trachops_cirrhosus_ROM107957_VE_Guarico_E
Trachops_cirrhosus_ROM99493_GA_Peten_W
Trachops_cirrhosus_ROM99527_GA_Peten_W
Macrophyllum_macrophyllum_ROM104711_OG
Tonatia_saurophila_ROM103210_OG
0.97
0.98
1
1
1
1
0.97
1
0.58
1
1
20
15
10
5
0
Million years before present (Ma)
