# Supplementary material for: Synthesis of geological data and comparative phylogeography of lowland tetrapods suggests recent dispersal through lowland portals crossing the Eastern Andean Cordillera
Source: PeerJ. 2022 Jul 13;10:e13186. doi: 10.7717/peerj.13186 (PMC9288170; doi:10.7717/peerj.13186)
Supplement: Supplemental Information 5 [file peerj-10-13186-s005.docx]

Synthesis of geological data and comparative phylogeography of lowland tetrapods suggests recent dispersal through lowland portals

crossing the Eastern Andean Cordillera

Erika Rodríguez-Muñoz, Camilo Montes, Fernando J. M. Rojas-Runjaic, and Andrew J. Crawford.

**Appendix S3.** Substitution rates and generation times assumed for the MTML-msBayes analyses. Phylogeographic analyses assumed the HKY model of nucleotide substitution (Hasegawa *et al.*, 1985), a 2-rate model that also recognizes variation in nucleotide frequencies, two important aspects of mtDNA sequence evolution. As the number of possible divergence models increases when more taxa are added, the computational limits will undersample all the models when implementing a single analysis with the 37 lineages (Oaks et al., 2013), hence we ran one independent analysis for each class of tetrapods. The estimated number of divergence pulses (Ψ) is underestimated when τ prior is wider, so for each run we set the upper limit of τ to the oldest split age on each class according to our BEAST analysis. Divergence times estimated from MTML-msBayes are in coalescent units, thus the conversion to millions of years assumed roughly equal sex ratios, haploid and maternally inherited mtDNA, and was made following the equation *t =* τ*θ*_Ave_/*μ*, where *θ*_Ave_ /*μ* is the average effective population size for each class estimated by MTML-msBayes and *μ* is the neutral mutation rate per site per generation. For bird and amphibians we employed the same mean substitution rates assumed from BEAST analyses calibrations, and for reptiles and mammals we used specific substitution rates per genus according to Nabholz (2008) and Eo & DeWoody (2010). As a first approximation, we assumed *μ* can be estimated as the silent-site substitution rate (Kimura, 1968). We assumed generation time to be the age at which organisms reached their sexual maturity and obtained these data from the AnAge database (Tacutu *et al.*, 2018). Substitution rate and generation time assumed for each taxon, and their corresponding bibliographic source are given. Hyper-posteriors were estimated from 1,000 accepted draws from 1.5 million simulations. We made a local linear regression of the accepted parameter values obtained by the acceptance/rejection step in order to improve the posterior estimation. We used Bayes factors (BF; Kass & Raftery, 1995) to evaluate the relative posterior support for the number of divergence pulses. To estimate the timing of each divergence interval and the species contained in each, we constrained Ψ to the value with maximum BF and repeated the analysis as outlined above (Paz et al. 2015).

| Taxon | Total substitution rate (%) | Gene(s) used to estimate substitution rates | Taxon used to calibrate | Generation time (years) | Reference: Substitution rates | Reference: Generation times |
| --- | --- | --- | --- | --- | --- | --- |
| Tropical frogs | 1.91 | ND2 | Craugastor | 1 | (Crawford, 2003) | (Duellman & Trueb, 1986) |
| Birds | 2.1 | Cyt *b* |  | 1 | (Weir & Schluter, 2008) | (Chatterjee, 2015) |
| *Chelonoidis carbonaria* | 0.40 | Complete mitochondrial genome | Sauropsids | 5 | (Eo & DeWoody, 2010) | (Myers *et al.*, 2018) |
| *Boa constrictor* | 1.06 | Complete mitochondrial genome | Sauropsids | 3 | (Eo & DeWoody, 2010) | (Tacutu *et al.*, 2018) |
| *Crotalus durissus* | 1.06 | Complete mitochondrial genome | Sauropsids | 3 | (Eo & DeWoody, 2010) | (Tacutu *et al.*, 2018) |
| *Leptodeira* | 1.06 | Complete mitochondrial genome | Sauropsids | 2 | (Eo & DeWoody, 2010) | (Savage, 2002) |
| *Caiman crocodilus* | 0.08 | Whole genome | *Alligator mississippiensis, Crocodylus porosus, Gavialis gangeticus* | 6 | (Green *et al.*, 2014) | (Myers *et al.*, 2018) |
| *Cebus albifrons* | 3.5 | Cyt *b* |  | 3.6 | (Nabholz, Glémin, & Galtier, 2008) | (Tacutu *et al.*, 2018) |
| *Coendou prehensilis* | 6.2 | Cyt *b* |  | 1.6 | (Nabholz, Glémin, & Galtier, 2008) | (Tacutu *et al.*, 2018) |
| *Marmosa robinsoni* | 8.3 | Cyt *b* | Didelphimorphia/Australidephian | 0.9 | (Nabholz, Glémin, & Galtier, 2008) | (Tacutu *et al.*, 2018) |
| *Philander opossum* | 8.3 | Cyt *b* | Didelphimorphia/Australidephian | 1.2 | (Nabholz, Glémin, & Galtier, 2008) | (Tacutu *et al.*, 2018) |
| *Saimiri sciureus* | 8.8 | Cyt *b* |  | 3.9 | (Nabholz, Glémin, & Galtier, 2008) | (Tacutu *et al.*, 2018) |
| *Trachops cirrhosus* | 5.5 | Cyt *b* |  | 1 | (Nabholz, Glémin, & Galtier, 2008) | (Kunz & Fenton, 2005) |

Chatterjee, S. (2015). *The Rise of Birds: 225 Million Years of Evolution*. (S. Chatterjee, Ed.). Johns Hopkins University Press. Baltimore, Maryland.

Crawford, A. J. (2003). Relative rates of nucleotide substitution in frogs. *Journal of Molecular Evolution*, *57*(6), 636–41. https://doi.org/10.1007/s00239-003-2513-7

Duellman, W. E., & Trueb, L. (1986) Biology of the Amphibia. McGraw-Hill Book Co. New York, New York.

Eo, S. H., & DeWoody, J. A. (2010). Evolutionary rates of mitochondrial genomes correspond to diversification rates and to contemporary species richness in birds and reptiles. *Proceedings of the Royal Society B: Biological Sciences*, *277*(1700), 3587–3592. https://doi.org/10.1098/rspb.2010.0965

Green, R. E., Braun, E. L., Armstrong, J., Earl, D., Nguyen, N., Hickey, G., … Ray, D. A. (2014). Three crocodilian genomes reveal ancestral patterns of evolution among archosaurs. *Science*, *346*(6215), 1254449–1254449. https://doi.org/10.1126/science.1254449

Kunz, T. H., & Fenton, M. B. (2005). *Bat Ecology*. (T. H. Kunz & M. B. Fenton, Eds.). The University of Chicago Press. Chicago, Illinois.

Myers, P., Espinosa, R., Parr, C. S., Jones, T., Hammond, G. S., & Dewey, T. A. (2018). The Animal Diversity Web.

Nabholz, B., Glémin, S., & Galtier, N. (2008). Strong variations of mitochondrial mutation rate across mammals - The longevity hypothesis. *Molecular Biology and Evolution*, *25*(1), 120–130. https://doi.org/10.1093/molbev/msm248

Savage, J. M. (2002). *The Amphibians and Reptiles of Costa Rica: A Herpetofauna Between Two Continents, Between Two Seas*. The University of Chicago Press. Chicago, Illinois.

Tacutu, R., Thornton, D., Johnson, E., Budovsky, A., Barardo, Di., Craig, T., … De Magalhães, J. P. (2018). Human Ageing Genomic Resources: New and updated databases. *Nucleic Acids Research*, *46*(D1), D1083–D1090. https://doi.org/10.1093/nar/gkx1042

Weir, J. T., & Schluter, D. (2008). Calibrating the avian molecular clock. *Molecular Ecology*, *17*(10), 2321–2328. https://doi.org/10.1111/j.1365-294X.2008.03742.x
