# Supplementary material for: Synthesis of geological data and comparative phylogeography of lowland tetrapods suggests recent dispersal through lowland portals crossing the Eastern Andean Cordillera
Source: PeerJ. 2022 Jul 13;10:e13186. doi: 10.7717/peerj.13186 (PMC9288170; doi:10.7717/peerj.13186)
Supplement: Supplemental Information 6 [file peerj-10-13186-s006.docx]

Synthesis of geological and comparative phylogeographic data rejects mountain uplift as driver of lowland divergence across the Eastern Andean Cordillera.

Erika Rodríguez-Muñoz, Camilo Montes, Fernando J. M. Rojas-Runjaic, and Andrew J. Crawford.

**Appendix S4.**

**Figure 1.** Number of divergence intervals (Ψ) estimated by hABC for a) amphibians, b) non-avian reptiles, c) birds and d) mammals. Light gray bars represent the prior distribution (uniform), gray and black bars represent the posterior probability distribution, and for each class the black bar represents the scenario with the highest posterior probability.

a)


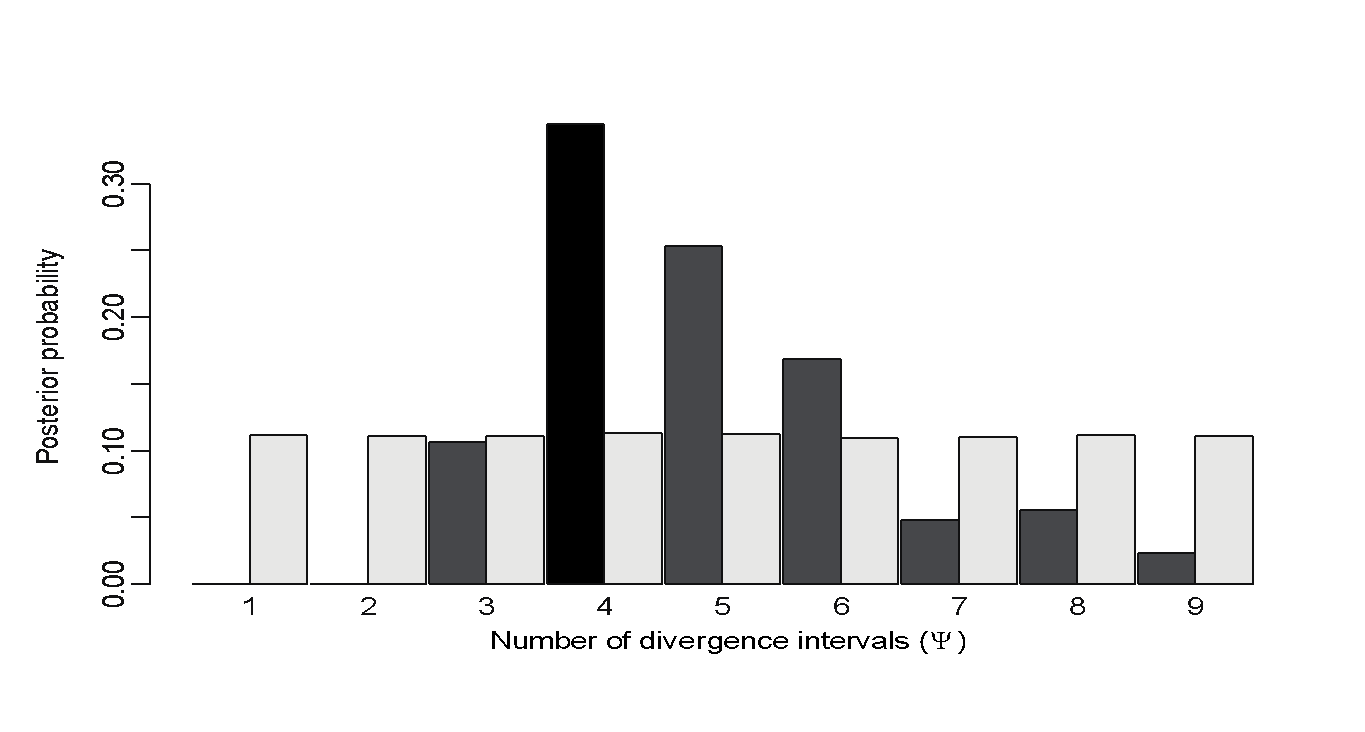


b)


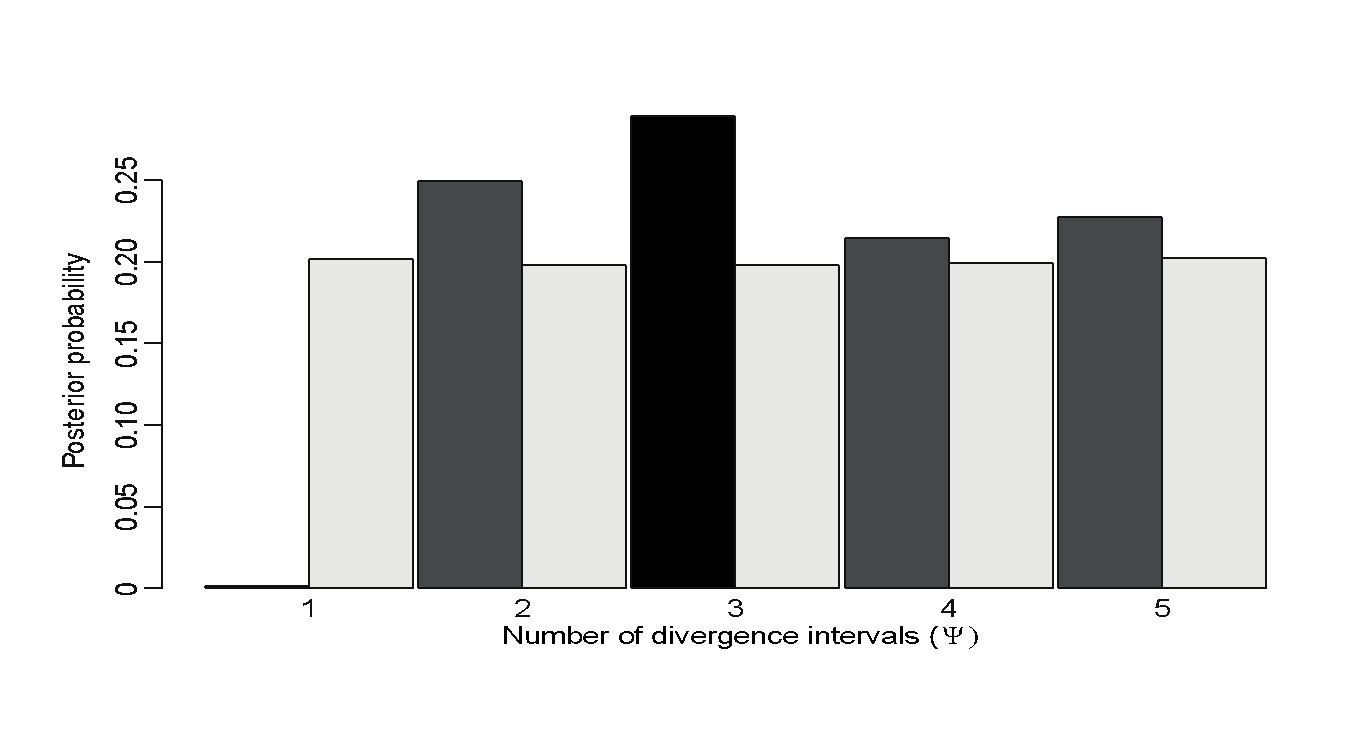


c)


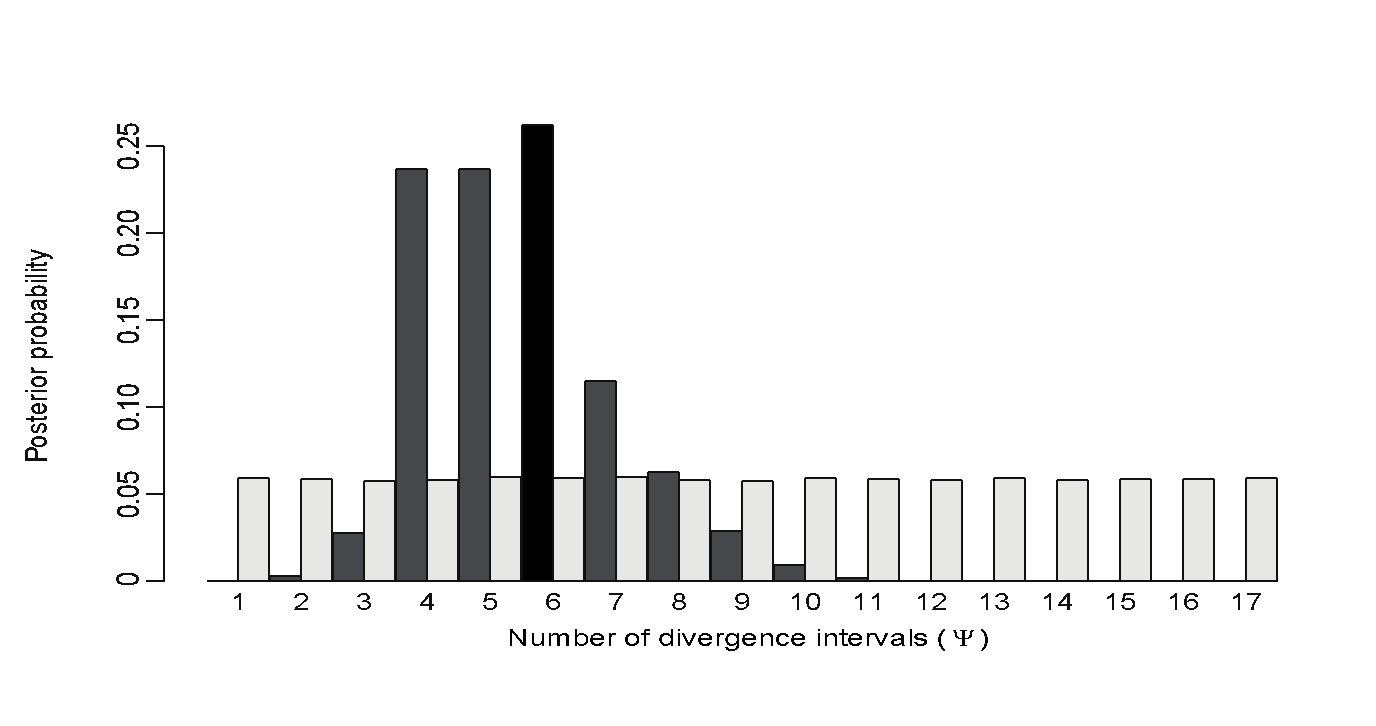


d)


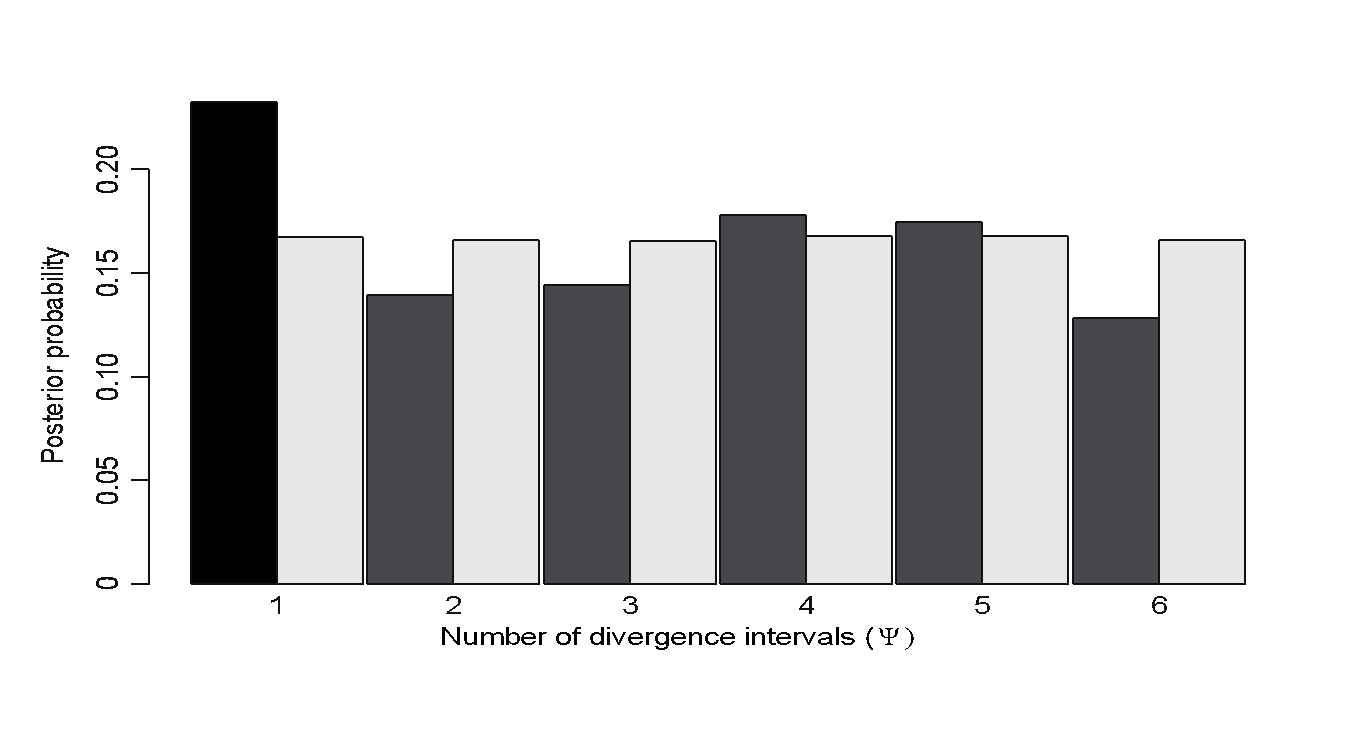


**Figure 2.**  Node ages estimated by BEAST against the average pairwise net difference between eastern and western populations, π_net_ for a) amphibians, b) non-avian reptiles, c) birds and d) mammals.

a)

**
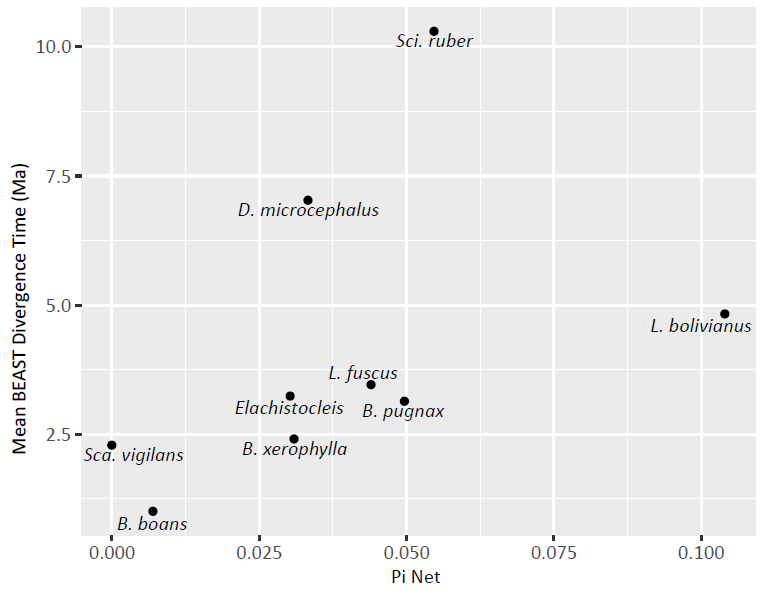
**

b)

**
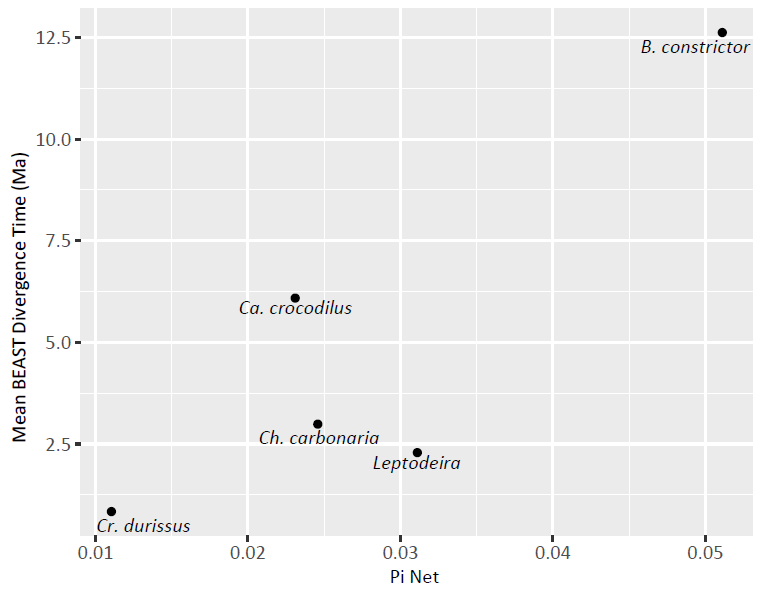
**

c)

**
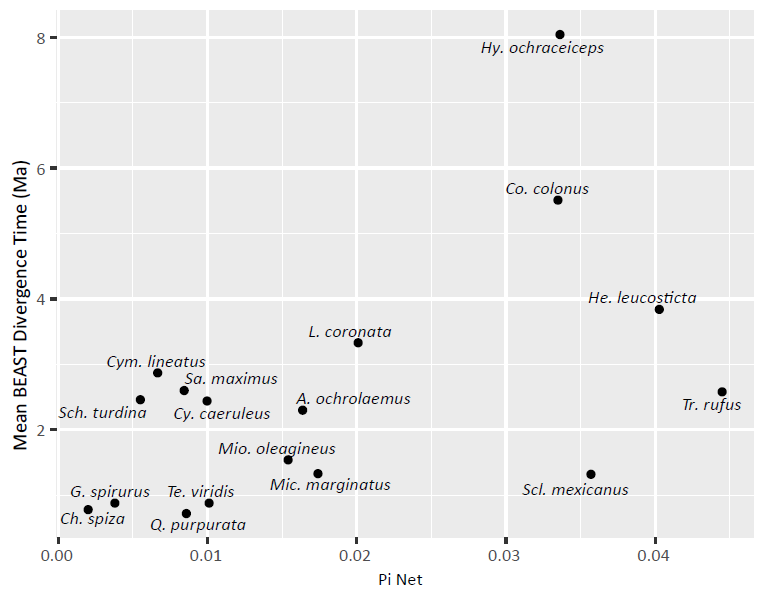
**

d)

**
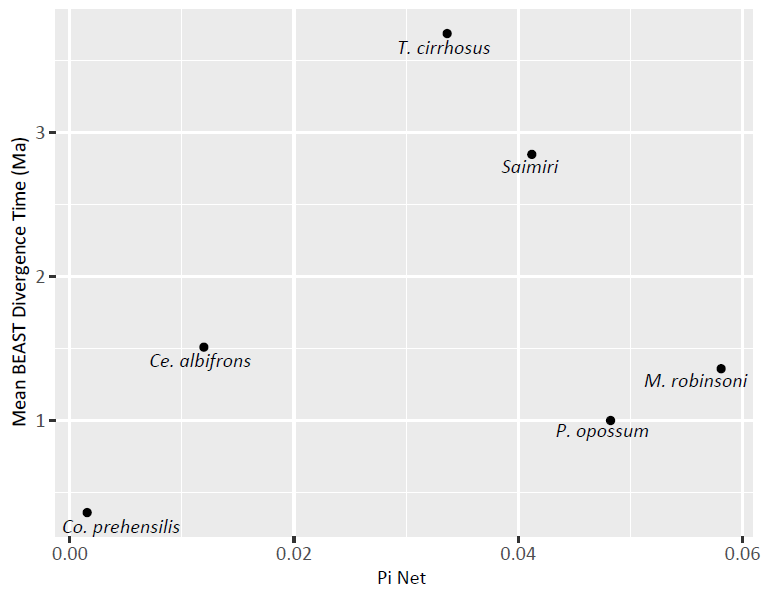
**

**Table 1.** Divergence times in million years before present (Ma) estimated for all lineages by each approximation (MTML-msBayes and BEAST).

| **Class** | **Species** | **Mean BEAST divergence (Ma)** | **Mean msBayes divergence (Ma)** |
| --- | --- | --- | --- |
| Amphibians | *Boana pugnax* | 3.14 | 3.62 |
| Amphibians | *Boana xerophylla* | 2.41 | 2.14 |
| Amphibians | *Boana boans* | 1.09 | 0.13 |
| Amphibians | *Dendropsophus microcephalus* | 7.03 | 3.62 |
| Amphibians | *Elachistocleis* | 3.26 | 2.14 |
| Amphibians | *Leptodactylus bolivianus* | 4.83 | 7.57 |
| Amphibians | *Leptodactylus fuscus* | 3.46 | 2.14 |
| Amphibians | *Scarthyla vigilans* | 2.29 | 0.13 |
| Amphibians | *Scinax ruber* | 10.3 | 7.57 |
| Birds | *Automolus ochrolaemus* | 2.3 | 2.67 |
| Birds | *Chlorophanes spiza* | 0.78 | 0.23 |
| Birds | *Colonia colonus* | 5.51 | 1.53 |
| Birds | *Cyanerpes caeruleus* | 2.44 | 0.65 |
| Birds | *Cymbilaimus lineatus* | 2.87 | 0.65 |
| Birds | *Glyphorynchus spirurus* | 2.84 | 2.1 |
| Birds | *Henicorrhina leucosticta* | 3.84 | 2.1 |
| Birds | *Hylophilus ochraceiceps* | 8.04 | 7.73 |
| Birds | *Lepidothrix coronata* | 3.33 | 1.53 |
| Birds | *Microcerculus marginatus* | 2.68 | 2.67 |
| Birds | *Mionectes oleagineus* | 1.54 | 0.65 |
| Birds | *Querula purpurata* | 0.72 | 0.23 |
| Birds | *Sclerurus mexicanus* | 1.32 | 0.23 |
| Birds | *Saltator maximus* | 2.6 | 1.53 |
| Birds | *Schiffornis turdina* | 2.07 | 2.1 |
| Birds | *Tersina viridis* | 0.88 | 0.23 |
| Birds | *Trogon rufus* | 2.58 | 2.67 |
| Mammals | *Cebus albifrons* | 1.51 | 0.36 |
| Mammals | *Coendou prehensilis* | 0.36 | 0.36 |
| Mammals | *Marmosa robinsoni* | 1.36 | 0.36 |
| Mammals | *Philander opossum* | 1 | 0.36 |
| Mammals | *Saimiri* | 2.85 | 0.36 |
| Mammals | *Trachops cirrhosus* | 3.69 | 0.36 |
| Reptiles | *Boa constrictor* | 12.62 | 9.05 |
| Reptiles | *Caiman crocodilus* | 6.09 | 4.59 |
| Reptiles | *Chelonoidis carbonaria* | 2.99 | 1.9 |
| Reptiles | *Crotalus durissus* | 0.84 | 1.9 |
| Reptiles | *Leptodeira* | 2.29 | 9.05 |
